# Supplementary material for: Occurrence and transmission potential of asymptomatic and presymptomatic SARS-CoV-2 infections: Update of a living systematic review and meta-analysis
Source: PLoS Med. 2022 May 26;19(5):e1003987. doi: 10.1371/journal.pmed.1003987 (PMC9135333; doi:10.1371/journal.pmed.1003987)
Supplement: S1 Text — (PDF) [file pmed.1003987.s002.pdf]

S1 Text. Search strings

From: Living Evidence on COVID-19 (https://ispmbern.github.io/covid-19/living-review/collectingdata.html, accessed 06.07.2021)

We retrieve data from EMBASE via OVID, MEDLINE via PubMed, BioRxiv and MedRxiv.

Search terms

When searches are updated, references that are identified that were not in the database before, are inserted by date (**date_entrez**) they were indexed in remote database, the date they are inserted in OUR database is formatted as the '**strategydate**' (raw data is available here).

01.05.2020

EMBASE:

```
(SARS coronavirus/ or middle east respiratory syndrome/ or severe acute
respiratory syndrome/ or (coronavirus* or corona virus* or HCoV* or ncov*
or covid or covid19 or sars-cov* or sarscov* or Sars-coronavirus* or Severe
Acute Respiratory Syndrome Coronavirus*).mp.) and 20191201:20301231.(dc).
```

29.04.2020

MEDLINE:

```
("coronavirus"[MH] OR "coronavirus infections"[MH] OR "coronavirus"[TW] OR
"corona virus"[TW] OR "HCoV"[TW] OR "nCov"[TW] OR "covid"[TW] OR
"covid19"[TW] OR "Severe Acute Respiratory Syndrome Coronavirus 2"[TW] OR
"SARS-CoV2"[TW] OR "SARS-CoV 2"[TW] OR "SARS Coronavirus 2"[TW] OR "MERS-
CoV"[TW]) AND (2019/1/1:3000[PDAT])
```

01.04.2020

From 01.04.2020, we retrieve the currate BioRxiv/MedRxiv dataset Link

26.03.2020

MEDLINE:

```
("Wuhan coronavirus" [Supplementary Concept] OR "COVID-19" OR SARS-CoV-2 OR
"2019 ncov"[tiab] OR (("novel coronavirus"[tiab] OR "new
coronavirus"[tiab]) AND (wuhan[tiab] OR 2019[tiab])) OR 2019-nCoV[All
Fields] OR (wuhan[tiab] AND coronavirus[tiab]))
```

EMBASE:

```
(nCoV or 2019-nCoV or ((new or novel or wuhan) adj3 coronavirus) or covid19
or covid-19 or SARS-CoV-2).mp.
```

BioRxiv/MedRxiv:

```
ncov or corona or wuhan or COVID or SARS-CoV-2
```

With the kind support of the Public Health & Primary Care Library PHC, and following guidance of the Medical Library Association

01.01.2020

MEDLINE:

```
("Wuhan coronavirus" [Supplementary Concept] OR "COVID-19" OR "2019
ncov"[tiab] OR (("novel coronavirus"[tiab] OR "new coronavirus"[tiab]) AND
(wuhan[tiab] OR 2019[tiab])) OR 2019-nCoV[All Fields] OR (wuhan[tiab] AND
coronavirus[tiab])))))
```

EMBASE:

```
ncov OR (wuhan AND corona) OR COVID
```

BioRxiv/MedRxiv:

```
ncov or corona or wuhan or COVID
```

We retained publications that used the keywords listed below in the title or abstract.

"asymp*" OR "pre-symp*" OR "presymp*" OR "preclinical" OR "pre-clinical" OR "without symptoms" OR "no symptoms" OR "free of symptoms" OR "non-symp*" OR "nonsymp*" OR "symptom-free" OR "symptomfree"
