# Supplementary material for: Occurrence and transmission potential of asymptomatic and presymptomatic SARS-CoV-2 infections: Update of a living systematic review and meta-analysis
Source: PLoS Med. 2022 May 26;19(5):e1003987. doi: 10.1371/journal.pmed.1003987 (PMC9135333; doi:10.1371/journal.pmed.1003987)
Supplement: S2 Text — (PDF) [file pmed.1003987.s003.pdf]

S2 Text. Risk of Bias Tool

<table>
<tr><td colspan="3">RISK OF BIAS TOOL-ASYMPTOMATIC REVIEW</td></tr>
<tr><td colspan="3">Selection Bias</td></tr>
<tr><td colspan="3"><u>Reporting:</u> How was the target population described? (Extract it from the study)
Target population: The collection of individuals, items, measurements, etc., about which inferences are desired (Porta, 2014). For our review, our target population is comprised of all people or participants who are at risk of getting SARS-CoV-2, and after positive RT-PCR, they are followed up to assess if they develop symptoms or remain asymptomatic during infection.</td></tr>
<tr><td colspan="3">Question 1- Was the sample invited to participate a close or true representation of the target population?</td></tr>
<tr><td>High risk</td><td>Unclear</td><td>Low</td></tr>
<tr><td>High risk in case of the following:
• Volunteers: Authors only included people who volunteered to participate.
• Example: A large-scale study that requires voluntary participation instead of a random selection of participants.
• Sampling-based on symptoms.
• Other: Please explain further.</td><td>• No information about sampling strategy.</td><td>• All eligible participants were included from a random sample.
• Contact tracing studies that included all contacts from index cases positive for SARS-CoV-2.
• All potential people who could be positive for SARS-CoV-2 were included in a screening study.
Examples:
• A study where all people had to be tested after traveling. Those who were positive were quarantined in a medical facility.
• All people who visited a facility where an outbreak occurred.
• All Residents in a nursing facility</td></tr>
<tr><td colspan="3"><u>Reporting:</u> Was the response rate from the eligible population provided?
Yes
No
Sometimes the authors report the participation or response rate. However, be very careful about the numbers provided by the authors</td></tr>
</table>

Example

- Eligible population: All passengers who were screened before taking a flight in the United States.
- Eligible Participants: All people who got tested for SARS-CoV-2 independently of the result.

If not, can you calculate the response rate? You can try to find if the authors report the number of people who met the inclusion criteria and/ or were invited and how many were finally included in the study (positive and negative for SARS-CoV-2).

$$\text{Response Rate} = \frac{Number\ of\ individuals\ who\ participated\ in\ the\ study}{Number\ of\ elibigle\ participants}\ X\ 100$$

Example
Example

- *Eligible participants:* All passengers before taking a flight in the United States.

- *Individuals who participated in the study:* All passengers who got tested for SARS-CoV-2 independently of the result.

(Number of people who participated in the study/number of eligible participants)
Yes
No
Response Rate:

Num and denominator%

Question 2 - The characteristics of non-respondents, if any, who were eligible are similar to those who participated in the study?

Response rate bias occurs when people who do not take part in a study differ systematically from those who take part in ways that are associated with the condition of interest, even if those invited come from a random selection.

We will not provide an exact cut-point to say there is a risk of response rate bias. We want to assess if there are differences among those who participated and those who did not participate in the study

High	Unclear	Low
• Respondents and non-respondents are different, and the participation might be related to being symptomatic or asymptomatic.<br><br>• Other: Please explain further.	• They might be similar, but no information was provided.	• The characteristics between respondents and non-respondents are similar.<br><br>• There were no non-respondents, the response rate is 100%

Information Bias

Reporting: How was symptomatic and asymptomatic defined?

Based on the reporting question
Question 3 - Was the assessment of symptoms status adequate? In this question, we would like to find the specific type of symptoms assessed in each study (for the type of symptoms commonly present in patients with COVID-19, see link). Please be aware of the appendixes in the supplementary material. In many cases, the list of symptoms is available in this section.

High	Unclear	Low
• The list of assessed symptoms is limited; for example, the study only presents information about flu-like symptoms.<br><br>• Other: Please explain further.	• There is no clear information about symptoms; the authors mention only symptomatic and asymptomatic without further description.	The authors assessed the symptom status based on a well-defined list of symptoms.<br><br>See link WHO-list of symptoms

https://www.who.int/emergencies/diseases/novel-coronavirus-2019/question-and-answers-hub/q-a-detail/coronavirus-disease-covid-19

<table>
<tr><td colspan="3">Question 4- Based on the method symptoms were collected, is there a risk of recall bias?
Recall bias: Occurs when the condition has been measured through surveys or questionnaires that rely on memory to provide information on the condition of interest.

We are aware that symptoms are a subjective report, but we rely on the participant’s memory to answer our research question. However, we want to assess if researchers asked participants or located the information about symptoms with enough frequency to minimize recall bias. Please see the options for high, low, or unclear.</td></tr>
<tr><td>High</td><td>Unclear</td><td>Low</td></tr>
<tr><td>Symptom status was assessed with a delay of one or two weeks.

• Example: Participants were asked, ‘Have you had any symptoms or the following symptoms during the past two weeks?’</td><td>• There is no clear information about the method for collecting symptoms.</td><td>• Symptom status was collected frequently during the follow-up. (i.e., daily, two times per week)

• Symptom status was assessed retrospectively in clinical charts, and the researchers stated that symptom status was frequently evaluated (i.e., daily, two times per week).</td></tr>
<tr><td colspan="3">INFORMATION BIAS - Misclassification</td></tr>
<tr><td colspan="3">Reporting: In the study, how did the authors report the follow-up of symptom status? You can select or more options
✓ 14 days after exposure
✓ Until one or more negative PCR
✓ Seven days after testing and 14 days after exposure
✓ More than one of the mentioned above
✓ Longer follow-up</td></tr>
<tr><td colspan="3">Was the viral load reported?
Yes
No

If yes, please provide the information about it CT. Add definition
*CT: Cycle threshold</td></tr>
</table>

Question 5- Is there a risk that the asymptomatic status was misclassified because of the follow-up? This question is not about the type of symptoms. It is about the follow-up period of symptoms among those who were positive for SARS-CoV-2 taking into account the course of the infection.		
**High**	**Unclear**	**Low**
• Follow-up 7 days after testing<br>• Other: Please explain further.	• Follow-up 14 days after the last possible exposure	• Until negative PCR<br>• 14 after exposure and 7 days after testing<br>• A Longer follow-up
**Selective or incomplete reporting of outcome (asymptomatic status)**		
Reporting<br>The authors provide information about the symptom status of all positive cases<br>Yes<br>No		
Question 6- Based on your previous answer<br>Is there a risk of incomplete or selective reporting of symptoms status among those positive for SARS-CoV-2?		
**High**	**Unclear**	**Low**
• There is missing information about the final symptom status of a proportion of participants.	• The numbers are not clearly reported	• All participants were followed up for symptom status and were included in the analysis.
Based on the risk of bias assessment, please provide some thoughts about the overall assessment of the study. Is there a risk of over or under-estimating the proportion of the truly asymptomatic population?<br><br>Please select one of the following and write down your comments about it		
