# Supplementary material for: Occurrence and transmission potential of asymptomatic and presymptomatic SARS-CoV-2 infections: Update of a living systematic review and meta-analysis
Source: PLoS Med. 2022 May 26;19(5):e1003987. doi: 10.1371/journal.pmed.1003987 (PMC9135333; doi:10.1371/journal.pmed.1003987)
Supplement: S1 Appendix — (PDF) [file pmed.1003987.s004.pdf]

S1 Appendix. Data extraction forms

Screening Form

Record ID:

Screening assigned to [screen1 assigned]; performed by:

(change only if assignment has changed)

Verification assigned to [screen1 veri]; performed by:

(change only if assignment has changed)

Resolution assigned to [screen1 resolve]; performed by:

(change only if assignment has changed)

record no [references redcap database]

preprint refnr [references redcap database]

PDF of article

Citation meta data

Authors

First author

(automatically imported; Lastname, IN)

Publication year

Title

Abstract

Author 1

Journal

issn

volume

issue	
pages	
DOI	
URL	
Source	◯ MedRxiv<br>◯ BioRxiv<br>◯ EMBASE<br>◯ PubMed<br>◯ Other

Screening

Comments	
Help	◯ Yes<br>◯ No
Reference number in publication	
To be included in the review	◯ Yes ◯ No
Reason for exclusion	◯ Excluded based on title/abstract<br>◯ Publication not on COVID-19<br>◯ No original data<br>◯ Data included in other publication (i.e. same population reported twice)<br>◯ Duplicate (i.e. exact same article)<br>◯ Insufficient extractable data<br>◯ Study design inappropriate<br>◯ Infections not diagnosed with a molecular test OR serologic test<br>◯ Aim of mathematical model not in review scope<br>◯ Inadequate or undocumented follow-up time<br>◯ Preprint of published article<br>◯ Other
Serological study	◯ Yes<br>◯ No
Comment from screening	
Major study design group from the screening	◯ EPI<br>◯ BASIC<br>◯ Non-Original<br>◯ NA

Major study design group	◯ EPI<br>◯ BASIC<br>◯ Non-Original<br>◯ NA
Verification	
Verification comment	
Resolution comment	

Data extraction form

General Information

Record ID:

author: [author_1] ([year]) [title]

General information

Aim/objective of the study:

(As reported by author)

Study design:

◯ Case series
◯ Cross sectional study
◯ Cohort study
◯ Mathematical modelling study
◯ Case-control study
◯ Outbreak Investigation
◯ Other

Please specify "other" study design:

SARS-CoV-2 infection diagnosed by:

◯ PCR
◯ Serology
◯ Both PCR and serology

Setting of data collection:

◯ Cruise ship
◯ Hospital
◯ Traveler/evacuee from an affected area
◯ Contact tracing
◯ Health care worker
◯ Family cluster
◯ Other

Please specify "other" setting:

Setting 2 [used in Q1, Q2 analysis]

◯ 1. Contact investigation
◯ 2. Contact investigation, aggregated
◯ 3. Outbreak investigation
◯ 4. Statistical model
◯ 5. Screening
◯ 6. Hospitalised adults
◯ 7. Hospitalised children
◯ 8. Hospitalised children and adults
◯ 9. Screening: institutional setting
◯ 10. Screening: community setting
◯ 11. Screening: occupational

Country:

◯ Afghanistan
◯ Albania
◯ Algeria
◯ Andorra
◯ Angola
◯ Antigua and Barbuda
◯ Argentina
◯ Armenia
◯ Aruba
◯ Australia
◯ Austria
◯ Azawad
◯ Azerbaijan
◯ Bahamas
◯ Bahrain
◯ Bangladesh
◯ Barbados
◯ Belarus
◯ Belgium
◯ Belize
◯ Benin
◯ Bermuda
◯ Bhutan
◯ Bolivia
◯ Bosnia and Herzegovina
◯ Botswana
◯ Brazil
◯ Brunei
◯ Bulgaria
◯ Burkina Faso
◯ Burundi
◯ Cambodia
◯ Cameroon
◯ Canada
◯ Cape Verde
◯ Central African Republic
◯ Chad
◯ Chechnya
◯ Chile
◯ China
◯ Colombia
◯ Comoros
◯ Congo
◯ Costa Rica
◯ Côte d'Ivoire
◯ Croatia
◯ Cuba
◯ Curaçao
◯ Cyprus
◯ Czechoslovakia,
◯ Denmark
◯ Dominican Republic
◯ Ecuador
◯ Egypt
◯ El Salvador
◯ Equatorial Guinea
◯ Eritrea
◯ Estonia
◯ Ethiopia
◯ Fiji
◯ Finland
◯ France
◯ French Guyana
◯ Gabon
◯ Gambia
◯ Georgia
◯ Germany
◯ Ghana
◯ Greece

◯ Greenland
◯ Guatemala
◯ Guinea
◯ Guyana
◯ Haiti
◯ Honduras
◯ Hong Kong
◯ Hungary
◯ Iceland
◯ India
◯ Indonesia
◯ Iran
◯ Iraq
◯ Ireland
◯ Israel
◯ Italy
◯ Jamaica
◯ Japan
◯ Jordan
◯ Kazakhstan
◯ Kenya
◯ Kosovo
◯ Kuwait
◯ Kyrgyzstan
◯ Laos
◯ Latvia
◯ Lebanon
◯ Liberia
◯ Libya
◯ Liechtenstein
◯ Lithuania
◯ Luxembourg
◯ Macedonia
◯ Madagascar
◯ Malawi
◯ Malaysia
◯ Maldives
◯ Mali
◯ Malta
◯ Mauritius
◯ Mexico
◯ Moldova
◯ Monaco
◯ Mongolia
◯ Montenegro
◯ Morocco
◯ Mozambique
◯ Myanmar
◯ Multiple
◯ Namibia
◯ Nepal
◯ Netherlands
◯ New Zealand
◯ Nicaragua
◯ Niger
◯ Nigeria
◯ North Korea
◯ North Yemen
◯ Norway
◯ Oman
◯ Pakistan
◯ Palestine
◯ Panama
◯ Papua New Guinea
◯ Paraguay
◯ Peru
◯ Philippines
◯ Poland
◯ Portugal
◯ Puerto Rico
◯ Qatar

◯ Romania
◯ Russia
◯ Rwanda
◯ Samoa
◯ San Marino
◯ Saudi Arabia
◯ Senegal
◯ Serbia
◯ Seychelles
◯ Sierra Leone
◯ Singapore
◯ Slovakia
◯ Slovenia
◯ Solomon Islands
◯ Somalia
◯ South Africa
◯ South Korea
◯ South Sudan
◯ South Yemen
◯ Spain
◯ Sri Lanka
◯ Sudan
◯ Suriname
◯ Swaziland
◯ Sweden
◯ Switzerland
◯ Syria
◯ Tajikistan
◯ Tanzania
◯ Thailand
◯ Tibet
◯ Togo
◯ Tunisia
◯ Turkey
◯ Turkmenistan
◯ Uganda
◯ Ukraine
◯ United Arab Emirates
◯ United Arab Republic
◯ United Kingdom
◯ United States of America
◯ Uruguay
◯ Uzbekistan
◯ Venezuela
◯ Vietnam
◯ Yemen
◯ Yugoslavia
◯ Zambia
◯ Zanzibar
◯ Zimbabwe
◯ Sao Tome and Principe
◯ More than one country
◯ Other

Region or comments from "country":

Duration of follow-up time (choose all that apply):

☐ 14 days after the last possible exposure date
☐ 7 days after diagnosis
☐ Until negative PCR result
☐ >7 days after diagnosis

Which questions are addressed in this study?	☐ Question 1: Amongst people who become infected with SARS-CoV-2, what proportion does not experience symptoms at all during their infection?<br>☐ DO NOT CHOOSE - Question 2: Amongst people who become infected with SARS-CoV-2, what proportion has no symptoms at the time of testing, but develops symptoms later?<br>☐ Question 2.1: What is the secondary attack rate (SAR) from asymptomatic or pre-symptomatic index cases?ORQuestion 2.2: What proportion of SARS-CoV-2 infections is accounted for by people who are either asymptomatic throughout infection, or pre-symptomatic?
Comments:	(Enter "none" if no comments)
Extracted by:	(Last name only)
Verified by:	(Last name only)

Question 1

Record ID:

author: [author_1] ([year]) [title]

Question 1: Amongst people who become infected with SARS-CoV-2, what proportion does not experience symptoms at all during their infection?

Reminder: Question 1: Proportion or rate of people with asymptomatic SARS-CoV-2 infections who do not experience symptoms at all during their infection (i.e. from the time of testing to the end of the follow-up);

Number of clusters described:	◯ 1<br>◯ 2<br>◯ 3<br>◯ 4<br>◯ NA
Omit from meta-analysis	◯ Yes<br>◯ No
Number of individuals that were asymptomatic throughout infection:	
Total number of individuals (denominator):	
Number of individuals that were asymptomatic throughout infection [cluster2]:	
Total number of individuals (denominator) [cluster2]:	
Number of individuals that were asymptomatic throughout infection [cluster3]:	
Total number of individuals (denominator) [cluster3]:	

Follow-up time

Please describe the follow-up time in days. The description should include how follow-up times were measured and for which people follow-up times were reported.

Sex

Number of females who remained asymptomatic throughout infection:

(Enter "9999" if not reported.)

Number of males who remained asymptomatic throughout infection:

(Enter "9999" if not reported.)

Age

Enter median age of all people who remained asymptomatic throughout infection:

(Enter "9999" if not reported.)

Enter IQR for age of all people who remained asymptomatic throughout infection:

(Enter "9999" if not reported.)

Age range of asymptomatic

Select one or more if applicable

- ☐ Children (< 18 years)
- ☐ Adults (18 - 65 years)
- ☐ Older adults (>65 years)
- ☐ All ages
- ☐ Not Reported

Comments

Comments:

Extracted by:

(Enter last name)

Verified by:

(Enter last name)

Question 2.1 and 2.2

Record ID:

author: [author_1] ([year]) [title]

What does this study report?

- ◯ Q. 2.1 SAR
- ◯ Q. 2.2 Transmission proportion
- ◯ Both
- ◯ NA

Summary of results:

Question 2.1
What is the secondary attack rate from asymptomatic or pre-symptomatic index cases?

SAR (Secondary attack rate)
Number of infections (numerator) caused by certain group of people: Here asymptomatics vs symptomatics (denominator)

Asymptomatic SAR

(number infected;number asymptomatic)

Asymptomatic SAR Risk Ratio (RR)

(Enter "9999" if not reported.)

Asymptomatic SAR Risk Ratio (RR) Lower Confidence Interval

(Enter "9999" if not reported.)

Asymptomatic SAR Risk Ratio (RR) Upper Confidence Interval

(Enter "9999" if not reported.)

Asymptomatic SAR Odds Ratio (OR)

(Enter "9999" if not reported.)

Asymptomatic SAR Odds Ratio (OR) Lower Confidence Interval

(Enter "9999" if not reported.)

Asymptomatic SAR Odds Ratio (OR) Upper Confidence Interval

(Enter "9999" if not reported.)

Pre-symptomatic SAR

(number infected;number pre-symptomatic)

Pre-symptomatic SAR Risk Ratio (RR)	(Enter "9999" if not reported.)
Pre-symptomatic SAR Risk Ratio (RR) Lower Confidence Interval	(Enter "9999" if not reported.)
Pre-symptomatic SAR Risk Ratio (RR) Upper Confidence Interval	(Enter "9999" if not reported.)
Pre-symptomatic SAR Odds Ratio (OR)	(Enter "9999" if not reported.)
Pre-symptomatic SAR Odds Ratio (RR) Lower Confidence Interval	(Enter "9999" if not reported.)
Pre-symptomatic SAR Odds Ratio (OR) Upper Confidence Interval	(Enter "9999" if not reported.)
Mild symptomatic SAR	(number infected;number mild symptomatic)
Moderate symptomatic SAR	(number infected;number mild symptomatic)
Severe/critical symptomatic SAR	(number infected;number critical symptomatic)
ANY symptomatic SAR	(number infected;number ANY symptomatic)

Question 2.2: What proportion of SARS-CoV-2 infections is accounted for by people who are either asymptomatic throughout infection, or pre-symptomatic?

Region/setting	(If multiple settings are described, please specify and separate with ';')

Asymptomatic transmission

Proportion asymptomatic transmission (median)

(For all fields: if mutiple settings are described use ';' to separate extraction)

Proportion asymptomatic transmission (lower CrI)

Proportion asymptomatic transmission (upper CrI)

Pre-symptomatic transmission

Proportion pre-symptomatic transmission (median)

(For all fields: if mutiple settings are described use ';' to separate extraction)

Proportion pre-symptomatic transmission (lower CrI)

Proportion pre-symptomatic transmission (upper CrI)

Comment:

Extraction

Extracted by:

(Enter last name)

Verfied by:

(Enter last name)

Risk of Bias - observational EPI [update 3]

Record ID:

ROB assigned to [rob reviewer 1]; performed by:

((e.g., NL))

ROB assigned to [rob reviewer 2]; performed by:

((e.g., NL))

Resolution assigned to [rob reviewer 3]; performed by:

(R1) If the study reports more than one cluster, please describe the participants of cluster 1 and cluster 2:

(R2) If the study reports more than one cluster, please describe the participants of cluster 1 and cluster 2:

Selection Bias

(R1) Reporting: How was the target population described?

((Extract it from the study))

(R1) Reporting: How was the target population described in cluster 2?

((Extract it from the study))

(R2) Reporting: How was the target population described?

((Extract it from the study))

(R2) Reporting: How was the target population described in cluster 2?

((Extract it from the study))

(R1) Question 1- Was the sample invited to participate a close or true representation of the target population?

◯ High ◯ Unclear ◯ Low

(R1) Question 1- Was the sample invited in cluster 2 to participate a close or true representation of the target population?

◯ High ◯ Unclear ◯ Low ◯ NA

(R1) Comments

(R2) Question 1- Was the sample invited to participate a close or true representation of the target population?	◯ High ◯ Unclear ◯ Low
(R2) Question 1- Was the sample invited in cluster 2 to participate a close or true representation of the target population?	◯ High ◯ Unclear ◯ Low ◯ NA
(R2) Comments	
(Consensus) Question 1- Was the sample invited to participate a close or true representation of the target population?	◯ High ◯ Unclear ◯ Low
(Consensus) Question 1- Was the sample invited in cluster 2 to participate a close or true representation of the target population?	◯ High ◯ Unclear ◯ Low

Response Rate

(R1) Reporting: Was the response rate from the eligible population provided?	◯ Yes ◯ No
(R1) Reporting: Was the response rate from the eligible population in cluster 2 provided?	◯ Yes ◯ No ◯ NA
(R2) Reporting: Was the response rate from the eligible population provided?	◯ Yes ◯ No
(R2) Reporting: Was the response rate from the eligible population in cluster 2 provided?	◯ Yes<br>◯ No<br>◯ NA
(R1) If the response rate is not provided. Can you calculate the response rate?	◯ Yes ◯ No ◯ NA
(R1) If the response rate for cluster 2 is not provided. Can you calculate the response rate for cluster 2?	◯ Yes ◯ No ◯ NA
(R2) If the response rate is not provided. Can you calculate the response rate?	◯ Yes ◯ No ◯ NA
(R2) If the response rate for cluster 2 is not provided. Can you calculate the response rate for cluster 2?	◯ Yes<br>◯ No<br>◯ NA
(R1) Number of individuals who participated in the study	
(R1) Number of individuals in cluster 2 who participated in the study	
(R2) Number of individuals who participated in the study	

(R2) Number of individuals in cluster 2 who participated in the study

(R1) Number of eligible participants

(R1) Number of eligible participants for cluster 2

(R2) Number of eligible participants

(R2) Number of eligible participants for cluster 2

(R1) Response rate (%)

(R1) Response rate (%) for cluster 2

(R2) Response rate (%)

(R2) Response rate (%) for cluster 2

(R1) Comments

(R2) Comments

(Consensus) Response rate (%)

(Consensus) Response rate (%) for cluster 2

Selection Bias - Response Rate

(R1) Question 2- The characteristics of non-respondents, if any, who were eligible are similar to those who participated in the study?

○ High ○ Unclear ○ Low

(R1) Question 2- The characteristics of non-respondents for cluster 2, if any, who were eligible are similar to those who participated in the study?

○ High
○ Unclear
○ Low
○ NA

(R1) Comments

(R2) Question 2- The characteristics of non-respondents, if any, who were eligible are similar to those who participated in the study?	◯ High ◯ Unclear ◯ Low
(R2) Question 2- The characteristics of non-respondents, if any, who were eligible are similar to those who participated in the study for cluster 2?	◯ High<br>◯ Unclear<br>◯ Low<br>◯ NA
(R2) Comments	
(Consensus) Q2- The characteristics of non-respondents, if any, who were eligible are similar to those who participated in the study?	◯ High ◯ Unclear ◯ Low
(Consensus) Q2- The characteristics of non-respondents, if any, who were eligible are similar to those who participated in the study for cluster 2?	◯ High ◯ Unclear ◯ Low

Information Bias

(R1) Reporting: How was symptomatic and asymptomatic defined?	
(R2) Reporting: How was symptomatic and asymptomatic defined?	
(R1) Question 3 - Was the assessment of symptoms status adequate?	◯ High ◯ Unclear ◯ Low
(R1) Question 3 - Was the assessment of symptoms status adequate for cluster 2?	◯ High<br>◯ Unclear<br>◯ Low<br>◯ NA
(R1) Comments	
(R2) Question 3 - Was the assessment of symptoms status adequate ?	◯ High ◯ Unclear ◯ Low
(R2) Question 3 - Was the assessment of symptoms status adequate for cluster 2?	◯ High<br>◯ Unclear<br>◯ Low<br>◯ NA
(R2) Comments	
(Consensus) Q3- Was the assessment of symptoms status adequate?	◯ High ◯ Unclear ◯ Low
(Consensus) Q3- Was the assessment of symptoms status adequate for cluster 2?	◯ High ◯ Unclear ◯ Low

(R1) Question 4- Based on the method symptoms were collected, is there a risk of recall bias?	◯ High ◯ Unclear ◯ Low
(R1) Question 4- Based on the method symptoms were collected, is there a risk of recall bias for cluster 2?	◯ High ◯ Unclear ◯ Low ◯ NA
(R1) Comments	
(R2) Question 4- Based on the method symptoms were collected, is there a risk of recall bias?	◯ High ◯ Unclear ◯ Low
(R2) Question 4- Based on the method symptoms were collected, is there a risk of recall bias for cluster 2?	◯ High ◯ Unclear ◯ Low ◯ NA
(R2) Comments	
(Consensus) Q4- Based on the method symptoms were collected, is there a risk of recall bias?	◯ High ◯ Unclear ◯ Low
(Consensus) Q4- Based on the method symptoms were collected, is there a risk of recall bias for cluster 2?	◯ High ◯ Unclear ◯ Low
(R1) Reporting: For how long were the participants followed to assess symptom status?	☐ 14 days after the last possible exposure date ☐ 7 days after diagnosis<br>☐ Until negative PCR result<br>☐ Both 14 days after the last possible exposure date AND 7 days after diagnosis<br>☐ Longer follow-up
(R1) Reporting: For how long were the participants followed to assess symptom status in cluster 2?	☐ 14 days after the last possible exposure date<br>☐ 7 days after diagnosis<br>☐ Until negative PCR result<br>☐ Both 14 days after the last possible exposure date AND 7 days after diagnosis<br>☐ Longer follow-up<br>☐ NA
(R2) Reporting: For how long were the participants followed to assess symptom status?	☐ 14 days after the last possible exposure date ☐ 7 days after diagnosis<br>☐ Until negative PCR result<br>☐ Both 14 days after the last possible exposure date AND 7 days after diagnosis<br>☐ Longer follow-up
(R2) Reporting: For how long were the participants followed to assess symptom status?	☐ 14 days after the last possible exposure date<br>☐ 7 days after diagnosis<br>☐ Until negative PCR result<br>☐ Both 14 days after the last possible exposure date AND 7 days after diagnosis<br>☐ Longer follow-up<br>☐ NA

(Consensus) Reporting: For how long were the participants followed to assess symptom status?	☐ 14 days after the last possible exposure date ☐ 7 days after diagnosis<br>☐ Until negative PCR result<br>☐ Both 14 days after the last possible exposure date AND 7 days after diagnosis<br>☐ Longer follow-up
(Consensus) Reporting: For how long were the participants followed to assess symptom status?	◯ High ◯ Unclear ◯ Low
(R1) Reporting: Was the viral load reported? Please provide information about it CT (cycle threshold) values.	(If not reported, please write 'NR')
(R2) Reporting: Was the viral load reported? Please provide information about it CT (cycle threshold) values.	(If not reported, please write 'NR')
(R1) Question 5- Is there a risk that asymptomatic status was misclassified?	◯ High ◯ Unclear ◯ Low
(R1) Question 5- Is there a risk that asymptomatic status was misclassified in cluster 2?	◯ High ◯ Unclear ◯ Low<br>◯ NA
(R1) Comments	
(R2) Question 5- Is there a risk that asymptomatic status was misclassified?	◯ High ◯ Unclear ◯ Low
(R2) Question 5- Is there a risk that asymptomatic status was misclassified in cluster 2?	◯ High ◯ Unclear ◯ Low<br>◯ NA
(R2) Comments	
(Consensus) Q5- Is there a risk that asymptomatic status was misclassified?	◯ High ◯ Unclear ◯ Low
(Consensus) Q5- Is there a risk that asymptomatic status was misclassified in cluster 2?	◯ High ◯ Unclear ◯ Low

Selective or Incomplete Reporting of Outcome

(R1) Reporting: The authors present the symptom status of all participants at the end of follow-up?	◯ Yes ◯ No
(R1) Reporting: The authors present the symptom status of all participants in cluster 2 at the end of follow-up?	◯ Yes ◯ No ◯ NA
(R2) Reporting: The authors present the symptom status of all participants at the end of follow-up?	◯ Yes ◯ No

(R2) Reporting: The authors present the symptom status of all participants in cluster 2 at the end of follow-up?	◯ Yes ◯ No ◯ NA
(R1) Question 6-Is there a risk of incomplete or selective reporting of symptoms status among those who were positive for SARS-CoV-2?	◯ High ◯ Unclear ◯ Low
(R1) Question 6-Is there a risk of incomplete or selective reporting of symptoms status among those who were positive for SARS-CoV-2 in cluster 2?	◯ High ◯ Unclear ◯ Low ◯ NA
(R1) Comments	
(R2) Question 6-Is there a risk of incomplete or selective reporting of symptoms status among those who were positive for SARS-CoV-2?	◯ High ◯ Unclear ◯ Low
(R2) Question 6-Is there a risk of incomplete or selective reporting of symptoms status among those who were positive for SARS-CoV-2 in cluster 2?	◯ High ◯ Unclear ◯ Low ◯ NA
(R2) Comments	
(Consensus) Q6- Is there a risk of incomplete or selective reporting of symptoms status?	◯ High ◯ Unclear ◯ Low
(Consensus) Q6- Is there a risk of incomplete or selective reporting of symptoms status in cluster 2?	◯ High ◯ Unclear ◯ Low

Final Comments

(R1) Is there a risk of over or under-estimating the proportion of the truly asymptomatic population?	◯ Underestimation of the proportion of asymptomatics ◯ Overestimation of the proportion of asymptomatics ◯ The proportion provided by the authors is accurate ◯ Unclear
(R1) Is there a risk of over or under-estimating the proportion of the truly asymptomatic population in cluster 2?	◯ Underestimation of the proportion of asymptomatics ◯ Overestimation of the proportion of asymptomatics ◯ The proportion provided by the authors is accurate ◯ Unclear ◯ NA
(R1) Comments	
(R2) Is there a risk of over or under-estimating the proportion of the truly asymptomatic population?	◯ Underestimation of the proportion of asymptomatics ◯ Overestimation of the proportion of asymptomatics ◯ The proportion provided by the authors is accurate ◯ Unclear

(R2) Is there a risk of over or under-estimating the proportion of the truly asymptomatic population in cluster 2?	◯ Underestimation of the proportion of asymptomatics ◯ Overestimation of the proportion of asymptomatics ◯ The proportion provided by the authors is accurate ◯ Unclear ◯ NA
(R2) Comments	
Reviewer 3 (Consensus) - comments	
