# Supplementary material for: Occurrence and transmission potential of asymptomatic and presymptomatic SARS-CoV-2 infections: Update of a living systematic review and meta-analysis
Source: PLoS Med. 2022 May 26;19(5):e1003987. doi: 10.1371/journal.pmed.1003987 (PMC9135333; doi:10.1371/journal.pmed.1003987)
Supplement: S2 Appendix — (PDF) [file pmed.1003987.s005.pdf]

S2 Appendix. Analysis of other systematic reviews of asymptomatic SARS-CoV-2 infection

First author, publication year [ref]	Studies included, n	Search dates	Inclusion criteria[a]	Summary estimate[b] (95% CI)	I^2	τ^2	Prediction interval
Byambasuren 2020 [1]	13	Until 20 July 2020	"…primary studies on asymptomatic prevalence in which (1) the sample frame includes at-risk populations and (2) follow-up was sufficient to identify pre-symptomatic cases"	18% (11% - 27%)	81%	0.68	3% - 59%
Beale 2020 [2]	21	Until 25 August 2020	"…studies based in community settings that involved systematic PCR testing on participants and follow-up symptom monitoring regardless of symptom status."	21% (13% - 33%)	86%	1.59	2% - 80%
Chen 2021 [3]	241	Until 31 December 2020	"Original investigations with sample size (or number of subjects) not less than five were included for further analyses."	20% (17% - 23%)	99%	2.35	1% - 83%
Ma 2021 [4]	95[c]	Until 4 February 2021	"Cross-sectional studies, cohort studies, case series studies, and case series on transmission reporting the number of asymptomatic infections among the tested and confirmed COVID-19 populations that were published in Chinese or English were included."	51% (42% - 60%)	97%	2.80	4% - 97%
	77[d]			38% (32% - 44%)	97%	1.26	6% - 85%
Sah 2021 [5]	170	Until 2 April 2021	"Studies that reported silent infections at the time of testing, whether presymptomatic or asymptomatic. Index cases were removed to minimize representational bias that would result in overestimation of symptomaticity."	36% (31% - 41%)	94%	1.90	3% - 89%

[a] Text extracted from study abstract;

[b] Using the random effects model and the logit transformation to calculate a summary estimate in the *metaprop* package in R. All studies published summary estimates, 95% CI, and I^2 values using their own methodology. Only one study (Beale) published a τ^2 value. None of the studies published a prediction interval;

[c] Including all studies (stratum 1 in the Figure);

[d] Excluding studies in which all cases were asymptomatic (stratum 2 in the Figure).

Figure. Graphical representation of summary estimates of asymptomatic SARS-CoV-2 infection extracted from other systematic reviews

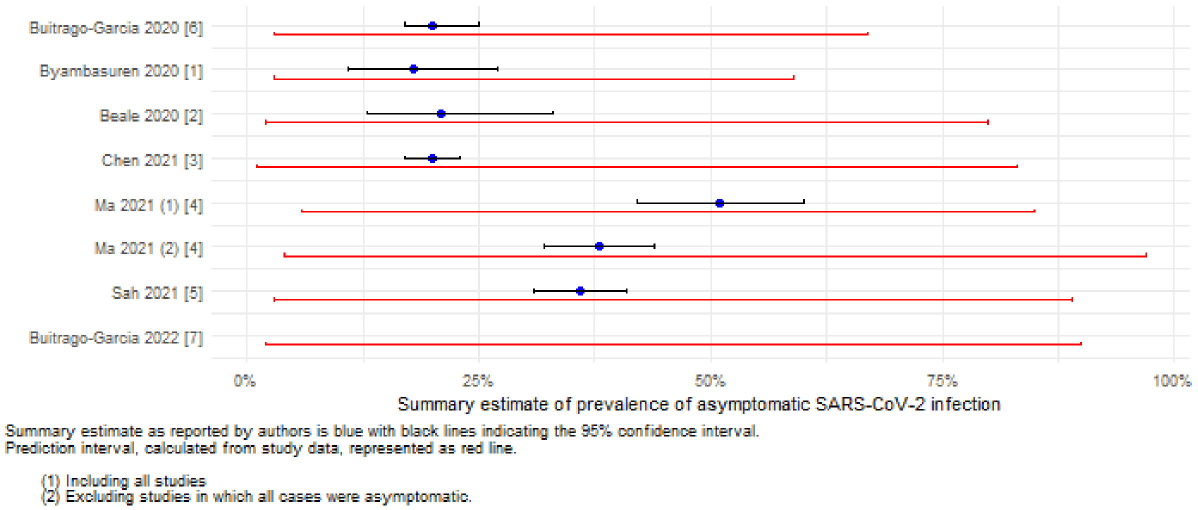

References

1. Byambasuren O, Cardona M, Bell K, Clark J, McLaws M-L, Glasziou P. Estimating the Extent of Asymptomatic Covid-19 and Its Potential for Community Transmission: Systematic Review and Meta-Analysis. Official Journal of the Association of Medical Microbiology and Infectious Disease Canada. 2020;5(4):223-34. https://doi.org/10.3138/jammi-2020-0030.

2. Beale S, Hayward A, Shallcross L, Aldridge R, Fragaszy E. A Rapid Review and Meta-Analysis of the Asymptomatic Proportion of Pcr-Confirmed Sars-Cov-2 Infections in Community Settings [Version 1; Peer Review: 1 Approved with Reservations]. Wellcome Open Research. 2020;5(266). https://doi.org/10.12688/wellcomeopenres.16387.1.

3. Chen X, Huang Z, Wang J, Zhao S, Wong MC-S, Chong KC, et al. Ratio of Asymptomatic Covid-19 Cases among Ascertained Sars-Cov-2 Infections in Different Regions and Population Groups in 2020: A Systematic Review and Meta-Analysis Including 130 123 Infections from 241 Studies. BMJ Open. 2021;11(12):e049752. http://dx.doi.org/10.1136/bmjopen-2021-049752.

4. Ma Q, Liu J, Liu Q, Kang L, Liu R, Jing W, et al. Global Percentage of Asymptomatic Sars-Cov-2 Infections among the Tested Population and Individuals with Confirmed Covid-19 Diagnosis: A Systematic Review and Meta-Analysis. JAMA Network Open. 2021;4(12):e2137257-e. https://doi.org/10.1001/jamanetworkopen.2021.37257.

5. Sah P, Fitzpatrick MC, Zimmer CF, Abdollahi E, Juden-Kelly L, Moghadas SM, et al. Asymptomatic Sars-Cov-2 Infection: A Systematic Review and Meta-Analysis. Proc Natl Acad Sci U S A. 2021;118(34). Epub 2021/08/12. https://doi.org/10.1073/pnas.2109229118. PubMed PMID: 34376550; PubMed Central PMCID: 8403749.

6. Buitrago-Garcia D, Egli-Gany D, Counotte MJ, Hossmann S, Imeri H, Ipekci AM, et al. Occurrence and transmission potential of asymptomatic and presymptomatic SARS-CoV-2 infections: A living systematic review and meta-analysis. PLOS Med 2020;17(9): e1003346. https://doi.org/10.1371/journal.pmed.1003346. PubMed PMID: 32960881.

7. Buitrago-Garcia D, Ipekci AM, Heron L, Imeri H, Araujo-Chaveron L, Arevalo-Rodriguez I, et al. Occurrence and transmission potential of asymptomatic and presymptomatic SARS-CoV-2 infections: update of a living systematic review and meta-analysis. PLOS Med 2022 https://doi.org/10.1371/journal.pmed.1003987.
