# Supplementary material for: Occurrence and transmission potential of asymptomatic and presymptomatic SARS-CoV-2 infections: Update of a living systematic review and meta-analysis
Source: PLoS Med. 2022 May 26;19(5):e1003987. doi: 10.1371/journal.pmed.1003987 (PMC9135333; doi:10.1371/journal.pmed.1003987)
Supplement: S1 Table — (PDF) [file pmed.1003987.s006.pdf]

S1 Table. Studies included in version 3 and excluded in versions 4 and 5 of the living systematic review

Author	Reason for Exclusion
**Contact investigations**	
Tong ZD [1]	Case series of already diagnosed cases
Huang R [2]	Contact investigation of a single family or individual
Jiang XL [3]	Contact investigation of a single family or individual
Jiang X [4]	Contact investigation of a single family or individual
Liao J [5]	The study only included diagnosed cases
Hu Z [6]	The study only included diagnosed cases
Luo SH [7]	Contact investigation of a single family or individual
Chan JF [8]	Contact investigation of a single family or individual
Ye F [9]	Contact investigation of a single family or individual
Bai Y [10]	Contact investigation of a single family or individual
Luo Y [11]	Contact investigation of a single family or individual
Zhang J [12]	Contact investigation of a single family or individual
Zhang B [13]	Contact investigation of a single family or individual
Huang L [14]	Contact investigation of a single family or individual
Qian G [15]	Contact investigation of a single family or individual
Gao Y [16]	Case series that do not enrol consecutive patients
**Contact investigations aggregated**	
Wang Z [17]	Not all contacts were tested for SARS-CoV-2
Yang R [18]	The study only included diagnosed cases
Bi Q [19]	Asymptomatic status only ascertained at start
**Outbreak investigations**	
Roxby AC [20]	Inadequate follow-up unclear information about the last possible exposure.
Solbach W [21]	The study only included diagnosed cases
Mizumoto K [22]	Data included in another publication
Tian S [23]	Preprint of published article

Pham TQ [24]	Preprint of published article
**Screening**	
Arima Y [25]	Same population reported twice
Lytras T [26]	Only included pre-symptomatic patients
Lombardi A [27]	Preprint of published article
**Hospitalised adults**	
Pongpirul WA [28]	The study only included diagnosed cases
Qiu C [29]	The study only included diagnosed cases
Zou L [30]	The study only included diagnosed cases
Zhou R [31]	The study only included diagnosed cases
Chang MC [32]	The study only included diagnosed cases
Zhou X [33]	The study only included diagnosed cases
Angelo Vaira L [34]	The study only included diagnosed cases
Wang X [35]	The study only included diagnosed cases
Xu T [36]	The study only included diagnosed cases
Tabata S [37]	The study only included diagnosed cases
Noh JY [38]	The study only included diagnosed cases
Kumar R [39]	The study only included diagnosed cases
Meng H [40]	Only included pre-symptomatic patients
Zhang Z [41]	Only included pre-symptomatic patients
Al-Shamsi HO [42]	Only included pre-symptomatic patients
Wang Y1 [43]	Only included pre-symptomatic patients
**Hospitalised children**	
See KC [44]	The study only included diagnosed cases
Tan YP [45]	The study only included diagnosed cases
Tan X [46]	The study only included diagnosed cases
Melgosa M [47]	The study only included diagnosed cases
Wu HP [48]	Inadequate follow-up.
Song W [49]	The study only included diagnosed cases

Bai K [50]	The study only included diagnosed cases
Xu H [51]	The study only included diagnosed cases
Qiu H [52]	The study only included diagnosed cases
Lu Y [53]	The study only included diagnosed cases
**Hospitalised adults and children**	
Merza MA [54]	The study only included diagnosed cases
Yongchen Z [55]	The study only included diagnosed cases
Ma Y [56]	The study only included diagnosed cases
Kim SE [57]	The study only included diagnosed cases
Choe PG [58]	The study only included diagnosed cases
Sharma AK [59]	The study only included diagnosed cases
Zhang W3 [60]	The study only included diagnosed cases
Alshami AA [61]	The study only included diagnosed cases
Kong W [62]	The study only included diagnosed cases
Wang Y2 [63]	The study only included diagnosed cases
**Mathematical Models**	
Ganyani T [64]	Data included in another publication
Kim Y [65]	Preprint of published article (first author Chun)
Emery [66]	Preprint of published article
Casey [67]	Mathematical model not in review scope - published version did not include pooled estimate of presymptomatic transmission

References

1. Tong ZD, Tang A, Li KF, Li P, Wang HL, Yi JP, et al. Potential Presymptomatic Transmission of Sars-Cov-2, Zhejiang Province, China, 2020. Emerg Infect Dis. 2020;26(5):1052-4. Epub 2020/02/25. doi: https://doi.org/10.3201/eid2605.200198. PMID: 32091386; PubMed Central PMCID: PMC7181913.

2. Huang R, Zhao H, Wang J, Yan X, Shao H, Wu C. A Family Cluster of Covid-19 Involving an Asymptomatic Case with Persistently Positive Sars-Cov-2 in Anal Swabs. Travel Med Infect Dis. 2020. doi: https://doi.org/10.1016/j.tmaid.2020.101745 PMID: 32425697.

3. Jiang XL, Zhang XL, Zhao XN, Li CB, Lei J, Kou ZQ, et al. Transmission Potential of Asymptomatic and Paucisymptomatic Sars-Cov-2 Infections: A Three-Family Cluster Study in China. J Infect Dis. 2020. doi: 10.1093/infdis/jiaa206. PMID: 32319519.

4. Jiang X, Luo M, Zou Z, Wang X, Chen C, Qiu J. Asymptomatic Sars-Cov-2 Infected Case with Viral Detection Positive in Stool but Negative in Nasopharyngeal Samples Lasts for 42 Days. J Med Virol. 2020. doi: https://doi.org/10.1093/infdis/jiaa206. PMID: 32319519.

5. Liao J, Fan S, Chen J, Wu J, Xu S, Guo Y, et al. Epidemiological and Clinical Characteristics of Covid-19 in Adolescents and Young Adults. The Innovation. 2020;1(1):100001. doi: https://doi.org/10.1016/j.xinn.2020.04.001. PMID: 33554183.

6. Hu Z, Song C, Xu C, Jin G, Chen Y, Xu X, et al. Clinical Characteristics of 24 Asymptomatic Infections with Covid-19 Screened among Close Contacts in Nanjing, China. Sci China Life Sci. 2020;63(5):706-11. Epub 2020/03/09. doi: https://doi.org/10.1007/s11427-020-1661-4. PMID: 32146694; PubMed Central PMCID: PMC7088568.

7. Luo SH, Liu W, Liu ZJ, Zheng XY, Hong CX, Liu ZR, et al. A Confirmed Asymptomatic Carrier of 2019 Novel Coronavirus. Chin Med J (Engl). 2020;133(9):1123-5. Epub 2020/03/10. doi: https://doi.org/10.1097/CM9.0000000000000798. PMID: 32149768.

8. Chan JF, Yuan S, Kok KH, To KK, Chu H, Yang J, et al. A Familial Cluster of Pneumonia Associated with the 2019 Novel Coronavirus Indicating Person-to-Person Transmission: A Study of a Family Cluster. Lancet. 2020;395(10223):514-23. Epub 2020/01/28. doi: https://doi.org/10.1016/S0140-6736(20)30154-9. PMID: 31986261; PubMed Central PMCID: PMC7159286.

9. Ye F, Xu S, Rong Z, Xu R, Liu X, Deng P, et al. Delivery of Infection from Asymptomatic Carriers of Covid-19 in a Familial Cluster. Int J Infect Dis. 2020;94(NA):133-8. Epub 2020/04/06. doi: https://doi.org/10.1016/j.ijid.2020.03.042. PMID: 32247826; PubMed Central PMCID: PMC7129961.

10. Bai Y, Yao L, Wei T, Tian F, Jin DY, Chen L, et al. Presumed Asymptomatic Carrier Transmission of Covid-19. JAMA. 2020;54(0):E017. doi: https://doi.org/10.1001/jama.2020.2565. PMID: 32083643.

11. Luo Y, Trevathan E, Qian Z, Li Y, Li J, Xiao W, et al. Asymptomatic Sars-Cov-2 Infection in Household Contacts of a Healthcare Provider, Wuhan, China. Emerg Infect Dis. 2020. doi: https://doi.org/10.3201/eid2608.201016. PMID: 32330112.

12. Zhang J, Tian S, Lou J, Chen Y. Familial Cluster of Covid-19 Infection from an Asymptomatic. Crit Care. 2020;NA(NA). doi: https://doi.org/10.1186/s13054-020-2817-7. PMID: 32220236.

13. Zhang B, Liu S, Dong Y, Zhang L, Zhong Q, Zou Y, et al. Positive Rectal Swabs in Young Patients Recovered from Coronavirus Disease 2019 (Covid-19). J Infect. 2020. doi: https://doi.org/10.1016/j.jinf.2020.04.023. PMID: 32335176.

14. Huang L, Jiang J, Li X, Zhou Y, Xu M, Zhou J. Initial Ct Imaging Characters of an Imported Family Cluster of Covid-19. Clinical Imaging. 2020;65:78-81. doi: https://doi.org/10.1016/j.clinimag.2020.04.010. PMID: 32361413.

15. Qian G, Yang N, Ma AHY, Wang L, Li G, Chen X, et al. A Covid-19 Transmission within a Family Cluster by Presymptomatic Infectors in China. Clin Infect Dis. 2020. doi: https://doi.org/10.1093/cid/ciaa316. PMID: 32201889.

16. Gao Y, Shi C, Chen Y, Shi P, Liu J, Xiao Y, et al. A Cluster of the Corona Virus Disease 2019 Caused by Incubation Period Transmission in Wuxi, China. J Infect. 2020;80(6):666-70. doi: https://doi.org/10.1016/j.jinf.2020.03.042. PMID: 32283165.

17. Wang Z, Ma W, Zheng X, Wu G, Zhang R. Household Transmission of Sars-Cov-2. J Infect. 2020;81(1):179-82. doi: https://doi.org/10.1016/j.jinf.2020.03.040. PMID: 32283139.

18. Yang R, Gui X, Xiong Y. Comparison of Clinical Characteristics of Patients with Asymptomatic Vs Symptomatic Coronavirus Disease 2019 in Wuhan, China. JAMA Netw Open. 2020. doi: https://doi.org/10.1001/jamanetworkopen.2020.10182. PMID: 32459353.

19. Bi Q, Wu Y, Mei S, Ye C, Zou X, Zhang Z, et al. Epidemiology and Transmission of Covid-19 in 391 Cases and 1286 of Their Close Contacts in Shenzhen, China: A Retrospective Cohort Study. Lancet Infect Dis. 2020. Epub 2020/05/01. doi: https://doi.org/10.1016/S1473-3099(20)30287-5. PMID: 32353347; PubMed Central PMCID: PMC7185944.

20. Roxby AC, Greninger AL, Hatfield KM, Lynch JB, Dellit TH, James A, et al. Outbreak Investigation of Covid-19 among Residents and Staff of an Independent and Assisted Living Community for Older Adults in Seattle, Washington. JAMA Intern Med. 2020. doi: https://doi.org/10.1001/jamainternmed.2020.2233. PMID: 32437547.

21. Solbach W, Schiffner J, Backhaus I, Burger D, Staiger R, Tiemer B, et al. Antibody Profiling of Covid-19 Patients in an Urban Low-Incidence Region in Northern Germany. Frontiers in Public Health. 2020;8. doi: https://doi.org/10.3389/fpubh.2020.570543. PMID: 33072707.

22. Mizumoto K, Kagaya K, Zarebski A, Chowell G. Estimating the Asymptomatic Proportion of Coronavirus Disease 2019 (Covid-19) Cases on Board the Diamond Princess Cruise Ship, Yokohama, Japan, 2020. Euro Surveill. 2020;25(10). Epub 2020/03/19. doi: https://doi.org/10.2807/1560-7917.ES.2020.25.10.2000180. PMID: 32183930; PubMed Central PMCID: PMC7078829.

23. Tian S, Wu M, Chang Z, Wang Y, Zhou G, Zhang W, et al. Epidemiological Investigation and Intergenerational Clinical Characteristics of 24 Coronavirus Disease Patients Associated

with a Supermarket Cluster: A Retrospective Study. BMC Public Health. 2021;21(1):647. doi: https://doi.org/10.1186/s12889-021-10713-z. PMID.

24. Thai PQ, Rabaa MA, Luong DH, Tan DQ, Quang TD, Quach H-L, et al. The First 100 Days of Severe Acute Respiratory Syndrome Coronavirus 2 (Sars-Cov-2) Control in Vietnam. Clin Infect Dis. 2020;72(9):e334-e42. doi: https://doi.org/10.1093/cid/ciaa1130. PMID: 32738143.

25. Arima Y, Shimada T, Suzuki M, Suzuki T, Kobayashi Y, Tsuchihashi Y, et al. Severe Acute Respiratory Syndrome Coronavirus 2 Infection among Returnees to Japan from Wuhan, China, 2020. Emerg Infect Dis. 2020;26(7). Epub 2020/04/11. doi: https://doi.org/10.3201/eid2607.200994. PMID: 32275498.

26. Lytras T, Dellis G, Flountzi A, Hatzianastasiou S, Nikolopoulou G, Tsekou K, et al. High Prevalence of Sars-Cov-2 Infection in Repatriation Flights to Greece from Three European Countries. J Travel Med. 2020;27(3). Epub 2020/04/17. doi: https://doi.org/10.1093/jtm/taaa054. PMID: 32297940; PubMed Central PMCID: PMC7184451.

27. Lombardi A, Consonni D, Carugno M, Bozzi G, Mangioni D, Muscatello A, et al. Characteristics of 1,573 Healthcare Workers Who Underwent Nasopharyngeal Swab for Sars-Cov-2 in Milano, Lombardy, Italy. MedRxiv. 2020. doi: https://doi.org/10.1101/2020.05.07.20094276. PMID.

28. Pongpirul WA, Mott JA, Woodring JV, Uyeki TM, MacArthur JR, Vachiraphan A, et al. Clinical Characteristics of Patients Hospitalized with Coronavirus Disease, Thailand. Emerg Infect Dis. 2020;26(7). Epub 2020/04/09. doi: https://doi.org/10.3201/eid2607.200598. PMID: 32267826.

29. Qiu C, Deng Z, Xiao Q, Shu Y, Deng Y, Wang H, et al. Transmission and Clinical Characteristics of Coronavirus Disease 2019 in 104 Outside-Wuhan Patients, China. J Med Virol. 2020. doi: https://doi.org/10.1002/jmv.25975. PMID; PubMed Central PMCID: 32369217.

30. Zou L, Ruan F, Huang M, Liang L, Huang H, Hong Z, et al. Sars-Cov-2 Viral Load in Upper Respiratory Specimens of Infected Patients. N Engl J Med. 2020;382(12):1177-9. Epub 2020/02/20. doi: https://doi.org/10.1056/NEJMc2001737. PMID: 32074444; PubMed Central PMCID: PMC7121626.

31. Zhou R, Li F, Chen F, Liu H, Zheng J, Lei C, et al. Viral Dynamics in Asymptomatic Patients with Covid-19. Int J Infect Dis. 2020. doi: https://doi.org/10.1016/j.ijid.2020.05.030. PMID; PubMed Central PMCID: 32437933.

32. Chang MC, Hur J, Park D. Chest Computed Tomography Findings in Asymptomatic Patients with Covid-19. MedRxiv. 2020. doi: https://doi.org/10.1159/000509334. PMID: 32894853.

33. Zhou X, Li Y, Li T, Zhang W. Follow-up of Asymptomatic Patients with Sars-Cov-2 Infection. Clin Microbiol Infect. 2020. Epub 2020/04/03. doi: https://doi.org/10.1016/j.cmi.2020.03.024. PMID: 32234453.

34. Angelo Vaira L, Hopkins C, Salzano G, Petrocelli M, Melis A, Cucurullo M, et al. Olfactory and Gustatory Function Impairment in Covid-19 Patients: Italian Objective Multicenter-Study. Head Neck. 2020. doi: https://doi.org/10.1002/hed.26269. PMID: 32437022.

35. Wang X, Fang J, Zhu Y, Chen L, Ding F, Zhou R, et al. Clinical Characteristics of Non-Critically Ill Patients with Novel Coronavirus Infection (Covid-19) in a Fangcang Hospital. Clin Microbiol Infect. 2020. Epub 2020/04/07. doi: https://doi.org/10.1016/j.cmi.2020.03.032. PMID: 32251842; PubMed Central PMCID: PMC7195539.

36. Xu T, Huang R, Zhu L, Wang J, Cheng J, Zhang B, et al. Epidemiological and Clinical Features of Asymptomatic Patients with Sars-Cov-2 Infection. J Med Virol. 2020. doi: https://doi.org/10.1002/jmv.25944. PMID: 32346873.

37. Tabata S, Imai K, Kawano S, Ikeda M, Kodama T, Miyoshi K, et al. Non-Severe Vs Severe Symptomatic Covid-19: 104 Cases from the Outbreak on the Cruise Ship 'Diamond Princess' in Japan. medRxiv. 2020. doi: https://doi.org/10.1101/2020.03.18.20038125. PMID.

38. Noh JY, Yoon JG, Seong H, Choi WS, Sohn JW, Cheong HJ, et al. Asymptomatic Infection and Atypical Manifestations of Covid-19: Comparison of Viral Shedding Duration. J Infect. 2020. doi: https://doi.org/10.1016/j.jinf.2020.05.035. PMID: 32445728.

39. Kumar R, Bhattacharya B, Meena VP, Aggarwal A, Tripathi M, Soneja M, et al. Management of Mild Covid-19: Policy Implications of Initial Experience in India. MedRxiv. 2020. doi: https://doi.org/10.1101/2020.05.20.20107664. PMID.

40. Meng H, Xiong R, He R, Lin W, Hao B, Zhang L, et al. Ct Imaging and Clinical Course of Asymptomatic Cases with Covid-19 Pneumonia at Admission in Wuhan, China. J Infect. 2020;NA(NA). Epub 2020/04/16. doi: https://doi.org/10.1016/j.jinf.2020.04.004. PMID: 32294504; PubMed Central PMCID: PMC7152865.

41. Xiao T, Wang Y, Yuan J, Ye H, Wei L, Liao X, et al. Early Viral Clearance and Antibody Kinetics of Covid-19 among Asymptomatic Carriers. Front Med 2021;8. doi: https://doi.org/10.3389/fmed.2021.595773. PMID: 33791320.

42. Al-Shamsi HO, Coomes EA, Alrawi S. Screening for Covid-19 in Asymptomatic Patients with Cancer in a Hospital in the United Arab Emirates. JAMA Oncol. 2020. doi: https://doi.org/10.1001/jamaoncol.2020.2548. PMID: 32459297.

43. Wang Y, Tong J, Qin Y, Xie T, Li J, Li J, et al. Characterization of an Asymptomatic Cohort of Sars-Cov-2 Infected Individuals Outside of Wuhan, China. Clin Infect Dis. 2020. doi: https://doi.org/10.1093/cid/ciaa629. PMID: 32442265.

44. See KC, Liew SM, Ng DCE, Chew EL, Khoo EM, Sam CH, et al. Covid-19: Four Paediatric Cases in Malaysia. Int J Infect Dis. 2020;94(NA):125-7. Epub 2020/04/19. doi: https://doi.org/10.1016/j.ijid.2020.03.049. PMID: 32304822; PubMed Central PMCID: PMC7158792.

45. Tan YP, Tan BY, Pan J, Wu J, Zeng SZ, Wei HY. Epidemiologic and Clinical Characteristics of 10 Children with Coronavirus Disease 2019 in Changsha, China. J Clin Virol. 2020;127(NA):104353. Epub 2020/04/18. doi: https://doi.org/10.1016/j.jcv.2020.104353. PMID: 32302953; PubMed Central PMCID: PMC7195108.

46. Tan X, Huang J, Zhao F, Zhou Y, Li JQ, Wang XY. [Clinical Features of Children with Sars-Cov-2 Infection: An Analysis of 13 Cases from Changsha, China]. Zhongguo Dang Dai Er Ke Za Zhi. 2020. doi: https://doi.org/10.7499/j.issn.1008-8830.2003199. PMID: 32312364.

47. Melgosa M, Madrid A, Alvárez O, Lumbreras J, Nieto F, Parada E, et al. Sars-Cov-2 Infection in Spanish Children with Chronic Kidney Pathologies. Pediatr Nephrol. 2020. doi: 10.1007/s00467-020-04597-1. PMID.

48. Wu HP, Li BF, Chen X, Hu HZ, Jiang SA, Cheng H, et al. Clinical Features of Coronavirus Disease 2019 in Children Aged <18 Years in Jiangxi, China: An Analysis of 23 Cases. Zhongguo Dang Dai Er Ke Za Zhi. 2020;22(5):419-24. doi: https://doi.org/10.7499/j.issn.1008-8830.2003202. PMID: 32434634.

49. Song W, Li J, Zou N, Guan W, Pan J, Xu W. Clinical Features of Pediatric Patients with Coronavirus Disease (Covid-19). J Clin Virol. 2020;127(104377). doi: https://doi.org/10.1016/j.jcv.2020.104377. PMID; PubMed Central PMCID: 32361323.

50. Bai K, Liu W, Liu C, Fu Y, Hu J, Qin Y, et al. Clinical Analysis of 25 Novel Coronavirus Infections in Children. Pediatr Infect Dis J. 2020. doi: https://doi.org/10.1097/inf.0000000000002740. PMID: 32520888.

51. Xu H, Liu E, Xie J, Smyth RL, Zhou Q, Zhao R, et al. A Follow-up Study of Children Infected with Sars-Cov-2 from Western China. Ann Transl Med. 2020;8(10):623. doi: https://doi.org/10.21037/atm-20-3192. PMID: 32566560.

52. Qiu H, Wu J, Hong L, Luo Y, Song Q, Chen D. Clinical and Epidemiological Features of 36 Children with Coronavirus Disease 2019 (Covid-19) in Zhejiang, China: An Observational Cohort Study. Lancet Infect Dis. 2020;NA(NA). Epub 2020/03/30. doi: https://doi.org/10.1016/S1473-3099(20)30198-5. PMID: 32220650; PubMed Central PMCID: PMC7158906.

53. Lu Y, Li Y, Deng W, Liu M, He Y, Huang L, et al. Symptomatic Infection Is Associated with Prolonged Duration of Viral Shedding in Mild Coronavirus Disease 2019: A Retrospective Study of 110 Children in Wuhan. Pediatr Infect Dis J. 2020. doi: https://doi.org/10.1097/inf.0000000000002729. PMID: 32379191.

54. Merza MA, Haleem Al Mezori AA, Mohammed HM, Abdulah DM. Covid-19 Outbreak in Iraqi Kurdistan: The First Report Characterizing Epidemiological, Clinical, Laboratory, and Radiological Findings of the Disease. Diabetes Metab Syndr Clin Res Rev. 2020;14(4):547-54. doi: https://doi.org/10.1016/j.dsx.2020.04.047. PMID: 32408119.

55. Yongchen Z, Shen H, Wang X, Shi X, Li Y, Yan J, et al. Different Longitudinal Patterns of Nucleic Acid and Serology Testing Results Based on Disease Severity of Covid-19 Patients. Emerg Microbes Infect. 2020;9(1):833-6. doi: https://doi.org/10.1080/22221751.2020.1756699. PMID: 32306864.

56. Migisha R, Kwesiga B, Mirembe BB, Amanya G, Kabwama SN, Kadobera D, et al. Early Cases of Sars-Cov-2 Infection in Uganda: Epidemiology and Lessons Learned from Risk-Based Testing Approaches - March-April 2020. Global Health. 2020;16(1):114. Epub 2020/11/27. doi: https://doi.org/10.1186/s12992-020-00643-7. PMID: 33239041; PubMed Central PMCID: 7686950.

57. Kim SE, Jeong HS, Yu Y, Shin SU, Kim S, Oh TH, et al. Viral Kinetics of Sars-Cov-2 in Asymptomatic Carriers and Presymptomatic Patients. Int J Infect Dis. 2020;95:441-3. doi: https://dx.doi.org/10.1016%2Fj.ijid.2020.04.083. PMID: 32376309.

58. Choe PG, Kang EK, Lee SY, Oh B, Im D, Lee HY, et al. Selecting Coronavirus Disease 2019 Patients with Negligible Risk of Progression: Early Experience from Non-Hospital Isolation Facility in Korea. Korean J Intern Med. 2020;35(4):765-70. doi: https://doi.org/10.3904/kjim.2020.159. PMID: 32460457.

59. Sharma AK, Ahmed A, Baig VN, Dhakad P, Dalela G, Kacker S, et al. Characteristics and Outcomes of Hospitalized Young Adults with Mild to Moderate Covid-19 at a University Hospital in India. MedRxiv. 2020. doi: https://doi.org/10.1101/2020.06.02.20106310. PMID.

60. Zhang W, Long Q, Huang Y, Chen C, Wu J, Hong Y, et al. Asymptomatic Covid-19 Have Longer Treatment Cycle Than Moderate Type of Confirmed Patients. MedRxiv. 2020. doi: https://doi.org/10.1101/2020.05.16.20103796. PMID.

61. Alshami AA, Alattas RA, Anan HF, Al Qahtani HS, Al Mulhim MA, Alahilmi AA, et al. Silent Disease and Loss of Taste and Smell Are Common Manifestations of Sars-Cov-2 Infection in a Quarantine Facility: First Report from Saudi Arabia. MedRxiv. 2020. doi: https://doi.org/10.3201/eid2605.200198. PMID.

62. Kong W, Wang Y, Hu J, Chughtai A, Pu H. Comparison of Clinical and Epidemiological Characteristics of Asymptomatic and Symptomatic Sars-Cov-2 Infection: A Multi-Center Study in Sichuan Province, China. Travel Med Infect Dis. 2020;37(101754). doi: https://dx.doi.org/10.1016%2Fj.tmaid.2020.101754. PMID: 32492485.

63. Wang Y, Liu Y, Liu L, Wang X, Luo N, Ling L. Clinical Outcome of 55 Asymptomatic Cases at the Time of Hospital Admission Infected with Sars-Coronavirus-2 in Shenzhen, China. J Infect Dis. 2020;221(11):1170-774. Epub 2020/03/18. doi: https://doi.org/10.1093/infdis/jiaa119. PMID: 32179910; PubMed Central PMCID: PMC7184401.

64. Ganyani T, Kremer C, Chen D, Torneri A, Faes C, Wallinga J, et al. Estimating the Generation Interval for Coronavirus Disease (Covid-19) Based on Symptom Onset Data, March 2020. Euro Surveill. 2020;25(17):2020.03.05.20031815. Epub 2020/05/07. doi: https://doi.org/10.2807/1560-7917.es.2020.25.17.2000257. PMID: 32372755; PubMed Central PMCID: PMC7201952.

65. Park SY, Kim YM, Yi S, Lee S, Na BJ, Kim CB, et al. Coronavirus Disease Outbreak in Call Center, South Korea. Emerg Infect Dis. 2020;26(8):1666-70. Epub 2020/04/24. doi: https://doi.org/10.3201/eid2608.201274. PMID: 32324530; PubMed Central PMCID: 7392450.

66. Emery JC, Russel TW, Liu Y, Hellewell J, Pearson CA, group Cnw, et al. The Contribution of Asymptomatic Sars-Cov-2 Infections to Transmission - a Model-Based Analysis of the Diamond Princess Outbreak. MedRxiv. 2020. doi: https://doi.org/10.1101/2020.05.07.20093849. PMID.

67. Casey M, Griffin J, McAloon CG, Byrne AW, Madden JM, McEvoy D, et al. Estimating Pre-Symptomatic Transmission of Covid-19: A Secondary Analysis Using Published Data. MedRxiv. 2020. doi: https://doi.org/10.1101/2020.05.08.20094870. PMID.
