# Supplementary material for: Occurrence and transmission potential of asymptomatic and presymptomatic SARS-CoV-2 infections: Update of a living systematic review and meta-analysis
Source: PLoS Med. 2022 May 26;19(5):e1003987. doi: 10.1371/journal.pmed.1003987 (PMC9135333; doi:10.1371/journal.pmed.1003987)
Supplement: S3 Table — (PDF) [file pmed.1003987.s008.pdf]

S3 Table. Location of studies contributing data to review question 1

Country	Total SARS-CoV-2, n	Total asymptomatic SARS-CoV-2, n	Total number of studies
United States of America	9,725	4,004	37
United Kingdom	2,338	590	12
China	3,881	2,021	11
Italy	537	127	7
Germany	100	9	6
South Korea	928	95	4
Spain	86	24	4
Canada	53	15	3
India	2,251	1,373	3
Japan	150	50	3
Bahrain	194	119	2
Brazil	42	6	2
France	173	15	2
Netherlands	192	11	2
Pakistan	280	66	2
South Africa	227	55	2
Turkey	687	64	2
Uganda	82	45	2
Argentina	113	75	1
Australia	171	61	1
Austria	4	2	1
Bangladesh	26	7	1
Belgium	4	2	1
Brunei	138	16	1
Colombia	35	11	1

Czech Republic	105	6	1
Ethiopia	2,617	1,935	1
Finland	127	23	1
French Guyana	137	87	1
Greece	46	7	1
Iceland	178	25	1
Iran	21	14	1
Ireland	1,105	290	1
Ivory Coast	54	18	1
Kuwait	1,096	473	1
Malaysia	46	6	1
Norway	40	0	1
Other	22	4	1
Portugal	14	0	1
Saudi Arabia	18	12	1
Switzerland	175	71	1
Vietnam	208	89	1
