# Supplementary material for: Occurrence and transmission potential of asymptomatic and presymptomatic SARS-CoV-2 infections: Update of a living systematic review and meta-analysis
Source: PLoS Med. 2022 May 26;19(5):e1003987. doi: 10.1371/journal.pmed.1003987 (PMC9135333; doi:10.1371/journal.pmed.1003987)
Supplement: S4 Table — (PDF) [file pmed.1003987.s009.pdf]

S4 Table. Summary of findings from subgroup analyses in studies estimating the proportion of asymptomatic SARS-CoV-2 infections

	Contact and outbreak investigations						Screening of defined population					
**Domain**	**n[a]**	**Summary (95% CI)**	**Prediction interval**	**I^2, %**	**τ^2**	**Subgroup difference, p value**	**n[b]**	**Summary (95% CI)**	**Prediction interval**	**I^2, %**	**τ^2**	**Subgroup difference p value**
**Selection bias[b]**												
Low risk	27	0.24 (0.17-0.33)	0.04-0.74	84	1.05	0.097	27	0.46 (0.33-0.59)	0.05-0.94	98	1.85	0.597
Unclear/ high risk	26	0.15 (0.10-0.23)	0.02-0.68	88	1.36		61	0.41 (0.31;0.52)	0.03-0.94	97	2.45	
**Information bias, assessment of symptoms defining status[a]**												
Low risk	12	0.18 (0.08-0.35)	0.01-0.86	85	2.07	0.794	21	0.29 (0.20-0.42)	0.03-0.83	98	1.33	0.026
Unclear/ high risk	41	0.20 (0.15;0.26)	0.03-0.67	86	1.07		67	0.47 (0.37;0.57)	0.04-0.95	97	2.47	
**Information bias, misclassification based on follow-up[a]**												
Low risk	39	0.18 (0.13-0.26)	0.02-0.76	88	1.64	0.241	68	0.41 (0.32-0.51)	0.03-0.94	97	2.31	0.554
Unclear/ high risk	14	0.24 (0.18-0.32)	0.07-0.55	88	0.35		20	0.47 (0.31-0.63)	0.04-0.95	97	1.95	
**Selective reporting bias[a]**												
Low risk	47	0.20 (0.15-0.26)	0.03-0.69	90	1.18	0.758	79	0.44 (0.35-0.53)	0.03-0.95	97	2.406	0.242
Unclear/ high risk	6	0.17 (0.06-0.38)	0.00-0.92	92	1.74		9	0.32 (0.18-0.50)	0.03-0.87	97	1.08	
**All domains**												
Low risk	6	0.20 (0.09-0.39)	0.01-0.85	92	1.01	0.881	6	0.25 (0.10-0.51)	0.01-0.94	99	1.67	0.152
Unclear/ high risk	47	0.19 (0.14-0.25)	0.02-0.71	86	1.31		47	0.44 (0.36-0.53)	0.04-0.94	96	2.25	

[a] n = number of clusters analysed, which exceeds the total number of studies
[b] Assessed in the risk of bias tool (S2 Text), with full assessments in S3 Fig;
