# Supplementary material for: Occurrence and transmission potential of asymptomatic and presymptomatic SARS-CoV-2 infections: Update of a living systematic review and meta-analysis
Source: PLoS Med. 2022 May 26;19(5):e1003987. doi: 10.1371/journal.pmed.1003987 (PMC9135333; doi:10.1371/journal.pmed.1003987)
Supplement: S5 Table — CI, confidence interval; NPI, non-pharmaceutical intervention; SARS-CoV-2, Severe Acute Respiratory Syndrome Coronavirus 2. (PDF) [file pmed.1003987.s010.pdf]

S5 Table. Characteristics of mathematical modelling studies and methods for estimation of the contribution of asymptomatic and presymptomatic infection to SARS-CoV-2 transmission

First author, publication year [ref]	Original data analysed	Method/sources	Comments	Level of evidence
Ferretti L, 2020 [1]	40 transmission pairs, publicly available sources, China. Used to estimate generation time	Incubation period: Lauer et al. 2020 [2]<br>Serial interval estimated	Original data: “manually selected according to high confidence of direct transmission inferred from publicly available sources at the time of writing (March 2020), and with known time of onset of symptoms for both source and recipient.”<br>Control measures: "effect of control measures discussed later will be relative to the early stages of an outbreak”	Moderate
Emery JC, 2020 [3]	Data from the Diamond Princess outbreak, 20 Jan-14 Feb 2020, extracted from Mizumoto et al. [4] Nishiura et al. [5].	Deterministic, compartmental model.<br>Latent period: 4.3 days, duration of presymptomatic state: 2.1 days, Backer et al. [6]; duration of asymptomatic state: 5 days (assumed).	The model is fitted to two sets of data: the number symptomatic cases and the cases detected from extensive testing of individuals regardless of symptoms.<br>The model estimates the decreases in transmissibility for pre- and asymptomatic individuals. However, the wide CI in the estimate of contribution of asymptomatic infections to transmission shows an issue in identifying such parameters.	Low/moderate
Zhang W, 2020 [7]	No original data analysed.	Incubation period: Li et al. 2020 [8]<br>Serial interval: Li et al. 2020 [8]	Scenario 1: early transmission in Wuhan (published data from cases before 20 January 2020)	Low
		Incubation period: Backer et al. 2020 [6]<br>Serial interval: Du et al. 2020 [9]	Scenario 2: Imported cases outside Wuhan (published data from cases 21 January – 8 February 2020)	

First author, publication year [ref]	Original data analysed	Method/sources	Comments	Level of evidence
			Control measures: "(the Wuhan lockdown was initiated on Jan. 23, 2020)." "The dramatic change of infection time distribution between these two scenarios may due to effective case isolation and quarantine of people with Wuhan travel history which could significantly reduce transmissions after symptom onset."	
He X, 2020 [10]	77 transmission pairs, publicly available sources within and outside mainland China. Used to estimate serial interval.	Incubation period taken from Li et al. 2020 [8]<br>Serial interval estimated.	Original data: from several different countries in SE Asia, Europe. Dates of contact 13 January 2020 – 25 February, where reported.<br>Control measures in place at the time of data collection and heterogeneity between sources for transmission pairs not discussed.	Moderate
Peak CM, 2020 [11]	No original data	Estimate the infectiousness profile for two scenarios.<br>Incubation period: Li et al. 2020 [8]<br>Serial interval: Nishiura et al 2020 [5]	Scenario 1: Normal serial interval	Low
		Incubation period: Li et al. 2020 [8]<br>Serial interval: Li et al. 2020 [8]	Scenario 2: Long serial interval<br>Control measures: reliability of input parameters mentioned as limitation, but not in terms of the control measures in place at the time of the original data collection	
Tindale LC, 2021 [12]	54 transmission pairs in Singapore	Serial interval and incubation period estimated	They considered that incubation and serial interval are dependent.<br>Changes in estimates of incubation over time suggests the presence of intermediate cases.	High (but no CI)
	80 transmission pairs in Tianjin, China			

First author, publication year [ref]	Original data analysed	Method/sources	Comments	Level of evidence
			Relatively short serial interval estimated, reflecting the contact tracing implementation.<br>Data from transmission pairs used in previous publication [13]<br>Control measures: described for each setting. Tianjin measures stronger than Singapore	
Moghadas SM, 2020 [14]	No original data	Incubation: Li et al. 2020 [8]<br>Presymptomatic period: Li et al. 2020 [15]<br>Infectious period from onset of symptom: He X et al. 2020 [10]	Scenario 1: Asymptomatic: 17.9% [4]<br>Scenario 2: Asymptomatic: 30.8% [5]<br>Control measures: not discussed	Low
Ren X, 2021 [16]	80 transmission pairs, infectors were people who had visited Wuhan, China; infectees were outside Hubei province	For the 55 pairs where transmission could have happened before or after symptoms, Monte Carlo simulations to estimate whether the chance of asymptomatic transmission is >50%	No mathematical model, they counted the proportion of infections from presymptomatic.<br>Control measures: “We restricted the study participants to COVID-19 cases reported outside Hubei Province in the early stage of outbreaks in China, before any community transmission had occurred in these areas.”	High (but no CI)
Chun JY, 2021 [17]	72 transmission pairs, South Korea, until 31 March	Bayesian method to infer the infectiousness profile	Very short incubation period and serial interval estimated (2.9 and 3.6 days respectively).<br>Control measures: not discussed	High
Bushman M, 2021 [18]	873 transmission pairs in China, before NPIs	Serial interval is estimated. The incubation period prior: Lauer et al. 2020 [2], Zhang et al. 2020 [19] and Backer et al. 2020 [6]	Also assumed an incubation-dependent model (incubation longer when generation interval longer).<br>The data includes some data from He X [10].<br>Control measures: “We divided case pairs into two time periods using the symptom onset	Moderate

First author, publication year [ref]	Original data analysed	Method/sources	Comments	Level of evidence
			dates of the primary cases. January 23 marked the lockdown of Wuhan and the start of a national rollout of nonpharmaceutical interventions (NPIs)"	
Sun K, 2020 [20]	Contact tracing data, Hunan China, before lockdown	The infectious profile (Fig 3E) is inferred from the transmission pairs.	Control measures: "Risk is further stratified by the date of implementation of social distancing interventions in Hunan, which is 25 January 2020."	High (but no CI)
Wu P, 2021 [21]	96 transmission pairs, contact tracing data from 4 provinces and 1 municipality in China	The authors used the same method as described by He et al. (see above). Incubation period taken from Li et al. 2020 [8].<br>Serial interval estimated.	Regarding the impact of control measure on the estimate, the authors acknowledged that "the estimate is likely to be the upper limit of contribution to the overall infections since further transmission might have been interrupted by isolation of confirmed cases depending on the efficiency in case finding" but added that their estimate "suffered little from such interruption".	Moderate
Tan J, 2021 [22]	All confirmed cases with symptom status, January 7th to February 21st, 2020, Zhejiang province, China (asymptomatic or symptomatic)	Age-stratified compartmental model with distinction between presymptomatic, asymptomatic and symptomatic infections.<br>Main assumptions: Latent period of 2 days (Lauer et al. [2], Backer et al. [6]), period of presymptomatic transmission: 3-4 days (Kong et al. [23], Wei et al.) [24].	The authors estimated the proportion of asymptomatic infections (accounting for unconfirmed asymptomatic infections) and the reduced transmissibility of asymptomatics, both by age. They could then estimate the contribution of asymptomatic to infections, overall and by age.<br>Limitation: The model involves many parameters that are estimated with only the reported number of symptomatic and asymptomatic infections, which questions about the identifiability of all the parameters	Low/moderate

CI, confidence interval; NPI, non-pharmaceutical intervention.

References

1. Ferretti L, Wymant C, Kendall M, Zhao L, Nurtay A, Abeler-Dorner L, et al. Quantifying Sars-Cov-2 Transmission Suggests Epidemic Control with Digital Contact Tracing. Science. 2020;368(6491). Epub 2020/04/03. https://doi.org/10.1126/science.abb6936. PubMed PMID: 32234805; PubMed Central PMCID: PMC7164555.

2. Lauer SA, Grantz KH, Bi Q, Jones FK, Zheng Q, Meredith HR, et al. The Incubation Period of Coronavirus Disease 2019 (Covid-19) from Publicly Reported Confirmed Cases: Estimation and Application. Ann Intern Med. 2020;172(9):577-82. Epub 20200310. https://doi.org/10.7326/m20-0504. PubMed PMID: 32150748; PubMed Central PMCID: PMC7081172.

3. Emery JC, Russell TW, Liu Y, Hellewell J, Pearson CA, Group CC-W, et al. The Contribution of Asymptomatic Sars-Cov-2 Infections to Transmission on the Diamond Princess Cruise Ship. Elife. 2020;9. Epub 2020/08/25. https://doi.org/10.7554/elife.58699. PubMed PMID: 32831176; PubMed Central PMCID: 7527238.

4. Mizumoto K, Kagaya K, Zarebski A, Chowell G. Estimating the Asymptomatic Proportion of Coronavirus Disease 2019 (Covid-19) Cases on Board the Diamond Princess Cruise Ship, Yokohama, Japan, 2020. Euro Surveill. 2020;25(10). Epub 2020/03/19. https://doi.org/10.2807/1560-7917.es.2020.25.10.2000180. PubMed PMID: 32183930; PubMed Central PMCID: 7078829.

5. Nishiura H, Kobayashi T, Miyama T, Suzuki A, Jung SM, Hayashi K, et al. Estimation of the Asymptomatic Ratio of Novel Coronavirus Infections (Covid-19). Int J Infect Dis. 2020;94:154-5. Epub 20200314. https://doi.org/10.1016/j.ijid.2020.03.020. PubMed PMID: 32179137; PubMed Central PMCID: PMC7270890.

6. Backer JA, Klinkenberg D, Wallinga J. Incubation Period of 2019 Novel Coronavirus (2019-Ncov) Infections among Travellers from Wuhan, China, 20-28 January 2020. Euro Surveill. 2020;25(5). https://doi.org/10.2807/1560-7917.es.2020.25.5.2000062. PubMed PMID: 32046819; PubMed Central PMCID: PMC7014672.

7. Zhang W. Estimating the Presymptomatic Transmission of Covid19 Using Incubation Period and Serial Interval Data. bioRxiv [Preprint]. 2020. https://doi.org/10.1101/2020.04.02.20051318.

8. Li Q, Guan X, Wu P, Wang X, Zhou L, Tong Y, et al. Early Transmission Dynamics in Wuhan, China, of Novel Coronavirus-Infected Pneumonia. N Engl J Med. 2020;382(13):1199-207. Epub 2020/01/30. https://doi.org/10.1056/nejmoa2001316. PubMed PMID: 31995857; PubMed Central PMCID: PMC7121484.

9. Du Z, Xu X, Wu Y, Wang L, Cowling BJ, Meyers LA. Serial Interval of Covid-19 among Publicly Reported Confirmed Cases. Emerg Infect Dis. 2020;26(6):1341-3. Epub 20200617. https://doi.org/10.3201/eid2606.200357. PubMed PMID: 32191173; PubMed Central PMCID: PMC7258488.

10. He X, Lau EHY, Wu P, Deng X, Wang J, Hao X, et al. Temporal Dynamics in Viral Shedding and Transmissibility of Covid-19. Nat Med. 2020;26(5):672-5. Epub 2020/04/17. https://doi.org/10.1038/s41591-020-0869-5. PubMed PMID: 32296168.

11. Peak CM, Kahn R, Grad YH, Childs LM, Li R, Lipsitch M, et al. Individual Quarantine Versus Active Monitoring of Contacts for the Mitigation of Covid-19: A Modelling Study. Lancet Infect Dis. 2020;20(9):1025-33. Epub 2020/05/24. https://doi.org/10.1016/s1473-3099(20)30361-3. PubMed PMID: 32445710; PubMed Central PMCID: 7239635.

12. Tindale LC, Stockdale JE, Coombe M, Garlock ES, Lau WYV, Saraswat M, et al. Evidence for Transmission of Covid-19 Prior to Symptom Onset Elife. 2020;9. Epub 2020/06/23. https://doi.org/10.7554/elife.57149. PubMed PMID: 32568070; PubMed Central PMCID: 7386904.

13. Ganyani T, Kremer C, Chen D, Torneri A, Faes C, Wallinga J, et al. Estimating the Generation Interval for Coronavirus Disease (Covid-19) Based on Symptom Onset Data, March 2020. Euro Surveill. 2020;25(17):2020.03.05.20031815. Epub 2020/05/07. https://doi.org/10.2807/1560-7917.es.2020.25.17.2000257. PubMed PMID: 32372755; PubMed Central PMCID: 7201952.

14. Moghadas SM, Fitzpatrick MC, Sah P, Pandey A, Shoukat A, Singer BH, et al. The Implications of Silent Transmission for the Control of Covid-19 Outbreaks. Proc Natl Acad Sci U S A. 2020;117(30):17513-5. Epub 2020/07/08. https://doi.org/10.1073/pnas.2008373117. PubMed PMID: 32632012; PubMed Central PMCID: 7395516.

15. Li R, Pei S, Chen B, Song Y, Zhang T, Yang W, et al. Substantial Undocumented Infection Facilitates the Rapid Dissemination of Novel Coronavirus (Sars-Cov-2). Science. 2020;368(6490):489-93. Epub 20200316. https://doi.org/10.1126/science.abb3221. PubMed PMID: 32179701; PubMed Central PMCID: PMC7164387.

16. Ren X, Li Y, Yang X, Li Z, Cui J, Zhu A, et al. Evidence for Pre-Symptomatic Transmission of Coronavirus Disease 2019 (Covid-19) in China. Influenza Other Respir Viruses. 2021;15(1):19-26. Epub 2020/08/09. https://doi.org/10.1111/irv.12787. PubMed PMID: 32767657; PubMed Central PMCID: 7436222.

17. Chun JY, Baek G, Kim Y. Transmission Onset Distribution of Covid-19. Int J Infect Dis. 2020;99:403-7. Epub 2020/08/11. https://doi.org/10.1016/j.ijid.2020.07.075. PubMed PMID: 32771633; PubMed Central PMCID: 7409940.

18. Bushman M, Worby C, Chang HH, Kraemer MUG, Hanage WP. Transmission of Sars-Cov-2 before and after Symptom Onset: Impact of Nonpharmaceutical Interventions in China. Eur J Epidemiol. 2021;36(4):429-39. Epub 2021/04/22. https://doi.org/10.1007/s10654-021-00746-4. PubMed PMID: 33881667; PubMed Central PMCID: 8058147.

19. Zhang J, Litvinova M, Wang W, Wang Y, Deng X, Chen X, et al. Evolving Epidemiology and Transmission Dynamics of Coronavirus Disease 2019 Outside Hubei Province, China: A Descriptive and Modelling Study. Lancet Infect Dis. 2020;20(7):793-802. Epub 20200402. https://doi.org/10.1016/s1473-3099(20)30230-9. PubMed PMID: 32247326; PubMed Central PMCID: PMC7269887.

20. Sun K, Wang W, Gao L, Wang Y, Luo K, Ren L, et al. Transmission Heterogeneities, Kinetics, and Controllability of Sars-Cov-2. Science. 2021;371(6526). Epub 2020/11/26. https://doi.org/10.1126/science.abe2424. PubMed PMID: 33234698; PubMed Central PMCID: 7857413.

21. Wu P, Liu F, Chang Z, Lin Y, Ren M, Zheng C, et al. Assessing Asymptomatic, Presymptomatic, and Symptomatic Transmission Risk of Severe Acute Respiratory Syndrome Coronavirus 2. Clin Infect Dis. 2021;73(6):e1314-e20. https://doi.org/10.1093/cid/ciab271. PubMed PMID: 33772573; PubMed Central PMCID: PMC8083716.

22. Tan J, Ge Y, Martinez L, Sun J, Li C, Westbrook A, et al. Transmission Roles of Symptomatic and Asymptomatic Covid-19 Cases: A Modeling Study. medRxiv [Preprint]. 2021:2021.05.11.21257060. https://doi.org/10.1101/2021.05.11.21257060.

23. Kong D, Zheng Y, Wu H, Pan H, Wagner AL, Zheng Y, et al. Pre-Symptomatic Transmission of Novel Coronavirus in Community Settings. Influenza and other respiratory viruses. 2020;14(6):610-4. Epub 2020/06/19. https://dx.doi.org/10.1111%2Firv.12773. PubMed PMID: 32558175.

24. Wei WE, Li Z, Chiew CJ, Yong SE, Toh MP, Lee VJ. Presymptomatic Transmission of Sars-Cov-2 - Singapore, January 23-March 16, 2020. MMWR Morb Mortal Wkly Rep. 2020;69(14):411-5. Epub 2020/04/10. http://dx.doi.org/10.15585/mmwr.mm6914e1. PubMed PMID: 32271722; PubMed Central PMCID: 7147908
