# Supplementary material for: Occurrence and transmission potential of asymptomatic and presymptomatic SARS-CoV-2 infections: Update of a living systematic review and meta-analysis
Source: PLoS Med. 2022 May 26;19(5):e1003987. doi: 10.1371/journal.pmed.1003987 (PMC9135333; doi:10.1371/journal.pmed.1003987)
Supplement: S1 Fig — (PDF) [file pmed.1003987.s011.pdf]

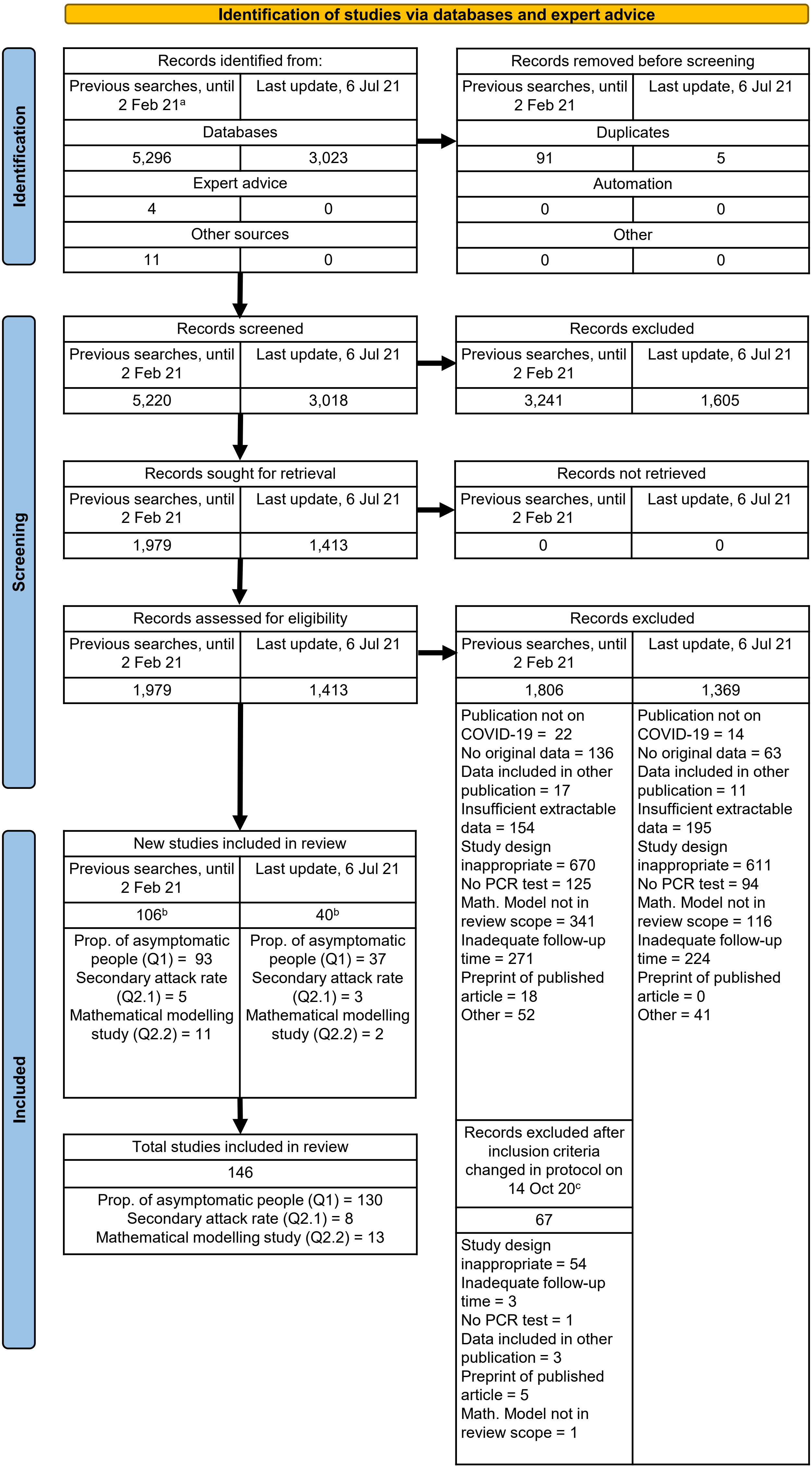

S1 Figure. Flowchart of identified, excluded and included records as of 6 July 2021

COVID-19, Coronavirus disease 2019; Math., mathematical; Prop., proportion;

[a]We also included papers that were published after the search date if we had previously identified their preprint.

[b]Note that some studies provided information for more than one question, therefore the total number is less than the sum of the study types.

[c]The protocol was updated on 18 Jun 21 and the study questions and inclusion criteria were updated. See S1 Table for more detail on these excluded studies.
