# Supplementary material for: Occurrence and transmission potential of asymptomatic and presymptomatic SARS-CoV-2 infections: Update of a living systematic review and meta-analysis
Source: PLoS Med. 2022 May 26;19(5):e1003987. doi: 10.1371/journal.pmed.1003987 (PMC9135333; doi:10.1371/journal.pmed.1003987)
Supplement: S2 Fig — (PDF) [file pmed.1003987.s012.pdf]

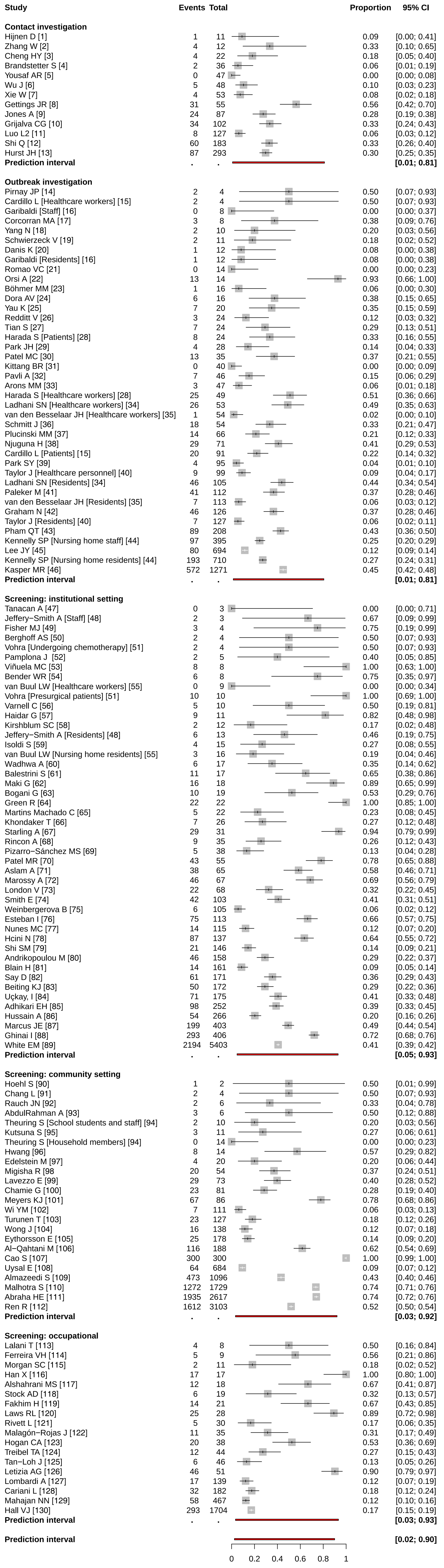
Study Events Total Proportion 95% CI
Contact investigation
Hijnen D [1] 1 11 0.09 [0.00; 0.41]
Zhang W [2] 4 12 0.33 [0.10; 0.65]
Cheng HY [3] 4 22 0.18 [0.05; 0.40]
Brandstetter S [4] 2 36 0.06 [0.01; 0.19]
Yousaf AR [5] 0 47 0.00 [0.00; 0.08]
Wu J [6] 5 48 0.10 [0.03; 0.23]
Xie W [7] 4 53 0.08 [0.02; 0.18]
Gettings JR [8] 31 55 0.56 [0.42; 0.70]
Jones A [9] 24 87 0.28 [0.19; 0.38]
Grijalva CG [10] 34 102 0.33 [0.24; 0.43]
Luo L2 [11] 8 127 0.06 [0.03; 0.12]
Shi Q [12] 60 183 0.33 [0.26; 0.40]
Hurst JH [13] 87 293 0.30 [0.25; 0.35]
Prediction interval . . [0.01; 0.81]
Outbreak investigation
Pirnay JP [14] 2 4 0.50 [0.07; 0.93]
Cardillo L [Healthcare workers] [15] 2 4 0.50 [0.07; 0.93]
Garibaldi [Staff] [16] 0 8 0.00 [0.00; 0.37]
Corcorran MA [17] 3 8 0.38 [0.09; 0.76]
Yang N [18] 2 10 0.20 [0.03; 0.56]
Schwierzeck V [19] 2 11 0.18 [0.02; 0.52]
Danis K [20] 1 12 0.08 [0.00; 0.38]
Garibaldi [Residents] [16] 1 12 0.08 [0.00; 0.38]
Romao VC [21] 0 14 0.00 [0.00; 0.23]
Orsi A [22] 13 14 0.93 [0.66; 1.00]
Böhmer MM [23] 1 16 0.06 [0.00; 0.30]
Dora AV [24] 6 16 0.38 [0.15; 0.65]
Yau K [25] 7 20 0.35 [0.15; 0.59]
Redditt V [26] 3 24 0.12 [0.03; 0.32]
Tian S [27] 7 24 0.29 [0.13; 0.51]
Harada S [Patients] [28] 8 24 0.33 [0.16; 0.55]
Park JH [29] 4 28 0.14 [0.04; 0.33]
Patel MC [30] 13 35 0.37 [0.21; 0.55]
Kittang BR [31] 0 40 0.00 [0.00; 0.09]
Pavli A [32] 7 46 0.15 [0.06; 0.29]
Arons MM [33] 3 47 0.06 [0.01; 0.18]
Harada S [Healthcare workers] [28] 25 49 0.51 [0.36; 0.66]
Ladhani SN [Healthcare workers] [34] 26 53 0.49 [0.35; 0.63]
van den Besselaar JH [Healthcare workers] [35] 1 54 0.02 [0.00; 0.10]
Schmitt J [36] 18 54 0.33 [0.21; 0.47]
Plucinski MM [37] 14 66 0.21 [0.12; 0.33]
Njuguna H [38] 29 71 0.41 [0.29; 0.53]
Cardillo L [Patients] [15] 20 91 0.22 [0.14; 0.32]
Park SY [39] 4 95 0.04 [0.01; 0.10]
Taylor J [Healthcare personnel] [40] 9 99 0.09 [0.04; 0.17]
Ladhani SN [Residents] [34] 46 105 0.44 [0.34; 0.54]
Paleker M [41] 41 112 0.37 [0.28; 0.46]
van den Besselaar JH [Residents] [35] 7 113 0.06 [0.03; 0.12]
Graham N [42] 46 126 0.37 [0.28; 0.46]
Taylor J [Residents] [40] 7 127 0.06 [0.02; 0.11]
Pham QT [43] 89 208 0.43 [0.36; 0.50]
Kennelly SP [Nursing home staff] [44] 97 395 0.25 [0.20; 0.29]
Lee JY [45] 80 694 0.12 [0.09; 0.14]
Kennelly SP [Nursing home residents] [44] 193 710 0.27 [0.24; 0.31]
Kasper MR [46] 572 1271 0.45 [0.42; 0.48]
Prediction interval . . [0.01; 0.81]
Screening: institutional setting
Tanacan A [47] 0 3 0.00 [0.00; 0.71]
Jeffery−Smith A [Staff] [48] 2 3 0.67 [0.09; 0.99]
Fisher MJ [49] 3 4 0.75 [0.19; 0.99]
Berghoff AS [50] 2 4 0.50 [0.07; 0.93]
Vohra [Undergoing chemotherapy] [51] 2 4 0.50 [0.07; 0.93]
Pamplona J [52] 2 5 0.40 [0.05; 0.85]
Viñuela MC [53] 8 8 1.00 [0.63; 1.00]
Bender WR [54] 6 8 0.75 [0.35; 0.97]
van Buul LW [Healthcare workers] [55] 0 9 0.00 [0.00; 0.34]
Vohra [Presurgical patients] [51] 10 10 1.00 [0.69; 1.00]
Varnell C [56] 5 10 0.50 [0.19; 0.81]
Haidar G [57] 9 11 0.82 [0.48; 0.98]
Kirshblum SC [58] 2 12 0.17 [0.02; 0.48]
Jeffery−Smith A [Residents] [48] 6 13 0.46 [0.19; 0.75]
Isoldi S [59] 4 15 0.27 [0.08; 0.55]
van Buul LW [Nursing home residents] [55] 3 16 0.19 [0.04; 0.46]
Wadhwa A [60] 6 17 0.35 [0.14; 0.62]
Balestrini S [61] 11 17 0.65 [0.38; 0.86]
Maki G [62] 16 18 0.89 [0.65; 0.99]
Bogani G [63] 10 19 0.53 [0.29; 0.76]
Green R [64] 22 22 1.00 [0.85; 1.00]
Martins Machado C [65] 5 22 0.23 [0.08; 0.45]
Khondaker T [66] 7 26 0.27 [0.12; 0.48]
Starling A [67] 29 31 0.94 [0.79; 0.99]
Rincon A [68] 9 35 0.26 [0.12; 0.43]
Pizarro−Sánchez MS [69] 5 38 0.13 [0.04; 0.28]
Patel MR [70] 43 55 0.78 [0.65; 0.88]
Aslam A [71] 38 65 0.58 [0.46; 0.71]
Marossy A [72] 46 67 0.69 [0.56; 0.79]
London V [73] 22 68 0.32 [0.22; 0.45]
Smith E [74] 42 103 0.41 [0.31; 0.51]
Weinbergerova B [75] 6 105 0.06 [0.02; 0.12]
Esteban I [76] 75 113 0.66 [0.57; 0.75]
Nunes MC [77] 14 115 0.12 [0.07; 0.20]
Hcini N [78] 87 137 0.64 [0.55; 0.72]
Shi SM [79] 21 146 0.14 [0.09; 0.21]
Andrikopoulou M [80] 46 158 0.29 [0.22; 0.37]
Blain H [81] 14 161 0.09 [0.05; 0.14]
Say D [82] 61 171 0.36 [0.29; 0.43]
Beiting KJ [83] 50 172 0.29 [0.22; 0.36]
Uçkay, I [84] 71 175 0.41 [0.33; 0.48]
Adhikari EH [85] 98 252 0.39 [0.33; 0.45]
Hussain A [86] 54 266 0.20 [0.16; 0.26]
Marcus JE [87] 199 403 0.49 [0.44; 0.54]
Ghinai I [88] 293 406 0.72 [0.68; 0.76]
White EM [89] 2194 5403 0.41 [0.39; 0.42]
Prediction interval . . [0.05; 0.93]
Screening: community setting
Hoehl S [90] 1 2 0.50 [0.01; 0.99]
Chang L [91] 2 4 0.50 [0.07; 0.93]
Rauch JN [92] 2 6 0.33 [0.04; 0.78]
AbdulRahman A [93] 3 6 0.50 [0.12; 0.88]
Theuring S [School students and staff] [94] 2 10 0.20 [0.03; 0.56]
Kutsuna S [95] 3 11 0.27 [0.06; 0.61]
Theuring S [Household members] [94] 0 14 0.00 [0.00; 0.23]
Hwang [96] 8 14 0.57 [0.29; 0.82]
Edelstein M [97] 4 20 0.20 [0.06; 0.44]
Migisha R [98 20 54 0.37 [0.24; 0.51]
Lavezzo E [99] 29 73 0.40 [0.28; 0.52]
Chamie G [100] 23 81 0.28 [0.19; 0.40]
Meyers KJ [101] 67 86 0.78 [0.68; 0.86]
Wi YM [102] 7 111 0.06 [0.03; 0.13]
Turunen T [103] 23 127 0.18 [0.12; 0.26]
Wong J [104] 16 138 0.12 [0.07; 0.18]
Eythorsson E [105] 25 178 0.14 [0.09; 0.20]
Al−Qahtani M [106] 116 188 0.62 [0.54; 0.69]
Cao S [107] 300 300 1.00 [0.99; 1.00]
Uysal E [108] 64 684 0.09 [0.07; 0.12]
Almazeedi S [109] 473 1096 0.43 [0.40; 0.46]
Malhotra S [110] 1272 1729 0.74 [0.71; 0.76]
Abraha HE [111] 1935 2617 0.74 [0.72; 0.76]
Ren R [112] 1612 3103 0.52 [0.50; 0.54]
Prediction interval . . [0.03; 0.92]
Screening: occupational
Lalani T [113] 4 8 0.50 [0.16; 0.84]
Ferreira VH [114] 5 9 0.56 [0.21; 0.86]
Morgan SC [115] 2 11 0.18 [0.02; 0.52]
Han X [116] 17 17 1.00 [0.80; 1.00]
Alshahrani MS [117] 12 18 0.67 [0.41; 0.87]
Stock AD [118] 6 19 0.32 [0.13; 0.57]
Fakhim H [119] 14 21 0.67 [0.43; 0.85]
Laws RL [120] 25 28 0.89 [0.72; 0.98]
Rivett L [121] 5 30 0.17 [0.06; 0.35]
Malagón−Rojas J [122] 11 35 0.31 [0.17; 0.49]
Hogan CA [123] 20 38 0.53 [0.36; 0.69]
Treibel TA [124] 12 44 0.27 [0.15; 0.43]
Tan−Loh J [125] 6 46 0.13 [0.05; 0.26]
Letizia AG [126] 46 51 0.90 [0.79; 0.97]
Lombardi A [127] 17 139 0.12 [0.07; 0.19]
Cariani L [128] 32 182 0.18 [0.12; 0.24]
Mahajan NN [129] 58 467 0.12 [0.10; 0.16]
Hall VJ [130] 293 1704 0.17 [0.15; 0.19]
Prediction interval . . [0.03; 0.93]
Prediction interval [0.02; 0.90]
0 0.2 0.4 0.6 0.8 1

References

1. Hijnen D, Marzano AV, Eyerich K, GeurtsvanKessel C, Gimenez-Arnau AM, Joly P, et al. Sars-Cov-2 Transmission from Presymptomatic Meeting Attendee, Germany. Emerg Infect Dis. 2020;26(8):1935-7. Epub 2020/05/12. doi: https://doi.org/10.3201/eid2608.201235. PMID: 32392125; PubMed Central PMCID: 7392453.

2. Zhang W, Cheng W, Luo L, Ma Y, Xu C, Qin P, et al. Secondary Transmission of Coronavirus Disease from Presymptomatic Persons, China. Emerg Infect Dis. 2020;26(8):1924-6. Epub 2020/05/27. doi: https://doi.org/10.3201/eid2608.201142. PMID: 32453686; PubMed Central PMCID: 7392433.

3. Cheng HY, Jian SW, Liu DP, Ng TC, Huang WT, Lin HH, et al. Contact Tracing Assessment of Covid-19 Transmission Dynamics in Taiwan and Risk at Different Exposure Periods before and after Symptom Onset. JAMA Intern Med. 2020;180(9):1156-63. Epub 2020/05/02. doi: https://doi.org/10.1001/jamainternmed.2020.2020. PMID: 32356867; PubMed Central PMCID: 7195694.

4. Brandstetter S, Roth S, Harner S, Buntrock-Dopke H, Toncheva AA, Borchers N, et al. Symptoms and Immunoglobulin Development in Hospital Staff Exposed to a Sars-Cov-2 Outbreak. Pediatr Allergy Immunol. 2020;31(7):841-7. Epub 2020/05/16. doi: https://doi.org/10.1111/pai.13278. PMID: 32413201.

5. Yousaf AR, Duca LM, Chu V, Reses HE, Fajans M, Rabold EM, et al. A Prospective Cohort Study in Nonhospitalized Household Contacts with Severe Acute Respiratory Syndrome Coronavirus 2 Infection: Symptom Profiles and Symptom Change over Time. Clin Infect Dis. 2021;73(7):e1841-e9. Epub 2020/07/29. doi: https://doi.org/10.1093/cid/ciaa1072. PMID: 32719874; PubMed Central PMCID: 7454397.

6. Wu J, Huang Y, Tu C, Bi C, Chen Z, Luo L, et al. Household Transmission of Sars-Cov-2, Zhuhai, China, 2020. Clin Infect Dis. 2020;71(16):2099-108. Epub 2020/05/12. doi: https://doi.org/10.1093/cid/ciaa557. PMID: 32392331; PubMed Central PMCID: 7239243.

7. Xie W, Chen Z, Wang Q, Song M, Cao Y, Wang L, et al. Infection and Disease Spectrum in Individuals with Household Exposure to Sars-Cov-2: A Family Cluster Cohort Study. J Med Virol. 2021;93(5):3033-46. Epub 20210212. doi: https://doi.org/10.1002/jmv.26847. PMID: 33538342; PubMed Central PMCID: PMC8014049.

8. Gettings JR, Gold JAW, Kimball A, Forsberg K, Scott C, Uehara A, et al. Severe Acute Respiratory Syndrome Coronavirus 2 Transmission in a Georgia School District-United States, December 2020-January 2021. Clin Infect Dis. 2022;74(2):319-26. doi: https://doi.org/10.1093/cid/ciab332. PMID: 33864375; PubMed Central PMCID: PMC8083290.

9. Jones A, Fialkowski V, Prinzing L, Trites J, Kelso P, Levine M. Assessment of Day-7 Postexposure Testing of Asymptomatic Contacts of Covid-19 Patients to Evaluate Early Release from Quarantine - Vermont, May-November 2020. MMWR Morb Mortal Wkly Rep. 2021;70(1):12-3. Epub 2021/01/08. doi: http://dx.doi.org/10.15585/mmwr.mm7001a3. PMID: 33411700; PubMed Central PMCID: 7790157.

10. Grijalva CG, Rolfes MA, Zhu Y, McLean HQ, Hanson KE, Belongia EA, et al. Transmission of Sars-Cov-2 Infections in Households - Tennessee and Wisconsin, April-September 2020. MMWR Morb Mortal Wkly Rep. 2020;69(44):1631-4. Epub 2020/11/06. doi: http://dx.doi.org/10.15585/mmwr.mm6944e1. PMID: 33151916; PubMed Central PMCID: 7643897.

11. Luo L, Liu D, Liao X, Wu X, Jing Q, Zheng J, et al. Contact Settings and Risk for Transmission in 3410 Close Contacts of Patients with Covid-19 in Guangzhou, China : A Prospective Cohort Study. Ann Intern Med. 2020;173(11):879-87. Epub 2020/08/14. doi: https://doi.org/10.7326/m20-2671. PMID: 32790510; PubMed Central PMCID: 7506769.

12. Shi Q, Hu Y, Peng B, Tang XJ, Wang W, Su K, et al. Effective Control of Sars-Cov-2 Transmission in Wanzhou, China. Nat Med. 2021;27(1):86-93. Epub 20201130. doi: https://doi.org/10.1038/s41591-020-01178-5. PMID: 33257893.

13. Hurst JH, Heston SM, Chambers HN, Cunningham HM, Price MJ, Suarez L, et al. Severe Acute Respiratory Syndrome Coronavirus 2 Infections among Children in the Biospecimens from Respiratory Virus-Exposed Kids (Brave Kids) Study. Clinical Infectious Diseases. 2021;73(9):e2875-e82. doi: https://doi.org/10.1093/cid/ciaa1693. PMID.

14. Pirnay JP, Selhorst P, Cochez C, Petrillo M, Claes V, Van der Beken Y, et al. Study of a Sars-Cov-2 Outbreak in a Belgian Military Education and Training Center in Maradi, Niger. Viruses. 2020;12(9). Epub 2020/09/02. doi: https://doi.org/10.3390/v12090949. PMID: 32867108; PubMed Central PMCID: 7552053.

15. Cardillo L, de Martinis C, Viscardi M, Esposito C, Sannino E, Lucibelli G, et al. Sars-Cov-2 Quantitative Real Time Pcr and Viral Loads Analysis among Asymptomatic and Symptomatic Patients: An Observational Study on an Outbreak in Two Nursing Facilities in Campania Region (Southern Italy). Infect Agent Cancer. 2021;16(1):45. Epub 20210622. doi: https://doi.org/10.1186/s13027-021-00388-x. PMID: 34158108; PubMed Central PMCID: PMC8218569.

16. Garibaldi PMM, Ferreira NN, Moraes GR, Moura JC, Espósito DLA, Volpe GJ, et al. Efficacy of Covid-19 Outbreak Management in a Skilled Nursing Facility Based on Serial Testing for Early Detection and Control. Braz J Infect Dis. 2021;25(2):101570. Epub 20210323. doi: https://doi.org/10.1016/j.bjid.2021.101570. PMID: 33773990; PubMed Central PMCID: PMC7985963.

17. Corcorran MA, Olin S, Rani G, Nasenbeny K, Constantino-Shor C, Holmes C, et al. Prolonged Persistence of Pcr-Detectable Virus During an Outbreak of Sars-Cov-2 in an Inpatient Geriatric Psychiatry Unit in King County, Washington. Am J Infect Control. 2021;49(3):293-8. Epub 2020/08/23. doi: https://doi.org/10.1016/j.ajic.2020.08.025. PMID: 32827597; PubMed Central PMCID: 7438365.

18. Yang N, Shen Y, Shi C, Ma AHY, Zhang X, Jian X, et al. In-Flight Transmission Cluster of Covid-19: A Retrospective Case Series. Infect Dis (Lond). 2020;52(12):891-901. Epub 2020/08/01. doi: https://doi.org/10.1080/23744235.2020.1800814. PMID: 32735163.

19. Schwierzeck V, Konig JC, Kuhn J, Mellmann A, Correa-Martinez CL, Omran H, et al. First Reported Nosocomial Outbreak of Severe Acute Respiratory Syndrome Coronavirus 2 in a Pediatric Dialysis Unit. Clin Infect Dis. 2021;72(2):265-70. Epub 2021/01/28. doi: https://doi.org/10.1093/cid/ciaa491. PMID: 33501962; PubMed Central PMCID: 7197625.

20. Danis K, Epaulard O, Benet T, Gaymard A, Campoy S, Bothelo-Nevers E, et al. Cluster of Coronavirus Disease 2019 (Covid-19) in the French Alps, 2020. Clin Infect Dis. 2020. Epub 2020/04/12. doi: https://doi.org/10.1093/cid/ciaa424. PMID: 32277759; PubMed Central PMCID: 7184384.

21. Romao VC, Oliveira-Ramos F, Cruz-Machado AR, Martins P, Barreira S, Silva-Dinis J, et al. A Covid-19 Outbreak in a Rheumatology Department Upon the Early Days of the Pandemic. Front Med (Lausanne). 2020;7:576162. Epub 2020/10/27. doi:

https://doi.org/10.3389/fmed.2020.576162. PMID: 33102507; PubMed Central PMCID: 7546334.

22. Orsi A, Domnich A, Pace VD, Ricucci V, Caligiuri P, Bottiglieri L, et al. Outbreak of Sars-Cov-2 Lineage 20i/501y.V1 in a Nursing Home Underlines the Crucial Role of Vaccination in Both Residents and Staff. Vaccines. 2021;9(6):591. doi. PMID: https://doi.org/10.3390/vaccines9060591.

23. Bohmer MM, Buchholz U, Corman VM, Hoch M, Katz K, Marosevic DV, et al. Investigation of a Covid-19 Outbreak in Germany Resulting from a Single Travel-Associated Primary Case: A Case Series. Lancet Infect Dis. 2020;20(8):920-8. Epub 2020/05/19. doi: https://doi.org/10.1016/s1473-3099(20)30314-5. PMID: 32422201; PubMed Central PMCID: 7228725.

24. Dora AV, Winnett A, Jatt LP, Davar K, Watanabe M, Sohn L, et al. Universal and Serial Laboratory Testing for Sars-Cov-2 at a Long-Term Care Skilled Nursing Facility for Veterans - Los Angeles, California, 2020. MMWR Morb Mortal Wkly Rep. 2020;69(21):651-5. Epub 2020/05/29. doi: https://doi.org/10.15585/mmwr.mm6921e1. PMID: 32463809; PubMed Central PMCID: 7269604.

25. Yau K, Muller MP, Lin M, Siddiqui N, Neskovic S, Shokar G, et al. Covid-19 Outbreak in an Urban Hemodialysis Unit. Am J Kidney Dis. 2020;76(5):690-5 e1. Epub 2020/07/19. doi: https://doi.org/10.1053/j.ajkd.2020.07.001. PMID: 32681983; PubMed Central PMCID: 7362862.

26. Redditt V, Wright V, Rashid M, Male R, Bogoch I. Outbreak of Sars-Cov-2 Infection at a Large Refugee Shelter in Toronto, April 2020: A Clinical and Epidemiologic Descriptive Analysis. CMAJ Open. 2020;8(4):E819-E24. Epub 2020/12/10. doi: https://doi.org/10.9778/cmajo.20200165. PMID: 33293331; PubMed Central PMCID: 7743908.

27. Tian S, Wu M, Chang Z, Wang Y, Zhou G, Zhang W, et al. Epidemiological Investigation and Intergenerational Clinical Characteristics of 24 Coronavirus Disease Patients Associated with a Supermarket Cluster: A Retrospective Study. BMC Public Health. 2021;21(1):647. Epub 2021/04/03. doi: https://doi.org/10.1186/s12889-021-10713-z. PMID: 33794836; PubMed Central PMCID: 8016429.

28. Harada S, Uno S, Ando T, Iida M, Takano Y, Ishibashi Y, et al. Control of a Nosocomial Outbreak of Covid-19 in a University Hospital. Open Forum Infect Dis. 2020;7(12):ofaa512. Epub 2020/12/18. doi: https://doi.org/10.1093/ofid/ofaa512. PMID: 33330740; PubMed Central PMCID: 7665726.

29. Park JH, Jang JH, Lee K, Yoo SJ, Shin H. Covid-19 Outbreak and Presymptomatic Transmission in Pilgrim Travelers Who Returned to Korea from Israel. J Korean Med Sci. 2020;35(48):e424. Epub 2020/12/15. doi: https://doi.org/10.3346/jkms.2020.35.e424. PMID: 33316860; PubMed Central PMCID: 7735917.

30. Patel MC, Chaisson LH, Borgetti S, Burdsall D, Chugh RK, Hoff CR, et al. Asymptomatic Sars-Cov-2 Infection and Covid-19 Mortality During an Outbreak Investigation in a Skilled Nursing Facility. Clin Infect Dis. 2020;71(11):2920-6. Epub 2020/06/18. doi: https://doi.org/10.1093/cid/ciaa763. PMID: 32548628; PubMed Central PMCID: 7337684.

31. Kittang BR, Hofacker SV, Solheim SP, Kruger K, Loland KK, Jansen K. Outbreak of Covid-19 at Three Nursing Homes in Bergen. Tidsskr Nor Laegeforen. 2020;140(11). Epub 2020/08/21. doi: https://doi.org/10.4045/tidsskr.20.0405. PMID: 32815356.

32. Pavli A, Smeti P, Papadima K, Andreopoulou A, Hadjianastasiou S, Triantafillou E, et al. A Cluster of Covid-19 in Pilgrims to Israel. J Travel Med. 2020;27(5). Epub 2020/06/26. doi: https://doi.org/10.1093/jtm/taaa102. PMID: 32584403; PubMed Central PMCID: 7337872.

33. Arons MM, Hatfield KM, Reddy SC, Kimball A, James A, Jacobs JR, et al. Presymptomatic Sars-Cov-2 Infections and Transmission in a Skilled Nursing Facility. N Engl J Med. 2020;382(22):2081-90. Epub 2020/04/25. doi: https://doi.org/10.1056/NEJMoa2008457. PMID: 32329971; PubMed Central PMCID: 7200056.

34. Ladhani SN, Jeffery-Smith A, Patel M, Janarthanan R, Fok J, Crawley-Boevey E, et al. High Prevalence of Sars-Cov-2 Antibodies in Care Homes Affected by Covid-19: Prospective Cohort Study, England. EClinicalMedicine. 2020;28:100597. Epub 2020/11/12. doi: https://doi.org/10.1016/j.eclinm.2020.100597. PMID: 33173854; PubMed Central PMCID: 7644437.

35. van den Besselaar JH, Sikkema RS, Koene F, van Buul LW, Oude Munnink BB, Frenay I, et al. Are Presymptomatic Sars-Cov-2 Infections in Nursing Home Residents Unrecognised Symptomatic Infections? Sequence and Metadata from Weekly Testing in an Extensive Nursing Home Outbreak. Age Ageing. 2021;50(5):1454-63. Epub 2021/05/09. doi: https://doi.org/10.1093/ageing/afab081. PMID: 33963830; PubMed Central PMCID: 8136016.

36. Schmitt J, Genet E, Danguy Des Deserts M, Chauvet-Atin S, Cungi PJ, Aries P, et al. Sars Cov2 Outbreak Management on a Landing Helicopter Dock: An Observational Retrospective Study. Infect Dis Now. 2021;51(5):424-8. Epub 20210619. doi: https://doi.org/10.1016/j.idnow.2021.06.303. PMID: 34157429; PubMed Central PMCID: PMC8214318.

37. Plucinski MM, Wallace M, Uehara A, Kurbatova EV, Tobolowsky FA, Schneider ZD, et al. Coronavirus Disease 2019 (Covid-19) in Americans Aboard the Diamond Princess Cruise Ship. Clin Infect Dis. 2021;72(10):e448-e57. Epub 2020/08/14. doi: https://doi.org/10.1093/cid/ciaa1180. PMID: 32785683; PubMed Central PMCID: 7454359.

38. Njuguna H, Wallace M, Simonson S, Tobolowsky FA, James AE, Bordelon K, et al. Serial Laboratory Testing for Sars-Cov-2 Infection among Incarcerated and Detained Persons in a Correctional and Detention Facility - Louisiana, April-May 2020. MMWR Morb Mortal Wkly Rep. 2020;69(26):836-40. Epub 2020/07/03. doi: https://doi.org/10.15585/mmwr.mm6926e2. PMID: 32614816; PubMed Central PMCID: 7332096.

39. Park SY, Kim YM, Yi S, Lee S, Na BJ, Kim CB, et al. Coronavirus Disease Outbreak in Call Center, South Korea. Emerg Infect Dis. 2020;26(8):1666-70. Epub 2020/04/24. doi: https://doi.org/10.3201/eid2608.201274. PMID: 32324530; PubMed Central PMCID: 7392450.

40. Taylor J, Carter RJ, Lehnertz N, Kazazian L, Sullivan M, Wang X, et al. Serial Testing for Sars-Cov-2 and Virus Whole Genome Sequencing Inform Infection Risk at Two Skilled Nursing Facilities with Covid-19 Outbreaks - Minnesota, April-June 2020. MMWR Morb Mortal Wkly Rep. 2020;69(37):1288-95. Epub 2020/09/24. doi: https://doi.org/10.15585/mmwr.mm6937a3. PMID: 32966272; PubMed Central PMCID: 7498172.

41. Paleker M, Tembo YA, Davies MA, Mahomed H, Pienaar D, Madhi SA, et al. Asymptomatic Covid-19 in South Africa - Implications for the Control of Transmission. Public

Health Action. 2021;11(2):58-60. doi: https://doi.org/10.5588/pha.20.0069. PMID: 34159063; PubMed Central PMCID: PMC8202632.

42. Graham NSN, Junghans C, Downes R, Sendall C, Lai H, McKirdy A, et al. Sars-Cov-2 Infection, Clinical Features and Outcome of Covid-19 in United Kingdom Nursing Homes. J Infect. 2020;81(3):411-9. Epub 2020/06/07. doi: https://doi.org/10.1016/j.jinf.2020.05.073. PMID: 32504743; PubMed Central PMCID: 7836316.

43. Pham QT, Rabaa MA, Luong DH, Tan DQ, Quang TD, Quach H-L, et al. The First 100 Days of Severe Acute Respiratory Syndrome Coronavirus 2 (Sars-Cov-2) Control in Vietnam. Clinical Infectious Diseases. 2020;72(9):e334-e42. doi: https://doi.org/10.1093/cid/ciaa1130. PMID: 32738143.

44. Kennelly SP, Dyer AH, Noonan C, Martin R, Kennelly SM, Martin A, et al. Asymptomatic Carriage Rates and Case Fatality of Sars-Cov-2 Infection in Residents and Staff in Irish Nursing Homes. Age Ageing. 2021;50(1):49-54. Epub 2020/09/29. doi: https://doi.org/10.1093/ageing/afaa220. PMID: 32986806; PubMed Central PMCID: 7543256.

45. Lee JY, Hong SW, Hyun M, Park JS, Lee JH, Suh YS, et al. Epidemiological and Clinical Characteristics of Coronavirus Disease 2019 in Daegu, South Korea. Int J Infect Dis. 2020;98:462-6. Epub 2020/07/24. doi: https://doi.org/10.1016/j.ijid.2020.07.017. PMID: 32702415; PubMed Central PMCID: 7371586.

46. Kasper MR, Geibe JR, Sears CL, Riegodedios AJ, Luse T, Von Thun AM, et al. An Outbreak of Covid-19 on an Aircraft Carrier. N Engl J Med. 2020;383(25):2417-26. Epub 2020/11/12. doi: https://doi.org/10.1056/nejmoa2019375. PMID: 33176077; PubMed Central PMCID: 7675688.

47. Tanacan A, Erol SA, Turgay B, Anuk AT, Secen EI, Yegin GF, et al. The Rate of Sars-Cov-2 Positivity in Asymptomatic Pregnant Women Admitted to Hospital for Delivery: Experience of a Pandemic Center in Turkey. Eur J Obstet Gynecol Reprod Biol. 2020;253:31-4. Epub 2020/08/09. doi: https://doi.org/10.1016/j.ejogrb.2020.07.051. PMID: 32763728; PubMed Central PMCID: 7390745.

48. Jeffery-Smith A, Dun-Campbell K, Janarthanan R, Fok J, Crawley-Boevey E, Vusirikala A, et al. Infection and Transmission of Sars-Cov-2 in London Care Homes Reporting No Cases or Outbreaks of Covid-19: Prospective Observational Cohort Study, England 2020. Lancet Reg Health Eur. 2021;3:100038. Epub 20210122. doi: https://doi.org/10.1016/j.lanepe.2021.100038. PMID: 33870248; PubMed Central PMCID: PMC7826003.

49. Fisher MJ, Psevdos G. Asymptomatic Sars-Cov-2 Infection Following First Dose Mrna-1273 Covid-19 Vaccine in a Veterans Affairs Long Term Care Facility. Am J Infect Control. 2021;49(9):1210-1. Epub 20210625. doi: https://doi.org/10.1016/j.ajic.2021.06.010. PMID: 34175364; PubMed Central PMCID: PMC8225993.

50. Berghoff AS, Gansterer M, Bathke AC, Trutschnig W, Hungerlander P, Berger JM, et al. Sars-Cov-2 Testing in Patients with Cancer Treated at a Tertiary Care Hospital During the Covid-19 Pandemic. J Clin Oncol. 2020;38(30):3547-54. Epub 2020/08/17. doi: https://doi.org/10.1200/jco.20.01442. PMID: 32795227; PubMed Central PMCID: 7571795.

51. Vohra LM, Jabeen D, Asif N, Ahad A. Covid-19 Pandemic and Breast Cancer Management: A Retrospective Observational Clinical Study from Pakistan. Ann Med Surg (Lond). 2021;63:102151. Epub 20210204. doi: https://doi.org/10.1016/j.amsu.2021.01.099. PMID: 33564461; PubMed Central PMCID: PMC7860941.

52. Pamplona J, Solano R, Ramírez M, Durandez R, Mohamed F, Pardo L, et al. High Prevalence of Sars-Cov-2 Infection in Patients Scheduled for Digestive Endoscopy after the Peak of the First Wave of the Pandemic. Gastroenterol Hepatol. 2021;44(9):614-9. Epub 20210420. doi: https://doi.org/10.1016/j.gastrohep.2021.03.001. PMID: 33862154; PubMed Central PMCID: PMC8056966.

53. Vinuela MC, De Leon-Luis JA, Alonso R, Catalan P, Lizarraga S, Munoz P, et al. Sars-Cov-2 Screening of Asymptomatic Women Admitted for Delivery Must Be Performed with a Combination of Microbiological Techniques: An Observational Study. Rev Esp Quimioter. 2020;33(6):415-21. Epub 2020/09/19. doi: https://doi.org/10.37201/req/088.2020. PMID: 32945157; PubMed Central PMCID: 7712338.

54. Bender WR, Hirshberg A, Coutifaris P, Acker AL, Srinivas SK. Universal Testing for Severe Acute Respiratory Syndrome Coronavirus 2 in 2 Philadelphia Hospitals: Carrier Prevalence and Symptom Development over 2 Weeks. Am J Obstet Gynecol MFM. 2020;2(4):100226. Epub 2020/09/22. doi: https://doi.org/10.1016/j.ajogmf.2020.100226. PMID: 32954248; PubMed Central PMCID: 7485442.

55. van Buul LW, van den Besselaar JH, Koene F, Buurman BM, Hertogh C, Group** C-N-S, et al. Asymptomatic Cases and Limited Transmission of Sars-Cov-2 in Residents and Healthcare Workers in Three Dutch Nursing Homes. Gerontol Geriatr Med. 2020;6:2333721420982800. Epub 2021/01/12. doi: https://doi.org/10.1177/2333721420982800. PMID: 33426178; PubMed Central PMCID: 7756037.

56. Varnell C, Jr., Harshman LA, Smith L, Liu C, Chen S, Al-Akash S, et al. Covid-19 in Pediatric Kidney Transplantation: The Improving Renal Outcomes Collaborative. Am J Transplant. 2021;21(8):2740-8. Epub 2021/01/17. doi: https://doi.org/10.1111/ajt.16501. PMID: 33452854; PubMed Central PMCID: 8013281.

57. Haidar G, Ayres A, King WC, McDonald M, Wells A, Mitchell SL, et al. Preprocedural Sars-Cov-2 Testing to Sustain Medically Needed Health Care Delivery During the Covid-19 Pandemic: A Prospective Observational Study. Open Forum Infectious Diseases. 2021;8(2). doi: https://doi.org/10.1093/ofid/ofab022. PMID.

58. Kirshblum SC, DeLauter G, Lopreiato MC, Pomeranz B, Dawson A, Hammerman S, et al. Screening Testing for Sars-Cov-2 Upon Admission to Rehabilitation Hospitals in a High Covid-19 Prevalence Community. PM R. 2020;12(10):1009-14. Epub 2020/07/24. doi: https://doi.org/10.1002/pmrj.12454. PMID: 32700434; PubMed Central PMCID: 7404891.

59. Isoldi S, Mallardo S, Marcellino A, Bloise S, Dilillo A, Iorfida D, et al. The Comprehensive Clinic, Laboratory, and Instrumental Evaluation of Children with Covid-19: A 6-Months Prospective Study. J Med Virol. 2021;93(5):3122-32. Epub 20210216. doi: https://doi.org/10.1002/jmv.26871. PMID: 33570199; PubMed Central PMCID: PMC8014060.

60. Wadhwa A, Fisher KA, Silver R, Koh M, Arons MM, Miller DA, et al. Identification of Presymptomatic and Asymptomatic Cases Using Cohort-Based Testing Approaches at a Large Correctional Facility-Chicago, Illinois, USA, May 2020. Clin Infect Dis. 2021;72(5):e128-e35. Epub 2020/12/04. doi: https://doi.org/10.1093/cid/ciaa1802. PMID: 33270101; PubMed Central PMCID: 7799274.

61. Balestrini S, Koepp MJ, Gandhi S, Rickman HM, Shin GY, Houlihan CF, et al. Clinical Outcomes of Covid-19 in Long-Term Care Facilities for People with Epilepsy. Epilepsy Behav. 2021;115:107602. Epub 2020/12/07. doi: https://doi.org/10.1016/j.yebeh.2020.107602. PMID: 33279440; PubMed Central PMCID: 7643621.

62. Maki G, Abdollah F, Dabaja A, Suleyman G. 462. Prevalence and Outcome of Asymptomatic Procedural Patients with Covid-19 Infection. Open Forum Infectious Diseases. 2020;7(Supplement_1):S298-S. doi: https://doi.org/10.1093/ofid/ofaa439.655. PMID.

63. Bogani G, Ditto A, Bosio S, Brusadelli C, Raspagliesi F. Cancer Patients Affected by Covid-19: Experience from Milan, Lombardy. Gynecol Oncol. 2020;158(2):262-5. Epub 2020/06/15. doi: https://doi.org/10.1016/j.ygyno.2020.06.161. PMID: 32534808; PubMed Central PMCID: 7286266.

64. Green R, Tulloch JSP, Tunnah C, Coffey E, Lawrenson K, Fox A, et al. Covid-19 Testing in Outbreak-Free Care Homes: What Are the Public Health Benefits? J Hosp Infect. 2021;111:89-95. Epub 2021/01/17. doi: https://doi.org/10.1016/j.jhin.2020.12.024. PMID: 33453349; PubMed Central PMCID: 7837210.

65. Machado CM, Kerbauy MN, Colturato I, Arcuri LJ, dos Santos ACF, Silva FR, et al. Clinical Characteristics and Outcomes of Covid-19 in Hsct Recipients. Blood. 2020;136:19-. Epub 2021/08/03. doi: https://doi.org/10.1182/blood-2020-141959. PMID: PMC8330389.

66. Khondaker T, Qader MA, Gosh K, Chowdhury GN, Ferdous T, Afroz S, et al. Clinical Profile and Outcome of Covid -19 in Children with Pre-Existing Renal Disease. Journal of Pediatric Nephrology. 2020;9(1):1-6. doi: https://doi.org/10.22037/jpn.v9i1.33008. PMID.

67. Starling A, White E, Showell D, Wyllie D, Kapadia S, Balakrishnan R. Whole Care Home Testing for Covid-19 in a Local Authority Area in the United Kingdom. medRxiv [Preprint]. 2020. doi: https://doi.org/10.1101/2020.08.06.20162859. PMID.

68. Rincón A, Moreso F, López-Herradón A, Fernández-Robres MA, Cidraque I, Nin J, et al. The Keys to Control a Covid-19 Outbreak in a Haemodialysis Unit. Clinical Kidney Journal. 2020;13(4):542-9. doi: https://doi.org/10.1093/ckj/sfaa119. PMID.

69. Pizarro-Sanchez MS, Avello A, Mas-Fontao S, Stock da Cunha T, Goma-Garces E, Pereira M, et al. Clinical Features of Asymptomatic Sars-Cov-2 Infection in Hemodialysis Patients. Kidney Blood Press Res. 2021;46(1):126-34. Epub 2021/01/28. doi: https://doi.org/10.1159/000512535. PMID: 33503627; PubMed Central PMCID: 7900451.

70. Patel MR, Kushwaha RS, Behera M, Bhadauria DS, Yachha M, Kaul A, et al. Aftermath of Fortnightly Universal Testing for Severe Acute Respiratory Corona Virus-2 Infection in Maintenance Hemodialysis Patients. Semin Dial. 2021;34(5):338-46. Epub 20210624. doi: https://doi.org/10.1111/sdi.12997. PMID: 34169574; PubMed Central PMCID: PMC8447208.

71. Aslam A, Singh J, Robilotti E, Chow K, Bist T, Reidy-Lagunes D, et al. Severe Acute Respiratory Syndrome Coronavirus 2 Surveillance and Exposure in the Perioperative Setting with Universal Testing and Personal Protective Equipment Policies. Clin Infect Dis. 2021;73(9):e3013-e8. Epub 2020/10/23. doi: https://doi.org/10.1093/cid/ciaa1607. PMID: 33090210; PubMed Central PMCID: 7665395.

72. Marossy A, Rakowicz S, Bhan A, Noon S, Rees A, Virk M, et al. A Study of Universal Severe Acute Respiratory Syndrome Coronavirus 2 Rna Testing among Residents and Staff in a Large Group of Care Homes in South London. J Infect Dis. 2021;223(3):381-8. Epub 2020/09/06. doi: https://doi.org/10.1093/infdis/jiaa565. PMID: 32889532; PubMed Central PMCID: 7499645.

73. London V, McLaren R, Jr., Atallah F, Cepeda C, McCalla S, Fisher N, et al. The Relationship between Status at Presentation and Outcomes among Pregnant Women with Covid-19. Am J Perinatol. 2020;37(10):991-4. Epub 2020/05/20. doi:

https://doi.org/10.1055/s-0040-1712164. PMID: 32428964; PubMed Central PMCID: 7416203.

74. Smith E, Aldus CF, Brainard J, Dunham S, Hunter PR, Steel N, et al. Testing for Sars-Cov-2 in Care Home Staff and Residents in English Care Homes: A Service Evaluation. medRxiv. 2020:2020.08.04.20165928. doi: https://doi.org/10.1101/2020.08.04.20165928. PMID.

75. Weinbergerova B, Mayer J, Hrabovsky S, Novakova Z, Pospisil Z, Martykanova L, et al. Covid-19's Natural Course among Ambulatory Monitored Outpatients. Sci Rep. 2021;11(1):10124. Epub 20210512. doi: https://doi.org/10.1038/s41598-021-89545-1. PMID: 33980931; PubMed Central PMCID: PMC8115337.

76. Esteban I, Bergero G, Alves C, Bronstein M, Ziegler V, Wood C, et al. Asymptomatic Covid-19 in the Elderly: Dementia and Viral Clearance as Risk Factors for Disease Progression. TP92 TP092 CLINICAL ADVANCES IN SARS-COV-2 AND COVID-19. 2021:A3826-A. doi: https://doi.org/10.1164/ajrccm-conference.2021.203.1_MeetingAbstracts.A3826. PMID.

77. Nunes MC, Baillie VL, Kwatra G, Bhikha S, Verwey C, Menezes C, et al. Severe Acute Respiratory Syndrome Coronavirus 2 Infection among Healthcare Workers in South Africa: A Longitudinal Cohort Study. Clin Infect Dis. 2021;73(10):1896-900. doi: https://doi.org/10.1093/cid/ciab398. PMID: 33949670; PubMed Central PMCID: PMC8135922.

78. Hcini N, Maamri F, Picone O, Carod JF, Lambert V, Mathieu M, et al. Maternal, Fetal and Neonatal Outcomes of Large Series of Sars-Cov-2 Positive Pregnancies in Peripartum Period: A Single-Center Prospective Comparative Study. Eur J Obstet Gynecol Reprod Biol. 2021;257:11-8. Epub 2020/12/15. doi: https://doi.org/10.1016/j.ejogrb.2020.11.068. PMID: 33310656; PubMed Central PMCID: 7705341.

79. Shi SM, Bakaev I, Chen H, Travison TG, Berry SD. Risk Factors, Presentation, and Course of Coronavirus Disease 2019 in a Large, Academic Long-Term Care Facility. J Am Med Dir Assoc. 2020;21(10):1378-83.e1. Epub 20200825. doi: https://doi.org/10.1016/j.jamda.2020.08.027. PMID: 32981664; PubMed Central PMCID: 7447263.

80. Andrikopoulou M, Madden N, Wen T, Aubey JJ, Aziz A, Baptiste CD, et al. Symptoms and Critical Illness among Obstetric Patients with Coronavirus Disease 2019 (Covid-19) Infection. Obstet Gynecol. 2020;136(2):291-9. Epub 2020/05/28. doi: https://doi.org/10.1097/aog.0000000000003996. PMID: 32459701.

81. Blain H, Gamon L, Tuaillon E, Pisoni A, Giacosa N, Albrand M, et al. Atypical Symptoms, Sars-Cov-2 Test Results and Immunisation Rates in 456 Residents from Eight Nursing Homes Facing a Covid-19 Outbreak. Age Ageing. 2021;50(3):641-8. doi: https://doi.org/10.1093/ageing/afab050. PMID: 33620381; PubMed Central PMCID: PMC7929417.

82. Say D, Crawford N, McNab S, Wurzel D, Steer A, Tosif S. Post-Acute Covid-19 Outcomes in Children with Mild and Asymptomatic Disease. Lancet Child Adolesc Health. 2021;5(6):e22-e3. Epub 20210421. doi: https://doi.org/10.1016/s2352-4642(21)00124-3. PMID: 33891880; PubMed Central PMCID: PMC8057863.

83. Beiting KJ, Huisingh-Scheetz M, Walker J, Graupner J, Martinchek M, Thompson K, et al. Management and Outcomes of a Covid-19 Outbreak in a Nursing Home with

Predominantly Black Residents. J Am Geriatr Soc. 2021;69(5):1155-65. Epub 20210324. doi: https://doi.org/10.1111/jgs.17126. PMID: 33739444; PubMed Central PMCID: PMC8218575.

84. Uçkay I, Steinwender L, Burkhard J, Holy D, Strähl M, Farshad M. Outcomes of Asymptomatic Hospital Employees in Covid-19 Post-Exposure Quarantine During the Second Pandemic Wave in Zurich. J Hosp Infect. 2021;113:189-91. Epub 20210421. doi: https://doi.org/10.1016/j.jhin.2021.04.010. PMID: 33891983; PubMed Central PMCID: PMC8057933.

85. Adhikari EH, Moreno W, Zofkie AC, MacDonald L, McIntire DD, Collins RRJ, et al. Pregnancy Outcomes among Women with and without Severe Acute Respiratory Syndrome Coronavirus 2 Infection. JAMA Netw Open. 2020;3(11):e2029256. Epub 2020/11/20. doi: https://doi.org/10.1001/jamanetworkopen.2020.29256. PMID: 33211113; PubMed Central PMCID: 7677755.

86. Hussain A, Satti L, Hanif F, Shoaib M, Ghauri MA, Khan Niazi GA, et al. Clinical and Virological Course of Sars-Cov 2 Infected Patients in a Tertiary Care Hospital in Pakistan. J Ayub Med Coll Abbottabad. 2020;32(Suppl 1)(4):S602-s6. doi: https://pubmed.ncbi.nlm.nih.gov/33754515/. PMID: 33754515; PubMed Central PMCID: 33754515.

87. Marcus JE, Frankel DN, Pawlak MT, Casey TM, Cybulski RJ, Jr., Enriquez E, et al. Risk Factors Associated with Covid-19 Transmission among Us Air Force Trainees in a Congregant Setting. JAMA Netw Open. 2021;4(2):e210202. Epub 20210201. doi: https://doi.org/10.1001/jamanetworkopen.2021.0202. PMID: 33630090; PubMed Central PMCID: PMC7907953.

88. Ghinai I, Davis ES, Mayer S, Toews KA, Huggett TD, Snow-Hill N, et al. Risk Factors for Severe Acute Respiratory Syndrome Coronavirus 2 Infection in Homeless Shelters in Chicago, Illinois-March-May, 2020. Open Forum Infect Dis. 2020;7(11):ofaa477. Epub 2020/12/03. doi: https://doi.org/10.1093/ofid/ofaa477. PMID: 33263069; PubMed Central PMCID: 7665740.

89. White EM, Santostefano CM, Feifer RA, Kosar CM, Blackman C, Gravenstein S, et al. Asymptomatic and Presymptomatic Severe Acute Respiratory Syndrome Coronavirus 2 Infection Rates in a Multistate Sample of Skilled Nursing Facilities. JAMA Intern Med. 2020;180(12):1709-11. Epub 2020/10/20. doi: https://doi.org/10.1001/jamainternmed.2020.5664. PMID: 33074318; PubMed Central PMCID: 7573793.

90. Hoehl S, Rabenau H, Berger A, Kortenbusch M, Cinatl J, Bojkova D, et al. Evidence of Sars-Cov-2 Infection in Returning Travelers from Wuhan, China. N Engl J Med. 2020;382(13):1278-80. Epub 2020/02/19. doi: https://doi.org/10.1056/nejmc2001899. PMID: 32069388; PubMed Central PMCID: 7121749.

91. Chang L, Zhao L, Gong H, Wang L, Wang L. Severe Acute Respiratory Syndrome Coronavirus 2 Rna Detected in Blood Donations. Emerg Infect Dis. 2020;26(7):1631-3. Epub 2020/04/04. doi: https://doi.org/10.3201/eid2607.200839. PMID: 32243255; PubMed Central PMCID: 7323524.

92. Rauch JN, Valois E, Ponce-Rojas JC, Aralis Z, Lach RS, Zappa F, et al. Comparison of Severe Acute Respiratory Syndrome Coronavirus 2 Screening Using Reverse Transcriptase-Quantitative Polymerase Chain Reaction or Crispr-Based Assays in Asymptomatic College Students. JAMA Netw Open. 2021;4(2):e2037129. Epub 2021/02/12. doi: https://doi.org/10.1001/jamanetworkopen.2020.37129. PMID: 33570576; PubMed Central PMCID: 7879237.

93. AbdulRahman A, AlAli S, Yaghi O, Shabaan M, Otoom S, Atkin SL, et al. Covid-19 and Sickle Cell Disease in Bahrain. Int J Infect Dis. 2020;101:14-6. Epub 2020/09/28. doi: https://doi.org/10.1016/j.ijid.2020.09.1433. PMID: 32980556; PubMed Central PMCID: 7833176.

94. Theuring S, Thielecke M, van Loon W, Hommes F, Hulso C, von der Haar A, et al. Sars-Cov-2 Infection and Transmission in School Settings During the Second Covid-19 Wave: A Cross-Sectional Study, Berlin, Germany, November 2020. Euro Surveill. 2021;26(34). Epub 2021/08/28. doi: https://doi.org/10.2807/1560-7917.es.2021.26.34.2100184. PMID: 34448448; PubMed Central PMCID: 8393892.

95. Kutsuna S, Suzuki T, Hayakawa K, Tsuzuki S, Asai Y, Suzuki T, et al. Sars-Cov-2 Screening Test for Japanese Returnees from Wuhan, China, January 2020. Open Forum Infect Dis. 2020;7(7):ofaa243. Epub 2020/08/06. doi: https://doi.org/10.1093/ofid/ofaa243. PMID: 32754627; PubMed Central PMCID: 7337761.

96. Hwang CE, Kussman A, Christle JW, Froelicher V, Wheeler MT, Moneghetti KJ. Findings from Cardiovascular Evaluation of National Collegiate Athletic Association Division I Collegiate Student-Athletes after Asymptomatic or Mildly Symptomatic Sars-Cov-2 Infection. Clin J Sport Med. 2022;32(2):103-7. doi: https://doi.org/10.1097/jsm.0000000000000954. PMID: 34173780.

97. Edelstein M, Obi C, Chand M, Hopkins S, Brown K, Ramsay M. Sars-Cov-2 Infection in London, England: Changes to Community Point Prevalence around Lockdown Time, March-May 2020. J Epidemiol Community Health. 2021;75(2):185-8. Epub 2020/10/03. doi: https://doi.org/10.1136/jech-2020-214730. PMID: 33004659; PubMed Central PMCID: 7815898.

98. Migisha R, Kwesiga B, Mirembe BB, Amanya G, Kabwama SN, Kadobera D, et al. Early Cases of Sars-Cov-2 Infection in Uganda: Epidemiology and Lessons Learned from Risk-Based Testing Approaches - March-April 2020. Global Health. 2020;16(1):114. Epub 2020/11/27. doi: https://doi.org/10.1186/s12992-020-00643-7. PMID: 33239041; PubMed Central PMCID: 7686950.

99. Lavezzo E, Franchin E, Ciavarella C, Cuomo-Dannenburg G, Barzon L, Del Vecchio C, et al. Suppression of a Sars-Cov-2 Outbreak in the Italian Municipality of Vo'. Nature. 2020;584(7821):425-9. Epub 2020/07/01. doi: https://doi.org/10.1038/s41586-020-2488-1. PMID: 32604404.

100. Chamie G, Marquez C, Crawford E, Peng J, Petersen M, Schwab D, et al. Community Transmission of Severe Acute Respiratory Syndrome Coronavirus 2 Disproportionately Affects the Latinx Population During Shelter-in-Place in San Francisco. Clin Infect Dis. 2021;73(Suppl 2):S127-S35. Epub 2020/08/22. doi: https://doi.org/10.1093/cid/ciaa1234. PMID: 32821935; PubMed Central PMCID: 7499499.

101. Meyers KJ, Dillman B, Williams C, Jiang J, Clifford N, Miller JL, et al. Follow-up of Sars-Cov-2 Positive Subgroup from the Asymptomatic Novel Coronavirus Infection Study. J Med Virol. 2021;93(5):2925-31. Epub 2021/01/20. doi: https://doi.org/10.1002/jmv.26810. PMID: 33463731; PubMed Central PMCID: 8014630.

102. Wi YM, Lim SJ, Kim SH, Lim S, Lee SJ, Ryu BH, et al. Response System for and Epidemiological Features of Covid-19 in Gyeongsangnam-Do Province in South Korea. Clin Infect Dis. 2021;72(4):661-7. Epub 2020/07/17. doi: https://doi.org/10.1093/cid/ciaa967. PMID: 32672789; PubMed Central PMCID: 7454481.

103. Turunen T, Kontunen K, Sugulle K, Hieta P, Snellman O, Hussein I, et al. Covid-19 Outbreak at a Reception Centre for Asylum Seekers in Espoo, Finland. J Migr Health. 2021;3:100043. Epub 20210416. doi: https://doi.org/10.1016/j.jmh.2021.100043. PMID: 33880457; PubMed Central PMCID: PMC8051004.

104. Wong J, Abdul Aziz ABZ, Chaw L, Mahamud A, Griffith MM, Ying-Ru LO, et al. High Proportion of Asymptomatic and Presymptomatic Covid-19 Infections in Travelers and Returning Residents to Brunei. J Travel Med. 2020. doi: https://doi.org/10.1093/jtm/taaa066. PMID.

105. Eythorsson E, Helgason D, Ingvarsson RF, Bjornsson HK, Olafsdottir LB, Bjarnadottir V, et al. Clinical Spectrum of Coronavirus Disease 2019 in Iceland: Population Based Cohort Study. BMJ. 2020;371:m4529. Epub 2020/12/04. doi: https://doi.org/10.1136/bmj.m4529. PMID: 33268329; PubMed Central PMCID: 7708618.

106. Al-Qahtani M, AlAli S, AbdulRahman A, Salman Alsayyad A, Otoom S, Atkin SL. The Prevalence of Asymptomatic and Symptomatic Covid-19 in a Cohort of Quarantined Subjects. Int J Infect Dis. 2021;102:285-8. Epub 2020/11/07. doi: https://doi.org/10.1016/j.ijid.2020.10.091. PMID: 33157290; PubMed Central PMCID: 7607262.

107. Cao S, Gan Y, Wang C, Bachmann M, Wei S, Gong J, et al. Post-Lockdown Sars-Cov-2 Nucleic Acid Screening in Nearly Ten Million Residents of Wuhan, China. Nat Commun. 2020;11(1):5917. Epub 2020/11/22. doi: https://doi.org/10.1038/s41467-020-19802-w. PMID: 33219229; PubMed Central PMCID: 7679396.

108. Uysal E, Kilinçer A, Cebeci H, Özer H, Demir NA, Öztürk M, et al. Chest Ct Findings in Rt-Pcr Positive Asymptomatic Covid-19 Patients. Clinical Imaging. 2021;77:37-42. doi: https://doi.org/10.1016/j.clinimag.2021.01.030. PMID.

109. Almazeedi S, Al-Youha S, Jamal MH, Al-Haddad M, Al-Muhaini A, Al-Ghimlas F, et al. Characteristics, Risk Factors and Outcomes among the First Consecutive 1096 Patients Diagnosed with Covid-19 in Kuwait. EClinicalMedicine. 2020;24:100448. Epub 2020/08/09. doi: https://doi.org/10.1016/j.eclinm.2020.100448. PMID: 32766546; PubMed Central PMCID: 7335246.

110. Malhotra S, Rahi M, Das P, Chaturvedi R, Chhibber-Goel J, Anvikar A, et al. Epidemiological Profiles and Associated Risk Factors of Sars-Cov-2 Positive Patients Based on a High-Throughput Testing Facility in India. Open Biol. 2021;11(6):200288. Epub 20210602. doi: https://doi.org/10.1098/rsob.200288. PMID: 34062097; PubMed Central PMCID: PMC8169211.

111. Abraha HE, Gessesse Z, Gebrecherkos T, Kebede Y, Weldegiargis AW, Tequare MH, et al. Clinical Features and Risk Factors Associated with Morbidity and Mortality among Patients with Covid-19 in Northern Ethiopia. Int J Infect Dis. 2021;105:776-83. Epub 20210316. doi: https://doi.org/10.1016/j.ijid.2021.03.037. PMID: 33741488; PubMed Central PMCID: PMC7962557.

112. Ren R, Zhang Y, Li Q, McGoogan JM, Feng Z, Gao GF, et al. Asymptomatic Sars-Cov-2 Infections among Persons Entering China from April 16 to October 12, 2020. Jama. 2021;325(5):489-92. doi: https://doi.org/10.1001/jama.2020.23942. PMID: 33528529; PubMed Central PMCID: PMC7856538.

113. Lalani T, Lee TK, Laing ED, Ritter A, Cooper E, Lee M, et al. Sars-Cov-2 Infections and Serologic Responses among Military Personnel Deployed on the Usns Comfort to New York City During the Covid-19 Pandemic. Open Forum Infect Dis. 2021;8(2):ofaa654. Epub

20210123. doi: https://doi.org/10.1093/ofid/ofaa654. PMID: 33553482; PubMed Central PMCID: PMC7856331.

114. Ferreira VH, Chruscinski A, Kulasingam V, Pugh TJ, Dus T, Wouters B, et al. Prospective Observational Study and Serosurvey of Sars-Cov-2 Infection in Asymptomatic Healthcare Workers at a Canadian Tertiary Care Center. PLoS One. 2021;16(2):e0247258. Epub 2021/02/17. doi: https://doi.org/10.1371/journal.pone.0247258. PMID: 33592074; PubMed Central PMCID: 7886177.

115. Morgan SC, Aigner S, Anderson C, Belda-Ferre P, De Hoff P, Marotz CA, et al. Automated, Miniaturized, and Scalable Screening of Healthcare Workers, First Responders, and Students for Sars-Cov-2 in San Diego County. medRxiv [Preprint]. 2021:2021.06.25.21257885. doi: https://doi.org/10.1101/2021.06.25.21257885. PMID.

116. Han X, Wei X, Alwalid O, Cao Y, Li Y, Wang L, et al. Severe Acute Respiratory Syndrome Coronavirus 2 among Asymptomatic Workers Screened for Work Resumption, China. Emerg Infect Dis. 2020;26(9). Epub 2020/06/20. doi: https://doi.org/10.3201/eid2609.201848. PMID: 32553070; PubMed Central PMCID: 7454110.

117. Alshahrani MS, Alnimr A, Alnassri S, Alfarag S, Aljehani Y, Alabdali M. Prevalence of the Sars-Cov-2 Infection among Post-Quarantine Healthcare Workers. J Multidiscip Healthc. 2020;13:1927-36. Epub 2020/12/29. doi: https://doi.org/10.2147/jmdh.s279469. PMID: 33363380; PubMed Central PMCID: 7752650.

118. Stock AD, Bader ER, Cezayirli P, Inocencio J, Chalmers SA, Yassari R, et al. Covid-19 Infection among Healthcare Workers: Serological Findings Supporting Routine Testing. Front Med (Lausanne). 2020;7:471. Epub 2020/09/26. doi: https://doi.org/10.3389/fmed.2020.00471. PMID: 32974370; PubMed Central PMCID: 7472984.

119. Fakhim H, Nasri E, Aboutalebian S, Gholipour S, Nikaeen M, Vaezi A, et al. Asymptomatic Carriers of Coronavirus Disease 2019 among Healthcare Workers in Isfahan, Iran. Future Virology. 2021;16(2):93-8. doi: https://dx.doi.org/10.2217%2Ffvl-2020-0224. PMID.

120. Laws RL, Biraro S, Kirungi W, Gianetti B, Aibo D, Awor AC, et al. Coronavirus Disease 2019 (Covid-19) Mitigation Efforts and Testing During an in-Person Training Event-Uganda, 12-29 October 2020. Clin Infect Dis. 2021;73(Suppl 1):S42-s4. doi: https://doi.org/10.1093/cid/ciab331. PMID: 33912911; PubMed Central PMCID: PMC8135603.

121. Rivett L, Sridhar S, Sparkes D, Routledge M, Jones NK, Forrest S, et al. Screening of Healthcare Workers for Sars-Cov-2 Highlights the Role of Asymptomatic Carriage in Covid-19 Transmission. Elife. 2020;9. Epub 2020/05/12. doi: https://doi.org/10.7554/elife.58728. PMID: 32392129.

122. Malagon-Rojas J, Gomez-Rendon C, Parra EL, Almentero J, Palma R, Lopez R, et al. Sars-Cov-2 and Rt-Pcr in Asymptomatic Patients: Results of a Cohort of Workers at El Dorado International Airport in Bogota, 2020. Biomedica. 2020;40(Supl. 2):166-72. Epub 2020/11/06. doi: https://doi.org/10.7705/biomedica.5802. PMID: 33152200; PubMed Central PMCID: 7676845.

123. Hogan CA, Gombar S, Wang H, Roltgen K, Shi RZ, Holubar M, et al. Large-Scale Testing of Asymptomatic Healthcare Personnel for Severe Acute Respiratory Syndrome Coronavirus 2. Emerg Infect Dis. 2021;27(1). Epub 2020/12/02. doi:

https://doi.org/10.3201/eid2701.203892. PMID: 33256889; PubMed Central PMCID: 7774535.

124. Treibel TA, Manisty C, Burton M, McKnight NA, Lambourne J, Augusto JB, et al. Covid-19: Pcr Screening of Asymptomatic Health-Care Workers at London Hospital. Lancet. 2020. doi: https://doi.org/10.1016/s0140-6736(20)31100-4. PMID.

125. Tan-Loh J, Cheong BMK. A Descriptive Analysis of Clinical Characteristics of Covid-19 among Healthcare Workers in a District Specialist Hospital. Med J Malaysia. 2021;76(1):24-8. Epub 2021/01/30. doi: https://pubmed.ncbi.nlm.nih.gov/33510104/. PMID: 33510104.

126. Letizia AG, Ramos I, Obla A, Goforth C, Weir DL, Ge Y, et al. Sars-Cov-2 Transmission among Marine Recruits During Quarantine. N Engl J Med. 2020;383(25):2407-16. Epub 2020/11/12. doi: https://doi.org/10.1056/nejmoa2029717. PMID: 33176093; PubMed Central PMCID: 7675690.

127. Lombardi A, Consonni D, Carugno M, Bozzi G, Mangioni D, Muscatello A, et al. Characteristics of 1573 Healthcare Workers Who Underwent Nasopharyngeal Swab Testing for Sars-Cov-2 in Milan, Lombardy, Italy. Clin Microbiol Infect. 2020;26(10):1413 e9- e13. Epub 2020/06/23. doi: https://doi.org/10.1016/j.cmi.2020.06.013. PMID: 32569835; PubMed Central PMCID: 7305713.

128. Cariani L, Orena BS, Ambrogi F, Gambazza S, Maraschini A, Dodaro A, et al. Time Length of Negativization and Cycle Threshold Values in 182 Healthcare Workers with Covid-19 in Milan, Italy: An Observational Cohort Study. Int J Environ Res Public Health. 2020;17(15). Epub 2020/07/29. doi: https://doi.org/10.3390/ijerph17155313. PMID: 32718008; PubMed Central PMCID: 7432921.

129. Mahajan NN, Kesarwani SN, Shinde SS, Nayak A, Modi DN, Mahale SD, et al. Co-Infection of Malaria and Dengue in Pregnant Women with Sars-Cov-2. Int J Gynaecol Obstet. 2020;151(3):459-62. Epub 2020/10/23. doi: https://doi.org/10.1002/ijgo.13415. PMID: 33090458; PubMed Central PMCID: 7611276.

130. Hall VJ, Foulkes S, Charlett A, Atti A, Monk EJM, Simmons R, et al. Sars-Cov-2 Infection Rates of Antibody-Positive Compared with Antibody-Negative Health-Care Workers in England: A Large, Multicentre, Prospective Cohort Study (Siren). Lancet. 2021;397(10283):1459-69. Epub 20210409. doi: https://doi.org/10.1016/s0140-6736(21)00675-9. PMID: 33844963; PubMed Central PMCID: PMC8040523.
