# Supplementary material for: Occurrence and transmission potential of asymptomatic and presymptomatic SARS-CoV-2 infections: Update of a living systematic review and meta-analysis
Source: PLoS Med. 2022 May 26;19(5):e1003987. doi: 10.1371/journal.pmed.1003987 (PMC9135333; doi:10.1371/journal.pmed.1003987)
Supplement: S3 Fig — (PDF) [file pmed.1003987.s013.pdf]

**S3 Fig. Risk of bias assessment of studies in review question 1 and 2**

**Question 1**

Contact investigation

Risk of bias items

| Study | 1 | 2 | 3 | 4 | 5 | 6 |
|---|---|---|---|---|---|---|
| Cheng HY [1] | + | + | X | - | - | + |
| Wu J [2] | X | - | + | - | + | - |
| Hijnen D [3] | + | - | - | - | - | + |
| Brandstetter S [4] | - | - | + | - | + | + |
| Zhang W [5] | + | + | - | - | + | + |
| Yousaf AR [6] | + | - | + | + | + | + |
| Luo L2 [7] | + | + | + | + | + | + |
| Hurst JH [8] | X | + | + | X | X | X |
| Grijalva CG [9] | - | - | - | + | X | + |
| Shi Q [10] | + | + | - | - | + | + |
| Jones A [11] | - | - | - | - | + | + |
| Xie W [12] | + | + | + | - | + | + |
| Gettings JR [13] | - | - | + | + | X | X |

Outbreak investigation

Risk of bias items

| Study | 1 | 2 | 3 | 4 | 5 | 6 |
|---|---|---|---|---|---|---|
| Danis K [14] | - | - | + | + | + | + |
| Park SY [15] | + | + | - | - | + | + |
| Arons MM [16] | + | + | + | + | + | + |
| Schwierzeck V [17] | + | + | - | + | + | + |
| Böhmer MM [18] | + | + | + | - | + | + |
| Dora AV [19] | + | + | X | + | + | + |
| Graham N [20] | + | + | X | X | X | + |
| Patel MC [21] | + | + | + | + | + | + |
| Pavli A [22] | + | + | + | - | + | + |
| Njuguna H [23] | - | + | X | X | + | + |
| Yau K [24] | + | + | - | - | + | + |
| Lee JY [25] | - | - | X | + | X | + |
| Yang N [26] | + | + | + | + | + | + |
| Pham QT [27] | + | + | - | - | + | + |
| Plucinski MM [28] | X | X | + | X | X | + |
| Kittang BR [29] | + | - | + | - | + | + |
| Corcorran MA [30] | + | + | + | + | + | + |
| Pirnay JP [31] | + | + | + | - | + | + |
| Taylor J [32] | - | - | - | - | + | + |
| Kennelly SP [33] | + | - | X | - | X | + |
| Romao VC [34] | + | - | + | + | + | + |
| Ladhani SN [35] | + | + | - | - | + | + |
| Kasper MR [36] | + | + | + | + | + | + |
| Redditt V [37] | + | - | + | X | - | + |
| Park JH [38] | + | + | X | - | + | + |
| Harada S [39] | + | - | + | + | + | + |
| Garibaldi PMM [40] | + | + | - | + | X | + |
| Tian S [41] | + | + | + | - | + | + |
| van den Besselaar JH [42] | + | X | + | - | + | X |
| Orsi A [43] | + | + | - | - | + | + |
| Schmitt J [44] | + | + | - | + | - | + |
| Paleker M [45] | - | - | - | - | + | X |
| Cardillo L [46] | - | - | X | - | + | + |

Screening: community setting

| Study | Risk of bias items: 1 | 2 | 3 | 4 | 5 | 6 |
|---|---|---|---|---|---|---|
| Chang L [47] | X | + | - | - | + | + |
| Lavezzo E [48] | + | - | + | X | + | + |
| Hoehl S [49] | + | + | + | + | + | + |
| Wong J [50] | + | - | - | - | + | + |
| Almazeedi S [51] | + | - | + | - | + | + |
| Wi YM [52] | + | + | + | + | + | + |
| Kutsuna S [53] | + | - | - | + | + | + |
| Eythorsson E [54] | + | - | + | + | X | + |
| Chamie G [55] | X | - | - | - | + | X |
| AbdulRahman A [56] | X | - | - | - | + | + |
| Edelstein M [57] | X | - | X | X | + | + |
| Al-Qahtani M [58] | + | + | X | + | + | + |
| Cao S [59] | + | - | X | - | + | + |
| Migisha R [60] | - | - | + | + | - | + |
| Meyers KJ [61] | X | - | + | X | + | + |
| Ren R [62] | + | + | - | + | + | + |
| Rauch JN [63] | X | - | - | - | X | + |
| Uysal E [64] | + | + | + | - | + | + |
| Abraha HE [65] | + | + | + | + | + | + |
| Turunen T [66] | + | - | + | + | + | + |
| Malhotra S [67] | X | - | X | - | X | + |
| Hwang [68] | + | + | + | + | - | + |
| Theuring S [69] | - | - | X | + | X | + |

Screening: institutional setting

| Study | Risk of bias items: 1 | 2 | 3 | 4 | 5 | 6 |
|---|---|---|---|---|---|---|
| London V [70] | + | + | X | - | + | + |
| Andrikopoulou M [71] | + | + | + | + | + | + |
| Bogani G [72] | + | + | X | + | + | + |
| Kirshblum SC [73] | + | + | X | + | + | + |
| Smith E [74] | + | - | + | - | + | + |
| Tanacan A [75] | X | - | - | - | + | + |
| Starling A [76] | - | - | X | - | + | + |
| Marossy A [77] | + | - | - | - | + | + |
| Viñuela MC [78] | + | + | - | - | + | + |
| Bender WR [79] | X | X | + | X | - | + |
| Shi SM [80] | X | X | - | + | + | + |
| Berghoff AS [81] | + | + | - | - | + | + |
| White EM [82] | - | + | + | + | - | + |
| Aslam A [83] | + | + | - | + | + | + |
| Adhikari EH [84] | X | - | - | - | - | + |
| Ghinai I [85] | + | - | + | X | + | X |
| Wadhwa A [86] | + | X | + | X | X | + |
| Balestrini S [87] | + | + | - | - | + | + |
| Hcini N [88] | + | + | - | - | + | + |
| van Buul LW [89] | + | - | + | - | + | + |
| Green R [90] | + | - | - | + | - | + |
| Varnell C [91] | X | - | + | - | + | - |
| Pizarro-Sánchez MS [92] | X | X | - | - | + | X |
| Vohra LM [93] | + | + | X | - | + | + |
| Isoldi S [94] | + | - | + | - | + | X |
| Haidar G [95] | X | X | + | + | + | + |
| Blain H [96] | + | + | + | + | + | + |
| Marcus JE [97] | + | + | + | - | + | + |
| Rincon A [98] | + | + | + | + | + | + |
| Martins Machado C [99] | + | - | - | - | + | + |
| Beiting KJ [100] | + | - | + | + | + | + |
| Hussain A [101] | X | - | + | - | + | + |
| Maki G [102] | X | + | - | - | + | + |
| Pamplona J [103] | X | - | X | - | + | + |
| Jeffery-Smith A [104] | - | - | + | + | + | + |
| Uçkay, I [105] | - | - | - | - | X | + |
| Say D [106] | - | - | + | - | + | + |
| Nunes MC [107] | - | - | + | + | + | + |
| Weinbergerova B [108] | X | - | + | + | + | + |
| Esteban I [109] | X | - | + | + | + | + |
| Fisher MJ [110] | + | - | X | - | X | - |
| Patel MR [111] | + | + | X | - | + | + |
| Khondaker T [112] | + | + | X | - | + | + |

Screening: occupational

| Study | Risk of bias items: 1 | 2 | 3 | 4 | 5 | 6 |
|---|---|---|---|---|---|---|
| Rivett L [113] | X | X | + | + | - | + |
| Treibel TA [114] | - | - | - | - | X | + |
| Han X [115] | X | - | - | - | X | + |
| Lombardi A [116] | + | + | + | - | + | + |
| Cariani L [117] | + | + | - | - | + | + |
| Stock AD [118] | - | - | - | - | + | + |
| Malagón-Rojas J [119] | - | - | - | X | + | + |
| Letizia AG [120] | X | - | - | - | + | + |
| Mahajan NN [121] | - | - | X | + | + | X |
| Alshahrani MS [122] | + | + | + | X | + | + |
| Hogan CA [123] | X | - | + | - | X | + |
| Ferreira VH [124] | X | - | + | + | - | + |
| Tan-Loh J [125] | - | - | + | + | + | X |
| Lalani T [126] | + | - | X | X | X | X |
| Fakhim H [127] | X | - | - | - | + | + |
| Hall VJ [128] | X | - | + | X | + | + |
| Laws RL [129] | + | + | - | - | + | + |
| Morgan SC [130] | X | - | - | - | + | + |

Judgement
+ Low
- Unclear
X High

Domains

Selection Bias:
1: Representativeness of the sample
2: Characteristics of non-respondents
Information Bias:
3: Symptom assessment
4: Recording of symptoms
Misclassification bias:
5: Classification of asymptomatic status
Attrition bias:
6: Selective reporting of symptoms status

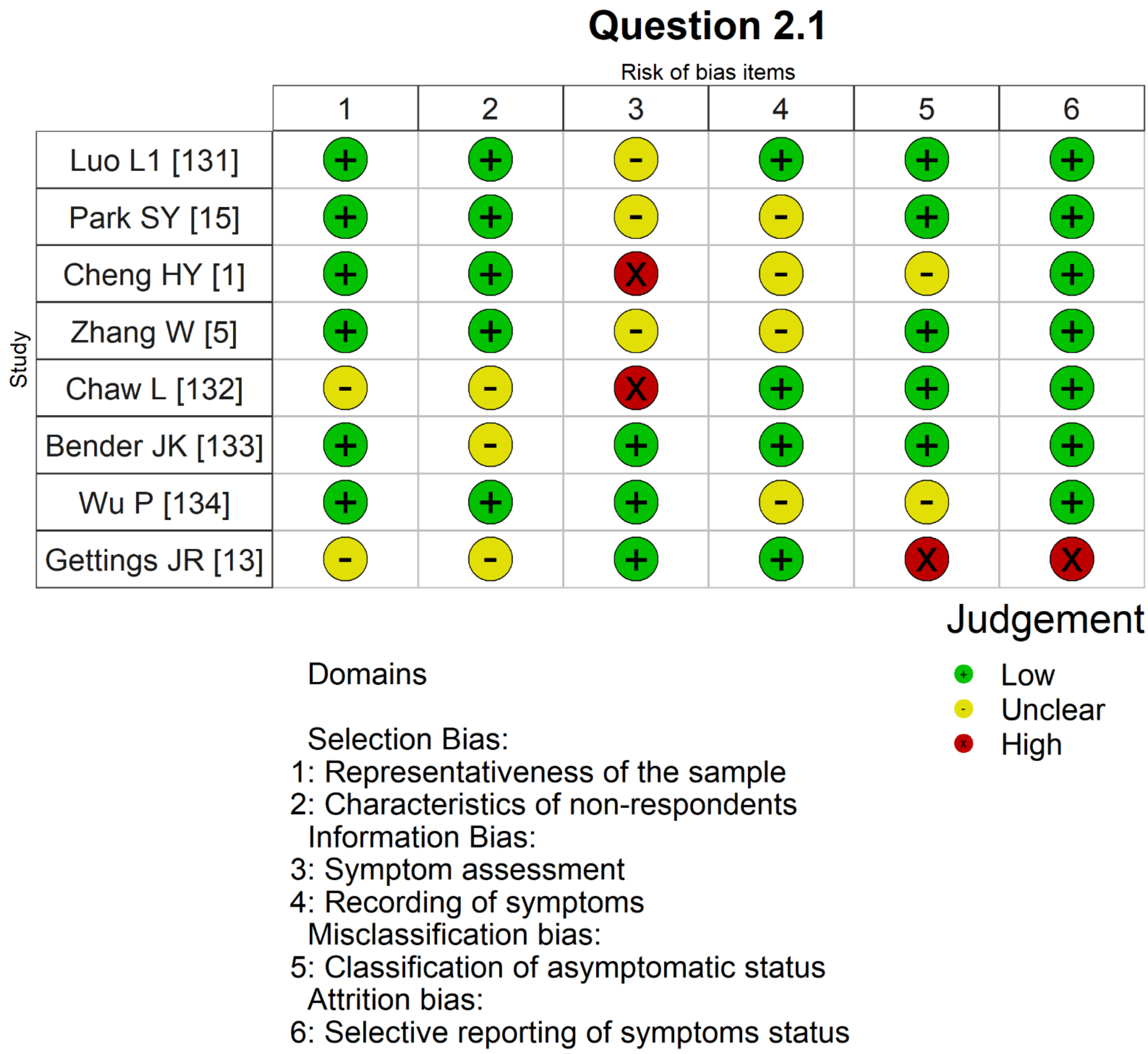
Question 2.1
Risk of bias items
1
2
3
4
5
6
Study
Luo L1 [131]
Park SY [15]
Cheng HY [1]
Zhang W [5]
Chaw L [132]
Bender JK [133]
Wu P [134]
Gettings JR [13]
Judgement
Low
Unclear
High
Domains
Selection Bias:
1: Representativeness of the sample
2: Characteristics of non-respondents
Information Bias:
3: Symptom assessment
4: Recording of symptoms
Misclassification bias:
5: Classification of asymptomatic status
Attrition bias:
6: Selective reporting of symptoms status

## References


1. Cheng HY, Jian SW, Liu DP, Ng TC, Huang WT, Lin HH, et al. Contact Tracing Assessment of Covid-19 Transmission Dynamics in Taiwan and Risk at Different Exposure Periods before and after Symptom Onset. *JAMA Intern Med*. 2020;180(9):1156-63. Epub 2020/05/02. doi: https://doi.org/10.1001/jamainternmed.2020.2020 PubMed PMID: 32356867; PubMed Central PMCID: 7195694.

2. Wu J, Huang Y, Tu C, Bi C, Chen Z, Luo L, et al. Household Transmission of Sars-Cov-2, Zhuhai, China, 2020. *Clin Infect Dis*. 2020;71(16):2099-108. Epub 2020/05/12. doi: https://doi.org/10.1093/cid/ciaa557. PubMed PMID: 32392331; PubMed Central PMCID: 7239243.

3. Hijnen D, Marzano AV, Eyerich K, GeurtsvanKessel C, Gimenez-Arnau AM, Joly P, et al. Sars-Cov-2 Transmission from Presymptomatic Meeting Attendee, Germany. *Emerg Infect Dis*. 2020;26(8):1935-7. Epub 2020/05/12. doi: https://doi.org/10.3201/eid2608.201235. PubMed PMID: 32392125; PubMed Central PMCID: 7392453.

4. Brandstetter S, Roth S, Harner S, Buntrock-Dopke H, Toncheva AA, Borchers N, et al. Symptoms and Immunoglobulin Development in Hospital Staff Exposed to a Sars-Cov-2 Outbreak. *Pediatr Allergy Immunol*. 2020;31(7):841-7. Epub 2020/05/16. doi: https://doi.org/10.1111/pai.13278. PubMed PMID: 32413201.

5. Zhang W, Cheng W, Luo L, Ma Y, Xu C, Qin P, et al. Secondary Transmission of Coronavirus Disease from Presymptomatic Persons, China. *Emerg Infect Dis*. 2020;26(8):1924-6. Epub 2020/05/27. doi: https://doi.org/10.3201/eid2608.201142. PubMed PMID: 32453686; PubMed Central PMCID: 7392433.

6. Yousaf AR, Duca LM, Chu V, Reses HE, Fajans M, Rabold EM, et al. A Prospective Cohort Study in Nonhospitalized Household Contacts with Severe Acute Respiratory Syndrome Coronavirus 2 Infection: Symptom Profiles and Symptom Change over Time. *Clin Infect Dis*. 2021;73(7):e1841-e9. Epub 2020/07/29. doi: https://doi.org/10.1093/cid/ciaa1072. PubMed PMID: 32719874; PubMed Central PMCID: 7454397.

7. Luo L, Liu D, Liao X, Wu X, Jing Q, Zheng J, et al. Contact Settings and Risk for Transmission in 3410 Close Contacts of Patients with Covid-19 in Guangzhou, China : A Prospective Cohort Study. *Ann Intern Med*. 2020;173(11):879-87. Epub 2020/08/14. doi: https://doi.org/10.7326/m20-2671. PubMed PMID: 32790510; PubMed Central PMCID: 7506769.

8. Hurst JH, Heston SM, Chambers HN, Cunningham HM, Price MJ, Suarez L, et al. Severe Acute Respiratory Syndrome Coronavirus 2 Infections among Children in the Biospecimens from Respiratory Virus-Exposed Kids (Brave Kids) Study. *Clinical Infectious Diseases*. 2021;73(9):e2875-e82. doi: https://doi.org/10.1093/cid/ciaa1693.

9. Grijalva CG, Rolfes MA, Zhu Y, McLean HQ, Hanson KE, Belongia EA, et al. Transmission of Sars-Cov-2 Infections in Households - Tennessee and Wisconsin, April-September 2020. *MMWR Morb Mortal Wkly Rep*. 2020;69(44):1631-4. Epub 2020/11/06. doi: http://dx.doi.org/10.15585/mmwr.mm6944e1. PubMed PMID: 33151916; PubMed Central PMCID: 7643897.

10. Shi Q, Hu Y, Peng B, Tang XJ, Wang W, Su K, et al. Effective Control of Sars-Cov-2 Transmission in Wanzhou, China. *Nat Med*. 2021;27(1):86-93. Epub 20201130. doi: https://doi.org/10.1038/s41591-020-01178-5. PubMed PMID: 33257893.

11. Jones A, Fialkowski V, Prinzing L, Trites J, Kelso P, Levine M. Assessment of Day-7 Postexposure Testing of Asymptomatic Contacts of Covid-19 Patients to Evaluate Early Release

from Quarantine - Vermont, May-November 2020. *MMWR Morb Mortal Wkly Rep*. 2021;70(1):12-3. Epub 2021/01/08. doi: http://dx.doi.org/10.15585/mmwr.mm7001a3. PubMed PMID: 33411700; PubMed Central PMCID: 7790157.

12. Xie W, Chen Z, Wang Q, Song M, Cao Y, Wang L, et al. Infection and Disease Spectrum in Individuals with Household Exposure to Sars-Cov-2: A Family Cluster Cohort Study. *J Med Virol*. 2021;93(5):3033-46. Epub 20210212. doi: https://doi.org/10.1002/jmv.26847. PubMed PMID: 33538342; PubMed Central PMCID: PMC8014049.

13. Gettings JR, Gold JAW, Kimball A, Forsberg K, Scott C, Uehara A, et al. Severe Acute Respiratory Syndrome Coronavirus 2 Transmission in a Georgia School District-United States, December 2020-January 2021. *Clin Infect Dis*. 2022;74(2):319-26. doi: https://doi.org/10.1093/cid/ciab332. PubMed PMID: 33864375; PubMed Central PMCID: PMC8083290.

14. Danis K, Epaulard O, Benet T, Gaymard A, Campoy S, Bothelo-Nevers E, et al. Cluster of Coronavirus Disease 2019 (Covid-19) in the French Alps, 2020. *Clin Infect Dis*. 2020. Epub 2020/04/12. doi: https://doi.org/10.1093/cid/ciaa424. PubMed PMID: 32277759; PubMed Central PMCID: 7184384.

15. Park SY, Kim YM, Yi S, Lee S, Na BJ, Kim CB, et al. Coronavirus Disease Outbreak in Call Center, South Korea. *Emerg Infect Dis*. 2020;26(8):1666-70. Epub 2020/04/24. doi: https://doi.org/10.3201/eid2608.201274. PubMed PMID: 32324530; PubMed Central PMCID: 7392450.

16. Arons MM, Hatfield KM, Reddy SC, Kimball A, James A, Jacobs JR, et al. Presymptomatic Sars-Cov-2 Infections and Transmission in a Skilled Nursing Facility. *N Engl J Med*. 2020;382(22):2081-90. Epub 2020/04/25. doi: https://doi.org/10.1056/NEJMoa2008457. PubMed PMID: 32329971; PubMed Central PMCID: 7200056.

17. Schwierzeck V, Konig JC, Kuhn J, Mellmann A, Correa-Martinez CL, Omran H, et al. First Reported Nosocomial Outbreak of Severe Acute Respiratory Syndrome Coronavirus 2 in a Pediatric Dialysis Unit. *Clin Infect Dis*. 2021;72(2):265-70. Epub 2021/01/28. doi: https://doi.org/10.1093/cid/ciaa491. PubMed PMID: 33501962; PubMed Central PMCID: 7197625.

18. Bohmer MM, Buchholz U, Corman VM, Hoch M, Katz K, Marosevic DV, et al. Investigation of a Covid-19 Outbreak in Germany Resulting from a Single Travel-Associated Primary Case: A Case Series. *Lancet Infect Dis*. 2020;20(8):920-8. Epub 2020/05/19. doi: https://doi.org/10.1016/s1473-3099(20)30314-5. PubMed PMID: 32422201; PubMed Central PMCID: 7228725.

19. Dora AV, Winnett A, Jatt LP, Davar K, Watanabe M, Sohn L, et al. Universal and Serial Laboratory Testing for Sars-Cov-2 at a Long-Term Care Skilled Nursing Facility for Veterans - Los Angeles, California, 2020. *MMWR Morb Mortal Wkly Rep*. 2020;69(21):651-5. Epub 2020/05/29. doi: https://doi.org/10.15585/mmwr.mm6921e1. PubMed PMID: 32463809; PubMed Central PMCID: 7269604.

20. Graham NSN, Junghans C, Downes R, Sendall C, Lai H, McKirdy A, et al. Sars-Cov-2 Infection, Clinical Features and Outcome of Covid-19 in United Kingdom Nursing Homes. *J Infect*. 2020;81(3):411-9. Epub 2020/06/07. doi: https://doi.org/10.1016/j.jinf.2020.05.073. PubMed PMID: 32504743; PubMed Central PMCID: 7836316.

21. Patel MC, Chaisson LH, Borgetti S, Burdsall D, Chugh RK, Hoff CR, et al. Asymptomatic Sars-Cov-2 Infection and Covid-19 Mortality During an Outbreak Investigation in a Skilled Nursing

Facility. *Clin Infect Dis*. 2020;71(11):2920-6. Epub 2020/06/18. doi: https://doi.org/10.1093/cid/ciaa763. PubMed PMID: 32548628; PubMed Central PMCID: 7337684.

22. Pavli A, Smeti P, Papadima K, Andreopoulou A, Hadjianastasiou S, Triantafillou E, et al. A Cluster of Covid-19 in Pilgrims to Israel. *J Travel Med*. 2020;27(5). Epub 2020/06/26. doi: https://doi.org/10.1093/jtm/taaa102. PubMed PMID: 32584403; PubMed Central PMCID: 7337872.

23. Njuguna H, Wallace M, Simonson S, Tobolowsky FA, James AE, Bordelon K, et al. Serial Laboratory Testing for Sars-Cov-2 Infection among Incarcerated and Detained Persons in a Correctional and Detention Facility - Louisiana, April-May 2020. *MMWR Morb Mortal Wkly Rep*. 2020;69(26):836-40. Epub 2020/07/03. doi: https://doi.org/10.15585/mmwr.mm6926e2. PubMed PMID: 32614816; PubMed Central PMCID: 7332096.

24. Yau K, Muller MP, Lin M, Siddiqui N, Neskovic S, Shokar G, et al. Covid-19 Outbreak in an Urban Hemodialysis Unit. *Am J Kidney Dis*. 2020;76(5):690-5 e1. Epub 2020/07/19. doi: https://doi.org/10.1053/j.ajkd.2020.07.001. PubMed PMID: 32681983; PubMed Central PMCID: 7362862.

25. Lee JY, Hong SW, Hyun M, Park JS, Lee JH, Suh YS, et al. Epidemiological and Clinical Characteristics of Coronavirus Disease 2019 in Daegu, South Korea. *Int J Infect Dis*. 2020;98:462-6. Epub 2020/07/24. doi: https://doi.org/10.1016/j.ijid.2020.07.017. PubMed PMID: 32702415; PubMed Central PMCID: 7371586.

26. Yang N, Shen Y, Shi C, Ma AHY, Zhang X, Jian X, et al. In-Flight Transmission Cluster of Covid-19: A Retrospective Case Series. *Infect Dis (Lond)*. 2020;52(12):891-901. Epub 2020/08/01. doi: https://doi.org/10.1080/23744235.2020.1800814. PubMed PMID: 32735163.

27. Thai PQ, Rabaa MA, Luong DH, Tan DQ, Quang TD, Quach HL, et al. The First 100 Days of Severe Acute Respiratory Syndrome Coronavirus 2 (Sars-Cov-2) Control in Vietnam. *Clin Infect Dis*. 2021;72(9):e334-e42. Epub 2020/08/02. doi: https://doi.org/10.1093/cid/ciaa1130. PubMed PMID: 32738143; PubMed Central PMCID: 7454342.

28. Plucinski MM, Wallace M, Uehara A, Kurbatova EV, Tobolowsky FA, Schneider ZD, et al. Coronavirus Disease 2019 (Covid-19) in Americans Aboard the Diamond Princess Cruise Ship. *Clin Infect Dis*. 2021;72(10):e448-e57. Epub 2020/08/14. doi: https://doi.org/10.1093/cid/ciaa1180. PubMed PMID: 32785683; PubMed Central PMCID: 7454359.

29. Kittang BR, Hofacker SV, Solheim SP, Kruger K, Loland KK, Jansen K. Outbreak of Covid-19 at Three Nursing Homes in Bergen. *Tidsskr Nor Laegeforen*. 2020;140(11). Epub 2020/08/21. doi: https://doi.org/10.4045/tidsskr.20.0405. PubMed PMID: 32815356.

30. Corcorran MA, Olin S, Rani G, Nasenbeny K, Constantino-Shor C, Holmes C, et al. Prolonged Persistence of Pcr-Detectable Virus During an Outbreak of Sars-Cov-2 in an Inpatient Geriatric Psychiatry Unit in King County, Washington. *Am J Infect Control*. 2021;49(3):293-8. Epub 2020/08/23. doi: https://doi.org/10.1016/j.ajic.2020.08.025. PubMed PMID: 32827597; PubMed Central PMCID: 7438365.

31. Pirnay JP, Selhorst P, Cochez C, Petrillo M, Claes V, Van der Beken Y, et al. Study of a Sars-Cov-2 Outbreak in a Belgian Military Education and Training Center in Maradi, Niger. *Viruses*. 2020;12(9). Epub 2020/09/02. doi: https://doi.org/10.3390/v12090949. PubMed PMID: 32867108; PubMed Central PMCID: 7552053.

32. Taylor J, Carter RJ, Lehnertz N, Kazazian L, Sullivan M, Wang X, et al. Serial Testing for Sars-Cov-2 and Virus Whole Genome Sequencing Inform Infection Risk at Two Skilled Nursing Facilities with Covid-19 Outbreaks - Minnesota, April-June 2020. *MMWR Morb Mortal Wkly Rep*.

2020;69(37):1288-95. Epub 2020/09/24. doi: https://doi.org/10.15585/mmwr.mm6937a3. PubMed PMID: 32966272; PubMed Central PMCID: 7498172.

33. Kennelly SP, Dyer AH, Noonan C, Martin R, Kennelly SM, Martin A, et al. Asymptomatic Carriage Rates and Case Fatality of Sars-Cov-2 Infection in Residents and Staff in Irish Nursing Homes. *Age Ageing*. 2021;50(1):49-54. Epub 2020/09/29. doi: https://doi.org/10.1093/ageing/afaa220. PubMed PMID: 32986806; PubMed Central PMCID: 7543256.

34. Romao VC, Oliveira-Ramos F, Cruz-Machado AR, Martins P, Barreira S, Silva-Dinis J, et al. A Covid-19 Outbreak in a Rheumatology Department Upon the Early Days of the Pandemic. *Front Med (Lausanne)*. 2020;7:576162. Epub 2020/10/27. doi: https://doi.org/10.3389/fmed.2020.576162. PubMed PMID: 33102507; PubMed Central PMCID: 7546334.

35. Ladhani SN, Jeffery-Smith A, Patel M, Janarthanan R, Fok J, Crawley-Boevey E, et al. High Prevalence of Sars-Cov-2 Antibodies in Care Homes Affected by Covid-19: Prospective Cohort Study, England. *EClinicalMedicine*. 2020;28:100597. Epub 2020/11/12. doi: https://doi.org/10.1016/j.eclinm.2020.100597. PubMed PMID: 33173854; PubMed Central PMCID: 7644437.

36. Kasper MR, Geibe JR, Sears CL, Riegodedios AJ, Luse T, Von Thun AM, et al. An Outbreak of Covid-19 on an Aircraft Carrier. *N Engl J Med*. 2020;383(25):2417-26. Epub 2020/11/12. doi: https://doi.org/10.1056/nejmoa2019375. PubMed PMID: 33176077; PubMed Central PMCID: 7675688.

37. Redditt V, Wright V, Rashid M, Male R, Bogoch I. Outbreak of Sars-Cov-2 Infection at a Large Refugee Shelter in Toronto, April 2020: A Clinical and Epidemiologic Descriptive Analysis. *CMAJ Open*. 2020;8(4):E819-E24. Epub 2020/12/10. doi: https://doi.org/10.9778/cmajo.20200165. PubMed PMID: 33293331; PubMed Central PMCID: 7743908.

38. Park JH, Jang JH, Lee K, Yoo SJ, Shin H. Covid-19 Outbreak and Presymptomatic Transmission in Pilgrim Travelers Who Returned to Korea from Israel. *J Korean Med Sci*. 2020;35(48):e424. Epub 2020/12/15. doi: https://doi.org/10.3346/jkms.2020.35.e424. PubMed PMID: 33316860; PubMed Central PMCID: 7735917.

39. Harada S, Uno S, Ando T, Iida M, Takano Y, Ishibashi Y, et al. Control of a Nosocomial Outbreak of Covid-19 in a University Hospital. *Open Forum Infect Dis*. 2020;7(12):ofaa512. Epub 2020/12/18. doi: https://doi.org/10.1093/ofid/ofaa512. PubMed PMID: 33330740; PubMed Central PMCID: 7665726.

40. Garibaldi PMM, Ferreira NN, Moraes GR, Moura JC, Espósito DLA, Volpe GJ, et al. Efficacy of Covid-19 Outbreak Management in a Skilled Nursing Facility Based on Serial Testing for Early Detection and Control. *Braz J Infect Dis*. 2021;25(2):101570. Epub 20210323. doi: https://doi.org/10.1016/j.bjid.2021.101570. PubMed PMID: 33773990; PubMed Central PMCID: PMC7985963.

41. Tian S, Wu M, Chang Z, Wang Y, Zhou G, Zhang W, et al. Epidemiological Investigation and Intergenerational Clinical Characteristics of 24 Coronavirus Disease Patients Associated with a Supermarket Cluster: A Retrospective Study. *BMC Public Health*. 2021;21(1):647. Epub 2021/04/03. doi: https://doi.org/10.1186/s12889-021-10713-z. PubMed PMID: 33794836; PubMed Central PMCID: 8016429.

42. van den Besselaar JH, Sikkema RS, Koene F, van Buul LW, Oude Munnink BB, Frenay I, et al. Are Presymptomatic Sars-Cov-2 Infections in Nursing Home Residents Unrecognised

Symptomatic Infections? Sequence and Metadata from Weekly Testing in an Extensive Nursing Home Outbreak. *Age Ageing*. 2021;50(5):1454-63. Epub 2021/05/09. doi: https://doi.org/10.1093/ageing/afab081. PubMed PMID: 33963830; PubMed Central PMCID: 8136016.

43. Orsi A, Domnich A, Pace VD, Ricucci V, Caligiuri P, Bottiglieri L, et al. Outbreak of Sars-Cov-2 Lineage 20i/501y.V1 in a Nursing Home Underlines the Crucial Role of Vaccination in Both Residents and Staff. *Vaccines*. 2021;9(6):591. PubMed PMID: https://doi.org/10.3390/vaccines9060591.

44. Schmitt J, Genet E, Danguy Des Deserts M, Chauvet-Atin S, Cungi PJ, Aries P, et al. Sars Cov2 Outbreak Management on a Landing Helicopter Dock: An Observational Retrospective Study. *Infect Dis Now*. 2021;51(5):424-8. Epub 20210619. doi: https://doi.org/10.1016/j.idnow.2021.06.303. PubMed PMID: 34157429; PubMed Central PMCID: PMC8214318.

45. Paleker M, Tembo YA, Davies MA, Mahomed H, Pienaar D, Madhi SA, et al. Asymptomatic Covid-19 in South Africa - Implications for the Control of Transmission. *Public Health Action*. 2021;11(2):58-60. doi: https://doi.org/10.5588/pha.20.0069. PubMed PMID: 34159063; PubMed Central PMCID: PMC8202632.

46. Cardillo L, de Martinis C, Viscardi M, Esposito C, Sannino E, Lucibelli G, et al. Sars-Cov-2 Quantitative Real Time Pcr and Viral Loads Analysis among Asymptomatic and Symptomatic Patients: An Observational Study on an Outbreak in Two Nursing Facilities in Campania Region (Southern Italy). *Infect Agent Cancer*. 2021;16(1):45. Epub 20210622. doi: https://doi.org/10.1186/s13027-021-00388-x. PubMed PMID: 34158108; PubMed Central PMCID: PMC8218569.

47. Chang L, Zhao L, Gong H, Wang L, Wang L. Severe Acute Respiratory Syndrome Coronavirus 2 Rna Detected in Blood Donations. *Emerg Infect Dis*. 2020;26(7):1631-3. Epub 2020/04/04. doi: https://doi.org/10.3201/eid2607.200839. PubMed PMID: 32243255; PubMed Central PMCID: 7323524.

48. Lavezzo E, Franchin E, Ciavarella C, Cuomo-Dannenburg G, Barzon L, Del Vecchio C, et al. Suppression of a Sars-Cov-2 Outbreak in the Italian Municipality of Vo'. *Nature*. 2020;584(7821):425-9. Epub 2020/07/01. doi: https://doi.org/10.1038/s41586-020-2488-1. PubMed PMID: 32604404.

49. Hoehl S, Rabenau H, Berger A, Kortenbusch M, Cinatl J, Bojkova D, et al. Evidence of Sars-Cov-2 Infection in Returning Travelers from Wuhan, China. *N Engl J Med*. 2020;382(13):1278-80. Epub 2020/02/19. doi: https://doi.org/10.1056/nejmc2001899. PubMed PMID: 32069388; PubMed Central PMCID: 7121749.

50. Wong J, Abdul Aziz ABZ, Chaw L, Mahamud A, Griffith MM, Ying-Ru LO, et al. High Proportion of Asymptomatic and Presymptomatic Covid-19 Infections in Travelers and Returning Residents to Brunei. *J Travel Med*. 2020. doi: https://doi.org/10.1093/jtm/taaa066.

51. Almazeedi S, Al-Youha S, Jamal MH, Al-Haddad M, Al-Muhaini A, Al-Ghimlas F, et al. Characteristics, Risk Factors and Outcomes among the First Consecutive 1096 Patients Diagnosed with Covid-19 in Kuwait. *EClinicalMedicine*. 2020;24:100448. Epub 2020/08/09. doi: https://doi.org/10.1016/j.eclinm.2020.100448. PubMed PMID: 32766546; PubMed Central PMCID: 7335246.

52. Wi YM, Lim SJ, Kim SH, Lim S, Lee SJ, Ryu BH, et al. Response System for and Epidemiological Features of Covid-19 in Gyeongsangnam-Do Province in South Korea. *Clin Infect*

*Dis*. 2021;72(4):661-7. Epub 2020/07/17. doi: https://doi.org/10.1093/cid/ciaa967. PubMed PMID: 32672789; PubMed Central PMCID: 7454481.

53. Kutsuna S, Suzuki T, Hayakawa K, Tsuzuki S, Asai Y, Suzuki T, et al. Sars-Cov-2 Screening Test for Japanese Returnees from Wuhan, China, January 2020. *Open Forum Infect Dis*. 2020;7(7):ofaa243. Epub 2020/08/06. doi: https://doi.org/10.1093/ofid/ofaa243. PubMed PMID: 32754627; PubMed Central PMCID: 7337761.

54. Eythorsson E, Helgason D, Ingvarsson RF, Bjornsson HK, Olafsdottir LB, Bjarnadottir V, et al. Clinical Spectrum of Coronavirus Disease 2019 in Iceland: Population Based Cohort Study. *BMJ*. 2020;371:m4529. Epub 2020/12/04. doi: https://doi.org/10.1136/bmj.m4529. PubMed PMID: 33268329; PubMed Central PMCID: 7708618.

55. Chamie G, Marquez C, Crawford E, Peng J, Petersen M, Schwab D, et al. Community Transmission of Severe Acute Respiratory Syndrome Coronavirus 2 Disproportionately Affects the Latinx Population During Shelter-in-Place in San Francisco. *Clin Infect Dis*. 2021;73(Suppl 2):S127-S35. Epub 2020/08/22. doi: https://doi.org/10.1093/cid/ciaa1234. PubMed PMID: 32821935; PubMed Central PMCID: 7499499.

56. AbdulRahman A, AlAli S, Yaghi O, Shabaan M, Otoom S, Atkin SL, et al. Covid-19 and Sickle Cell Disease in Bahrain. *Int J Infect Dis*. 2020;101:14-6. Epub 2020/09/28. doi: https://doi.org/10.1016/j.ijid.2020.09.1433. PubMed PMID: 32980556; PubMed Central PMCID: 7833176.

57. Edelstein M, Obi C, Chand M, Hopkins S, Brown K, Ramsay M. Sars-Cov-2 Infection in London, England: Changes to Community Point Prevalence around Lockdown Time, March-May 2020. *J Epidemiol Community Health*. 2021;75(2):185-8. Epub 2020/10/03. doi: https://doi.org/10.1136/jech-2020-214730. PubMed PMID: 33004659; PubMed Central PMCID: 7815898.

58. Al-Qahtani M, AlAli S, AbdulRahman A, Salman Alsayyad A, Otoom S, Atkin SL. The Prevalence of Asymptomatic and Symptomatic Covid-19 in a Cohort of Quarantined Subjects. *Int J Infect Dis*. 2021;102:285-8. Epub 2020/11/07. doi: https://doi.org/10.1016/j.ijid.2020.10.091. PubMed PMID: 33157290; PubMed Central PMCID: 7607262.

59. Cao S, Gan Y, Wang C, Bachmann M, Wei S, Gong J, et al. Post-Lockdown Sars-Cov-2 Nucleic Acid Screening in Nearly Ten Million Residents of Wuhan, China. *Nat Commun*. 2020;11(1):5917. Epub 2020/11/22. doi: https://doi.org/10.1038/s41467-020-19802-w. PubMed PMID: 33219229; PubMed Central PMCID: 7679396.

60. Migisha R, Kwesiga B, Mirembe BB, Amanya G, Kabwama SN, Kadobera D, et al. Early Cases of Sars-Cov-2 Infection in Uganda: Epidemiology and Lessons Learned from Risk-Based Testing Approaches - March-April 2020. *Global Health*. 2020;16(1):114. Epub 2020/11/27. doi: https://doi.org/10.1186/s12992-020-00643-7. PubMed PMID: 33239041; PubMed Central PMCID: 7686950.

61. Meyers KJ, Dillman B, Williams C, Jiang J, Clifford N, Miller JL, et al. Follow-up of Sars-Cov-2 Positive Subgroup from the Asymptomatic Novel Coronavirus Infection Study. *J Med Virol*. 2021;93(5):2925-31. Epub 2021/01/20. doi: https://doi.org/10.1002/jmv.26810. PubMed PMID: 33463731; PubMed Central PMCID: 8014630.

62. Ren R, Zhang Y, Li Q, McGoogan JM, Feng Z, Gao GF, et al. Asymptomatic Sars-Cov-2 Infections among Persons Entering China from April 16 to October 12, 2020. *Jama*. 2021;325(5):489-92. doi: https://doi.org/10.1001/jama.2020.23942. PubMed PMID: 33528529; PubMed Central PMCID: PMC7856538.

63. Rauch JN, Valois E, Ponce-Rojas JC, Aralis Z, Lach RS, Zappa F, et al. Comparison of Severe Acute Respiratory Syndrome Coronavirus 2 Screening Using Reverse Transcriptase-Quantitative Polymerase Chain Reaction or Crispr-Based Assays in Asymptomatic College Students. *JAMA Netw Open*. 2021;4(2):e2037129. Epub 2021/02/12. doi: https://doi.org/10.1001/jamanetworkopen.2020.37129. PubMed PMID: 33570576; PubMed Central PMCID: 7879237.

64. Uysal E, Kilinçer A, Cebeci H, Özer H, Demir NA, Öztürk M, et al. Chest Ct Findings in Rt-Pcr Positive Asymptomatic Covid-19 Patients. *Clinical Imaging*. 2021;77:37-42. doi: https://doi.org/10.1016/j.clinimag.2021.01.030.

65. Abraha HE, Gessesse Z, Gebrecherkos T, Kebede Y, Weldegiargis AW, Tequare MH, et al. Clinical Features and Risk Factors Associated with Morbidity and Mortality among Patients with Covid-19 in Northern Ethiopia. *Int J Infect Dis*. 2021;105:776-83. Epub 20210316. doi: https://doi.org/10.1016/j.ijid.2021.03.037. PubMed PMID: 33741488; PubMed Central PMCID: PMC7962557.

66. Turunen T, Kontunen K, Sugulle K, Hieta P, Snellman O, Hussein I, et al. Covid-19 Outbreak at a Reception Centre for Asylum Seekers in Espoo, Finland. *J Migr Health*. 2021;3:100043. Epub 20210416. doi: https://doi.org/10.1016/j.jmh.2021.100043. PubMed PMID: 33880457; PubMed Central PMCID: PMC8051004.

67. Malhotra S, Rahi M, Das P, Chaturvedi R, Chhibber-Goel J, Anvikar A, et al. Epidemiological Profiles and Associated Risk Factors of Sars-Cov-2 Positive Patients Based on a High-Throughput Testing Facility in India. *Open Biol*. 2021;11(6):200288. Epub 20210602. doi: https://doi.org/10.1098/rsob.200288. PubMed PMID: 34062097; PubMed Central PMCID: PMC8169211.

68. Hwang CE, Kussman A, Christle JW, Froelicher V, Wheeler MT, Moneghetti KJ. Findings from Cardiovascular Evaluation of National Collegiate Athletic Association Division I Collegiate Student-Athletes after Asymptomatic or Mildly Symptomatic Sars-Cov-2 Infection. *Clin J Sport Med*. 2022;32(2):103-7. doi: https://doi.org/10.1097/jsm.0000000000000954. PubMed PMID: 34173780.

69. Theuring S, Thielecke M, van Loon W, Hommes F, Hulso C, von der Haar A, et al. Sars-Cov-2 Infection and Transmission in School Settings During the Second Covid-19 Wave: A Cross-Sectional Study, Berlin, Germany, November 2020. *Euro Surveill*. 2021;26(34). Epub 2021/08/28. doi: https://doi.org/10.2807/1560-7917.es.2021.26.34.2100184. PubMed PMID: 34448448; PubMed Central PMCID: 8393892.

70. London V, McLaren R, Jr., Atallah F, Cepeda C, McCalla S, Fisher N, et al. The Relationship between Status at Presentation and Outcomes among Pregnant Women with Covid-19. *Am J Perinatol*. 2020;37(10):991-4. Epub 2020/05/20. doi: https://doi.org/10.1055/s-0040-1712164. PubMed PMID: 32428964; PubMed Central PMCID: 7416203.

71. Andrikopoulou M, Madden N, Wen T, Aubey JJ, Aziz A, Baptiste CD, et al. Symptoms and Critical Illness among Obstetric Patients with Coronavirus Disease 2019 (Covid-19) Infection. *Obstet Gynecol*. 2020;136(2):291-9. Epub 2020/05/28. doi: https://doi.org/10.1097/aog.0000000000003996. PubMed PMID: 32459701.

72. Bogani G, Ditto A, Bosio S, Brusadelli C, Raspagliesi F. Cancer Patients Affected by Covid-19: Experience from Milan, Lombardy. *Gynecol Oncol*. 2020;158(2):262-5. Epub 2020/06/15. doi: https://doi.org/10.1016/j.ygyno.2020.06.161. PubMed PMID: 32534808; PubMed Central PMCID: 7286266.

73. Kirshblum SC, DeLauter G, Lopreiato MC, Pomeranz B, Dawson A, Hammerman S, et al. Screening Testing for Sars-Cov-2 Upon Admission to Rehabilitation Hospitals in a High Covid-19 Prevalence Community. *PM R*. 2020;12(10):1009-14. Epub 2020/07/24. doi: https://doi.org/10.1002/pmrj.12454. PubMed PMID: 32700434; PubMed Central PMCID: 7404891.

74. Smith E, Aldus CF, Brainard J, Dunham S, Hunter PR, Steel N, et al. Testing for Sars-Cov-2 in Care Home Staff and Residents in English Care Homes: A Service Evaluation. *medRxiv [Preprint]*. 2020. doi: https://doi.org/10.1101/2020.08.04.20165928.

75. Tanacan A, Erol SA, Turgay B, Anuk AT, Secen EI, Yegin GF, et al. The Rate of Sars-Cov-2 Positivity in Asymptomatic Pregnant Women Admitted to Hospital for Delivery: Experience of a Pandemic Center in Turkey. *Eur J Obstet Gynecol Reprod Biol*. 2020;253:31-4. Epub 2020/08/09. doi: https://doi.org/10.1016/j.ejogrb.2020.07.051. PubMed PMID: 32763728; PubMed Central PMCID: 7390745.

76. Starling A, White E, Showell D, Wyllie D, Kapadia S, Balakrishnan R. Whole Care Home Testing for Covid-19 in a Local Authority Area in the United Kingdom. *medRxiv [Preprint]*. 2020. doi: https://doi.org/10.1101/2020.08.06.20162859.

77. Marossy A, Rakowicz S, Bhan A, Noon S, Rees A, Virk M, et al. A Study of Universal Severe Acute Respiratory Syndrome Coronavirus 2 Rna Testing among Residents and Staff in a Large Group of Care Homes in South London. *J Infect Dis*. 2021;223(3):381-8. Epub 2020/09/06. doi: https://doi.org/10.1093/infdis/jiaa565. PubMed PMID: 32889532; PubMed Central PMCID: 7499645.

78. Vinuela MC, De Leon-Luis JA, Alonso R, Catalan P, Lizarraga S, Munoz P, et al. Sars-Cov-2 Screening of Asymptomatic Women Admitted for Delivery Must Be Performed with a Combination of Microbiological Techniques: An Observational Study. *Rev Esp Quimioter*. 2020;33(6):415-21. Epub 2020/09/19. doi: https://doi.org/10.37201/req/088.2020. PubMed PMID: 32945157; PubMed Central PMCID: 7712338.

79. Bender WR, Hirshberg A, Coutifaris P, Acker AL, Srinivas SK. Universal Testing for Severe Acute Respiratory Syndrome Coronavirus 2 in 2 Philadelphia Hospitals: Carrier Prevalence and Symptom Development over 2 Weeks. *Am J Obstet Gynecol MFM*. 2020;2(4):100226. Epub 2020/09/22. doi: https://doi.org/10.1016/j.ajogmf.2020.100226. PubMed PMID: 32954248; PubMed Central PMCID: 7485442.

80. Shi SM, Bakaev I, Chen H, Travison TG, Berry SD. Risk Factors, Presentation, and Course of Coronavirus Disease 2019 in a Large, Academic Long-Term Care Facility. *J Am Med Dir Assoc*. 2020;21(10):1378-83.e1. Epub 20200825. doi: https://doi.org/10.1016/j.jamda.2020.08.027. PubMed PMID: 32981664; PubMed Central PMCID: 7447263.

81. Berghoff AS, Gansterer M, Bathke AC, Trutschnig W, Hungerlander P, Berger JM, et al. Sars-Cov-2 Testing in Patients with Cancer Treated at a Tertiary Care Hospital During the Covid-19 Pandemic. *J Clin Oncol*. 2020;38(30):3547-54. Epub 2020/08/17. doi: https://doi.org/10.1200/jco.20.01442. PubMed PMID: 32795227; PubMed Central PMCID: 7571795.

82. White EM, Santostefano CM, Feifer RA, Kosar CM, Blackman C, Gravenstein S, et al. Asymptomatic and Presymptomatic Severe Acute Respiratory Syndrome Coronavirus 2 Infection Rates in a Multistate Sample of Skilled Nursing Facilities. *JAMA Intern Med*. 2020;180(12):1709-11. Epub 2020/10/20. doi: https://doi.org/10.1001/jamainternmed.2020.5664. PubMed PMID: 33074318; PubMed Central PMCID: 7573793.

83. Aslam A, Singh J, Robilotti E, Chow K, Bist T, Reidy-Lagunes D, et al. Severe Acute Respiratory Syndrome Coronavirus 2 Surveillance and Exposure in the Perioperative Setting with Universal Testing and Personal Protective Equipment Policies. *Clin Infect Dis*. 2021;73(9):e3013-e8. Epub 2020/10/23. doi: https://doi.org/10.1093/cid/ciaa1607. PubMed PMID: 33090210; PubMed Central PMCID: 7665395.

84. Adhikari EH, Moreno W, Zofkie AC, MacDonald L, McIntire DD, Collins RRJ, et al. Pregnancy Outcomes among Women with and without Severe Acute Respiratory Syndrome Coronavirus 2 Infection. *JAMA Netw Open*. 2020;3(11):e2029256. Epub 2020/11/20. doi: https://doi.org/10.1001/jamanetworkopen.2020.29256. PubMed PMID: 33211113; PubMed Central PMCID: 7677755.

85. Ghinai I, Davis ES, Mayer S, Toews KA, Huggett TD, Snow-Hill N, et al. Risk Factors for Severe Acute Respiratory Syndrome Coronavirus 2 Infection in Homeless Shelters in Chicago, Illinois-March-May, 2020. *Open Forum Infect Dis*. 2020;7(11):ofaa477. Epub 2020/12/03. doi: https://doi.org/10.1093/ofid/ofaa477. PubMed PMID: 33263069; PubMed Central PMCID: 7665740.

86. Wadhwa A, Fisher KA, Silver R, Koh M, Arons MM, Miller DA, et al. Identification of Presymptomatic and Asymptomatic Cases Using Cohort-Based Testing Approaches at a Large Correctional Facility-Chicago, Illinois, USA, May 2020. *Clin Infect Dis*. 2021;72(5):e128-e35. Epub 2020/12/04. doi: https://doi.org/10.1093/cid/ciaa1802. PubMed PMID: 33270101; PubMed Central PMCID: 7799274.

87. Balestrini S, Koepp MJ, Gandhi S, Rickman HM, Shin GY, Houlihan CF, et al. Clinical Outcomes of Covid-19 in Long-Term Care Facilities for People with Epilepsy. *Epilepsy Behav*. 2021;115:107602. Epub 2020/12/07. doi: https://doi.org/10.1016/j.yebeh.2020.107602. PubMed PMID: 33279440; PubMed Central PMCID: 7643621.

88. Hcini N, Maamri F, Picone O, Carod JF, Lambert V, Mathieu M, et al. Maternal, Fetal and Neonatal Outcomes of Large Series of Sars-Cov-2 Positive Pregnancies in Peripartum Period: A Single-Center Prospective Comparative Study. *Eur J Obstet Gynecol Reprod Biol*. 2021;257:11-8. Epub 2020/12/15. doi: https://doi.org/10.1016/j.ejogrb.2020.11.068. PubMed PMID: 33310656; PubMed Central PMCID: 7705341.

89. van Buul LW, van den Besselaar JH, Koene F, Buurman BM, Hertogh C, Group** C-N-S, et al. Asymptomatic Cases and Limited Transmission of Sars-Cov-2 in Residents and Healthcare Workers in Three Dutch Nursing Homes. *Gerontol Geriatr Med*. 2020;6:2333721420982800. Epub 2021/01/12. doi: https://doi.org/10.1177/2333721420982800. PubMed PMID: 33426178; PubMed Central PMCID: 7756037.

90. Green R, Tulloch JSP, Tunnah C, Coffey E, Lawrenson K, Fox A, et al. Covid-19 Testing in Outbreak-Free Care Homes: What Are the Public Health Benefits? *J Hosp Infect*. 2021;111:89-95. Epub 2021/01/17. doi: https://doi.org/10.1016/j.jhin.2020.12.024. PubMed PMID: 33453349; PubMed Central PMCID: 7837210.

91. Varnell C, Jr., Harshman LA, Smith L, Liu C, Chen S, Al-Akash S, et al. Covid-19 in Pediatric Kidney Transplantation: The Improving Renal Outcomes Collaborative. *Am J Transplant*. 2021;21(8):2740-8. Epub 2021/01/17. doi: https://doi.org/10.1111/ajt.16501. PubMed PMID: 33452854; PubMed Central PMCID: 8013281.

92. Pizarro-Sanchez MS, Avello A, Mas-Fontao S, Stock da Cunha T, Goma-Garces E, Pereira M, et al. Clinical Features of Asymptomatic Sars-Cov-2 Infection in Hemodialysis Patients. *Kidney Blood Press Res*. 2021;46(1):126-34. Epub 2021/01/28. doi: https://doi.org/10.1159/000512535. PubMed PMID: 33503627; PubMed Central PMCID: 7900451.

93. Vohra LM, Jabeen D, Asif N, Ahad A. Covid-19 Pandemic and Breast Cancer Management: A Retrospective Observational Clinical Study from Pakistan. *Ann Med Surg (Lond)*. 2021;63:102151. Epub 20210204. doi: https://doi.org/10.1016/j.amsu.2021.01.099. PubMed PMID: 33564461; PubMed Central PMCID: PMC7860941.

94. Isoldi S, Mallardo S, Marcellino A, Bloise S, Dilillo A, Iorfida D, et al. The Comprehensive Clinic, Laboratory, and Instrumental Evaluation of Children with Covid-19: A 6-Months Prospective Study. *J Med Virol*. 2021;93(5):3122-32. Epub 20210216. doi: https://doi.org/10.1002/jmv.26871. PubMed PMID: 33570199; PubMed Central PMCID: PMC8014060.

95. Haidar G, Ayres A, King WC, McDonald M, Wells A, Mitchell SL, et al. Preprocedural Sars-Cov-2 Testing to Sustain Medically Needed Health Care Delivery During the Covid-19 Pandemic: A Prospective Observational Study. *Open Forum Infectious Diseases*. 2021;8(2). doi: https://doi.org/10.1093/ofid/ofab022.

96. Blain H, Gamon L, Tuaillon E, Pisoni A, Giacosa N, Albrand M, et al. Atypical Symptoms, Sars-Cov-2 Test Results and Immunisation Rates in 456 Residents from Eight Nursing Homes Facing a Covid-19 Outbreak. *Age Ageing*. 2021;50(3):641-8. doi: https://doi.org/10.1093/ageing/afab050. PubMed PMID: 33620381; PubMed Central PMCID: PMC7929417.

97. Marcus JE, Frankel DN, Pawlak MT, Casey TM, Cybulski RJ, Jr., Enriquez E, et al. Risk Factors Associated with Covid-19 Transmission among Us Air Force Trainees in a Congregant Setting. *JAMA Netw Open*. 2021;4(2):e210202. Epub 20210201. doi: https://doi.org/10.1001/jamanetworkopen.2021.0202. PubMed PMID: 33630090; PubMed Central PMCID: PMC7907953.

98. Rincón A, Moreso F, López-Herradón A, Fernández-Robres MA, Cidraque I, Nin J, et al. The Keys to Control a Covid-19 Outbreak in a Haemodialysis Unit. *Clinical Kidney Journal*. 2020;13(4):542-9. doi: https://doi.org/10.1093/ckj/sfaa119.

99. Machado CM, Kerbauy MN, Colturato I, Arcuri LJ, dos Santos ACF, Silva FR, et al. Clinical Characteristics and Outcomes of Covid-19 in Hsct Recipients. *Blood*. 2020;136:19-. Epub 2021/08/03. doi: https://doi.org/10.1182/blood-2020-141959. PubMed PMID: PMC8330389.

100. Beiting KJ, Huisingh-Scheetz M, Walker J, Graupner J, Martinchek M, Thompson K, et al. Management and Outcomes of a Covid-19 Outbreak in a Nursing Home with Predominantly Black Residents. *J Am Geriatr Soc*. 2021;69(5):1155-65. Epub 20210324. doi: https://doi.org/10.1111/jgs.17126. PubMed PMID: 33739444; PubMed Central PMCID: PMC8218575.

101. Hussain A, Satti L, Hanif F, Shoaib M, Ghauri MA, Khan Niazi GA, et al. Clinical and Virological Course of Sars-Cov 2 Infected Patients in a Tertiary Care Hospital in Pakistan. *J Ayub Med Coll Abbottabad*. 2020;32(Suppl 1)(4):S602-s6. doi: https://pubmed.ncbi.nlm.nih.gov/33754515/. PubMed PMID: 33754515; PubMed Central PMCID: 33754515.

102. Maki G, Abdollah F, Dabaja A, Suleyman G. 462. Prevalence and Outcome of Asymptomatic Procedural Patients with Covid-19 Infection. *Open Forum Infectious Diseases*. 2020;7(Supplement_1):S298-S. doi: https://doi.org/10.1093/ofid/ofaa439.655.

103. Pamplona J, Solano R, Ramírez M, Durandez R, Mohamed F, Pardo L, et al. High Prevalence of Sars-Cov-2 Infection in Patients Scheduled for Digestive Endoscopy after the Peak of the First Wave of the Pandemic. *Gastroenterol Hepatol*. 2021;44(9):614-9. Epub 20210420. doi:

https://doi.org/10.1016/j.gastrohep.2021.03.001. PubMed PMID: 33862154; PubMed Central PMCID: PMC8056966.

104. Jeffery-Smith A, Dun-Campbell K, Janarthanan R, Fok J, Crawley-Boevey E, Vusirikala A, et al. Infection and Transmission of Sars-Cov-2 in London Care Homes Reporting No Cases or Outbreaks of Covid-19: Prospective Observational Cohort Study, England 2020. *Lancet Reg Health Eur*. 2021;3:100038. Epub 20210122. doi: https://doi.org/10.1016/j.lanepe.2021.100038. PubMed PMID: 33870248; PubMed Central PMCID: PMC7826003.

105. Uçkay I, Steinwender L, Burkhard J, Holy D, Strähl M, Farshad M. Outcomes of Asymptomatic Hospital Employees in Covid-19 Post-Exposure Quarantine During the Second Pandemic Wave in Zurich. *J Hosp Infect*. 2021;113:189-91. Epub 20210421. doi: https://doi.org/10.1016/j.jhin.2021.04.010. PubMed PMID: 33891983; PubMed Central PMCID: PMC8057933.

106. Say D, Crawford N, McNab S, Wurzel D, Steer A, Tosif S. Post-Acute Covid-19 Outcomes in Children with Mild and Asymptomatic Disease. *Lancet Child Adolesc Health*. 2021;5(6):e22-e3. Epub 20210421. doi: https://doi.org/10.1016/s2352-4642(21)00124-3. PubMed PMID: 33891880; PubMed Central PMCID: PMC8057863.

107. Nunes MC, Baillie VL, Kwatra G, Bhikha S, Verwey C, Menezes C, et al. Severe Acute Respiratory Syndrome Coronavirus 2 Infection among Healthcare Workers in South Africa: A Longitudinal Cohort Study. *Clin Infect Dis*. 2021;73(10):1896-900. doi: https://doi.org/10.1093/cid/ciab398. PubMed PMID: 33949670; PubMed Central PMCID: PMC8135922.

108. Weinbergerova B, Mayer J, Hrabovsky S, Novakova Z, Pospisil Z, Martykanova L, et al. Covid-19's Natural Course among Ambulatory Monitored Outpatients. *Sci Rep*. 2021;11(1):10124. Epub 20210512. doi: https://doi.org/10.1038/s41598-021-89545-1. PubMed PMID: 33980931; PubMed Central PMCID: PMC8115337.

109. Esteban I, Bergero G, Alves C, Bronstein M, Ziegler V, Wood C, et al. Asymptomatic Covid-19 in the Elderly: Dementia and Viral Clearance as Risk Factors for Disease Progression. *TP92 TP092 CLINICAL ADVANCES IN SARS-COV-2 AND COVID-19*. 2021:A3826-A. doi: https://doi.org/10.1164/ajrccm-conference.2021.203.1_MeetingAbstracts.A3826.

110. Fisher MJ, Psevdos G. Asymptomatic Sars-Cov-2 Infection Following First Dose Mrna-1273 Covid-19 Vaccine in a Veterans Affairs Long Term Care Facility. *Am J Infect Control*. 2021;49(9):1210-1. Epub 20210625. doi: https://doi.org/10.1016/j.ajic.2021.06.010. PubMed PMID: 34175364; PubMed Central PMCID: PMC8225993.

111. Patel MR, Kushwaha RS, Behera M, Bhadauria DS, Yachha M, Kaul A, et al. Aftermath of Fortnightly Universal Testing for Severe Acute Respiratory Corona Virus-2 Infection in Maintenance Hemodialysis Patients. *Semin Dial*. 2021;34(5):338-46. Epub 20210624. doi: https://doi.org/10.1111/sdi.12997. PubMed PMID: 34169574; PubMed Central PMCID: PMC8447208.

112. Khondaker T, Qader MA, Gosh K, Chowdhury GN, Ferdous T, Afroz S, et al. Clinical Profile and Outcome of Covid -19 in Children with Pre-Existing Renal Disease. *Journal of Pediatric Nephrology*. 2020;9(1):1-6. doi: https://doi.org/10.22037/jpn.v9i1.33008.

113. Rivett L, Sridhar S, Sparkes D, Routledge M, Jones NK, Forrest S, et al. Screening of Healthcare Workers for Sars-Cov-2 Highlights the Role of Asymptomatic Carriage in Covid-19 Transmission. *Elife*. 2020;9. Epub 2020/05/12. doi: https://doi.org/10.7554/elife.58728. PubMed PMID: 32392129.

114. Treibel TA, Manisty C, Burton M, McKnight NA, Lambourne J, Augusto JB, et al. Covid-19: Pcr Screening of Asymptomatic Health-Care Workers at London Hospital. *Lancet*. 2020. doi: https://doi.org/10.1016/s0140-6736(20)31100-4.

115. Han X, Wei X, Alwalid O, Cao Y, Li Y, Wang L, et al. Severe Acute Respiratory Syndrome Coronavirus 2 among Asymptomatic Workers Screened for Work Resumption, China. *Emerg Infect Dis*. 2020;26(9). Epub 2020/06/20. doi: https://doi.org/10.3201/eid2609.201848. PubMed PMID: 32553070; PubMed Central PMCID: 7454110.

116. Lombardi A, Consonni D, Carugno M, Bozzi G, Mangioni D, Muscatello A, et al. Characteristics of 1573 Healthcare Workers Who Underwent Nasopharyngeal Swab Testing for Sars-Cov-2 in Milan, Lombardy, Italy. *Clin Microbiol Infect*. 2020;26(10):1413 e9- e13. Epub 2020/06/23. doi: https://doi.org/10.1016/j.cmi.2020.06.013. PubMed PMID: 32569835; PubMed Central PMCID: 7305713.

117. Cariani L, Orena BS, Ambrogi F, Gambazza S, Maraschini A, Dodaro A, et al. Time Length of Negativization and Cycle Threshold Values in 182 Healthcare Workers with Covid-19 in Milan, Italy: An Observational Cohort Study. *Int J Environ Res Public Health*. 2020;17(15). Epub 2020/07/29. doi: https://doi.org/10.3390/ijerph17155313. PubMed PMID: 32718008; PubMed Central PMCID: 7432921.

118. Stock AD, Bader ER, Cezayirli P, Inocencio J, Chalmers SA, Yassari R, et al. Covid-19 Infection among Healthcare Workers: Serological Findings Supporting Routine Testing. *Front Med (Lausanne)*. 2020;7:471. Epub 2020/09/26. doi: https://doi.org/10.3389/fmed.2020.00471. PubMed PMID: 32974370; PubMed Central PMCID: 7472984.

119. Malagon-Rojas J, Gomez-Rendon C, Parra EL, Almentero J, Palma R, Lopez R, et al. Sars-Cov-2 and Rt-Pcr in Asymptomatic Patients: Results of a Cohort of Workers at El Dorado International Airport in Bogota, 2020. *Biomedica*. 2020;40(Supl. 2):166-72. Epub 2020/11/06. doi: https://doi.org/10.7705/biomedica.5802. PubMed PMID: 33152200; PubMed Central PMCID: 7676845.

120. Letizia AG, Ramos I, Obla A, Goforth C, Weir DL, Ge Y, et al. Sars-Cov-2 Transmission among Marine Recruits During Quarantine. *N Engl J Med*. 2020;383(25):2407-16. Epub 2020/11/12. doi: https://doi.org/10.1056/nejmoa2029717. PubMed PMID: 33176093; PubMed Central PMCID: 7675690.

121. Mahajan NN, Kesarwani SN, Shinde SS, Nayak A, Modi DN, Mahale SD, et al. Co-Infection of Malaria and Dengue in Pregnant Women with Sars-Cov-2. *Int J Gynaecol Obstet*. 2020;151(3):459-62. Epub 2020/10/23. doi: https://doi.org/10.1002/ijgo.13415. PubMed PMID: 33090458; PubMed Central PMCID: 7611276.

122. Alshahrani MS, Alnimr A, Alnassri S, Alfarag S, Aljehani Y, Alabdali M. Prevalence of the Sars-Cov-2 Infection among Post-Quarantine Healthcare Workers. *J Multidiscip Healthc*. 2020;13:1927-36. Epub 2020/12/29. doi: https://doi.org/10.2147/jmdh.s279469. PubMed PMID: 33363380; PubMed Central PMCID: 7752650.

123. Hogan CA, Gombar S, Wang H, Roltgen K, Shi RZ, Holubar M, et al. Large-Scale Testing of Asymptomatic Healthcare Personnel for Severe Acute Respiratory Syndrome Coronavirus 2. *Emerg Infect Dis*. 2021;27(1). Epub 2020/12/02. doi: https://doi.org/10.3201/eid2701.203892. PubMed PMID: 33256889; PubMed Central PMCID: 7774535.

124. Ferreira VH, Chruscinski A, Kulasingam V, Pugh TJ, Dus T, Wouters B, et al. Prospective Observational Study and Serosurvey of Sars-Cov-2 Infection in Asymptomatic Healthcare Workers at a Canadian Tertiary Care Center. *PLoS One*. 2021;16(2):e0247258. Epub 2021/02/17. doi:

https://doi.org/10.1371/journal.pone.0247258. PubMed PMID: 33592074; PubMed Central PMCID: 7886177.

125. Tan-Loh J, Cheong BMK. A Descriptive Analysis of Clinical Characteristics of Covid-19 among Healthcare Workers in a District Specialist Hospital. *Med J Malaysia*. 2021;76(1):24-8. Epub 2021/01/30. doi: https://pubmed.ncbi.nlm.nih.gov/33510104/. PubMed PMID: 33510104.

126. Lalani T, Lee TK, Laing ED, Ritter A, Cooper E, Lee M, et al. Sars-Cov-2 Infections and Serologic Responses among Military Personnel Deployed on the Usns Comfort to New York City During the Covid-19 Pandemic. *Open Forum Infect Dis*. 2021;8(2):ofaa654. Epub 20210123. doi: https://doi.org/10.1093/ofid/ofaa654. PubMed PMID: 33553482; PubMed Central PMCID: PMC7856331.

127. Fakhim H, Nasri E, Aboutalebian S, Gholipour S, Nikaeen M, Vaezi A, et al. Asymptomatic Carriers of Coronavirus Disease 2019 among Healthcare Workers in Isfahan, Iran. *Future Virology*. 2021;16(2):93-8. doi: https://dx.doi.org/10.2217%2Ffvl-2020-0224.

128. Hall VJ, Foulkes S, Charlett A, Atti A, Monk EJM, Simmons R, et al. Sars-Cov-2 Infection Rates of Antibody-Positive Compared with Antibody-Negative Health-Care Workers in England: A Large, Multicentre, Prospective Cohort Study (Siren). *Lancet*. 2021;397(10283):1459-69. Epub 20210409. doi: https://doi.org/10.1016/s0140-6736(21)00675-9. PubMed PMID: 33844963; PubMed Central PMCID: PMC8040523.

129. Laws RL, Biraro S, Kirungi W, Gianetti B, Aibo D, Awor AC, et al. Coronavirus Disease 2019 (Covid-19) Mitigation Efforts and Testing During an in-Person Training Event-Uganda, 12-29 October 2020. *Clin Infect Dis*. 2021;73(Suppl 1):S42-s4. doi: https://doi.org/10.1093/cid/ciab331. PubMed PMID: 33912911; PubMed Central PMCID: PMC8135603.

130. Morgan SC, Aigner S, Anderson C, Belda-Ferre P, De Hoff P, Marotz CA, et al. Automated, Miniaturized, and Scalable Screening of Healthcare Workers, First Responders, and Students for Sars-Cov-2 in San Diego County. *medRxiv [Preprint]*. 2021:2021.06.25.21257885. doi: https://doi.org/10.1101/2021.06.25.21257885.

131. Luo L, Liu D, Liao X-l, Wu X-b, Jing Q-l, Zheng J-z, et al. Modes of Contact and Risk of Transmission in Covid-19 among Close Contacts. *bioRxiv [Preprint]*. 2020. doi: https://doi.org/10.1101/2020.03.24.20042606.

132. Chaw L, Koh WC, Jamaludin SA, Naing L, Alikhan MF, Wong J. Analysis of Sars-Cov-2 Transmission in Different Settings, Brunei. *Emerg Infect Dis*. 2020;26(11):2598-606. Epub 2020/10/10. doi: https://doi.org/10.3201/eid2611.202263. PubMed PMID: 33035448; PubMed Central PMCID: 7588541.

133. Bender JK, Brandl M, Höhle M, Buchholz U, Zeitlmann N. Analysis of Asymptomatic and Presymptomatic Transmission in Sars-Cov-2 Outbreak, Germany, 2020. *Emerging infectious diseases*. 2021;27(4):1159-63. Epub 2021/02/18. doi: https://dx.doi.org/10.3201%2Feid2704.204576. PubMed PMID: 33600301.

134. Wu P, Liu F, Chang Z, Lin Y, Ren M, Zheng C, et al. Assessing Asymptomatic, Presymptomatic, and Symptomatic Transmission Risk of Severe Acute Respiratory Syndrome Coronavirus 2. *Clin Infect Dis*. 2021;73(6):e1314-e20. doi: https://doi.org/10.1093/cid/ciab271. PubMed PMID: 33772573; PubMed Central PMCID: PMC8083716.
