# Supplementary material for: Occurrence and transmission potential of asymptomatic and presymptomatic SARS-CoV-2 infections: Update of a living systematic review and meta-analysis
Source: PLoS Med. 2022 May 26;19(5):e1003987. doi: 10.1371/journal.pmed.1003987 (PMC9135333; doi:10.1371/journal.pmed.1003987)
Supplement: S4 Fig — (PDF) [file pmed.1003987.s014.pdf]

**Published Jan '20 − Jun '20**				
Hoehl S [1]	1	2	0.50	[0.01; 0.99]
Chang L [2]	2	4	0.50	[0.07; 0.93]
Hijnen D [3]	1	11	0.09	[0.00; 0.41]
Schwierzeck V [4]	2	11	0.18	[0.02; 0.52]
Danis K [5]	1	12	0.08	[0.00; 0.38]
Zhang W [6]	4	12	0.33	[0.10; 0.65]
Böhmer MM [7]	1	16	0.06	[0.00; 0.30]
Dora AV [8]	6	16	0.38	[0.15; 0.65]
Han X [9]	17	17	1.00	[0.80; 1.00]
Bogani G [10]	10	19	0.53	[0.29; 0.76]
Cheng HY [11]	4	22	0.18	[0.05; 0.40]
Rivett L [12]	5	30	0.17	[0.06; 0.35]
Patel MC [13]	13	35	0.37	[0.21; 0.55]
Brandstetter S [14]	2	36	0.06	[0.01; 0.19]
Treibel TA [15]	12	44	0.27	[0.15; 0.43]
Pavli A [16]	7	46	0.15	[0.06; 0.29]
Arons MM [17]	3	47	0.06	[0.01; 0.18]
Wu J [18]	5	48	0.10	[0.03; 0.23]
London V [19]	22	68	0.32	[0.22; 0.45]
Park SY [20]	4	95	0.04	[0.01; 0.10]
Graham N [21]	46	126	0.37	[0.28; 0.46]
Wong J [22]	16	138	0.12	[0.07; 0.18]
Lombardi A [23]	17	139	0.12	[0.07; 0.19]
Andrikopoulou M [24]	46	158	0.29	[0.22; 0.37]
**Prediction interval**	.	.		**[0.01; 0.85]**
**Published Jul '20 − Dec '20**				
Tanacan A [25]	0	3	0.00	[0.00; 0.71]
Pirnay JP [26]	2	4	0.50	[0.07; 0.93]
Berghoff AS [27]	2	4	0.50	[0.07; 0.93]
AbdulRahman A [28]	3	6	0.50	[0.12; 0.88]
Corcorran MA [29]	3	8	0.38	[0.09; 0.76]
Viñuela MC [30]	8	8	1.00	[0.63; 1.00]
Bender WR [31]	6	8	0.75	[0.35; 0.97]
Yang N [32]	2	10	0.20	[0.03; 0.56]
Kutsuna S [33]	3	11	0.27	[0.06; 0.61]
Kirshblum SC [34]	2	12	0.17	[0.02; 0.48]
Romao VC [35]	0	14	0.00	[0.00; 0.23]
Wadhwa A [36]	6	17	0.35	[0.14; 0.62]
Balestrini S [37]	11	17	0.65	[0.38; 0.86]
Alshahrani MS [38]	12	18	0.67	[0.41; 0.87]
Stock AD [39]	6	19	0.32	[0.13; 0.57]
Yau K [40]	7	20	0.35	[0.15; 0.59]
Edelstein M [41]	4	20	0.20	[0.06; 0.44]
Redditt V [42]	3	24	0.12	[0.03; 0.32]
Harada S [Patients] [43]	8	24	0.33	[0.16; 0.55]
Park JH [44]	4	28	0.14	[0.04; 0.33]
Starling A [45]	29	31	0.94	[0.79; 0.99]
Malagón−Rojas J [46]	11	35	0.31	[0.17; 0.49]
Kittang BR [47]	0	40	0.00	[0.00; 0.09]
Yousaf AR [48]	0	47	0.00	[0.00; 0.08]
Harada S [Healthcare workers] [43]	25	49	0.51	[0.36; 0.66]
Letizia AG [49]	46	51	0.90	[0.79; 0.97]
Ladhani SN [Healthcare workers] [50]	26	53	0.49	[0.35; 0.63]
Migisha R [51]	20	54	0.37	[0.24; 0.51]
Aslam A [52]	38	65	0.58	[0.46; 0.71]
Plucinski MM [53]	14	66	0.21	[0.12; 0.33]
Marossy A [54]	46	67	0.69	[0.56; 0.79]
Njuguna H [55]	29	71	0.41	[0.29; 0.53]
Lavezzo E [56]	29	73	0.40	[0.28; 0.52]
Chamie G [57]	23	81	0.28	[0.19; 0.40]
Taylor J [Healthcare personnel] [58]	9	99	0.09	[0.04; 0.17]
Grijalva CG [59]	34	102	0.33	[0.24; 0.43]
Smith E [60]	42	103	0.41	[0.31; 0.51]
Ladhani SN [Residents] [4/]	46	105	0.44	[0.34; 0.54]
Wi YM [60]	7	111	0.06	[0.03; 0.13]
Taylor J [Residents] [58]	7	127	0.06	[0.02; 0.11]
Luo L2 [61]	8	127	0.06	[0.03; 0.12]
Hcini N [62]	87	137	0.64	[0.55; 0.72]
Shi SM [63]	21	146	0.14	[0.09; 0.21]
Eythorsson E [64]	25	178	0.14	[0.09; 0.20]
Cariani L [65]	32	182	0.18	[0.12; 0.24]
Shi Q [66]	60	183	0.33	[0.26; 0.40]
Al−Qahtani M [67]	116	188	0.62	[0.54; 0.69]
Pham QT [58]	89	208	0.43	[0.36; 0.50]
Adhikari EH [7/]	98	252	0.39	[0.33; 0.45]
Hurst JH [70]	87	293	0.30	[0.25; 0.35]
Cao S [71]	300	300	1.00	[0.99; 1.00]
Kennelly SP [Nursing home staff] [72]	97	395	0.25	[0.20; 0.29]
Ghinai I [73]	293	406	0.72	[0.68; 0.76]
Mahajan NN [74]	58	467	0.12	[0.10; 0.16]
Lee JY [75]	80	694	0.12	[0.09; 0.14]
Kennelly SP [Nursing home residents] [72]	193	710	0.27	[0.24; 0.31]
Almazeedi S [76]	473	1096	0.43	[0.40; 0.46]
Kasper MR [77]	572	1271	0.45	[0.42; 0.48]
White EM [68]	2194	5403	0.41	[0.39; 0.42]
**Prediction interval**	.	.		**[0.03; 0.91]**
**Published from Jan '21 onwards**				
Jeffery−Smith A [Staff] [8/]	2	3	0.67	[0.09; 0.99]
Cardillo L [Healthcare workers] [80]	2	4	0.50	[0.07; 0.93]
Fisher MJ [81]	3	4	0.75	[0.19; 0.99]
Vohra [Undergoing chemotherapy] [82]	2	4	0.50	[0.07; 0.93]
Pamplona J [83]	2	5	0.40	[0.05; 0.85]
Rauch JN [84]	2	6	0.33	[0.04; 0.78]
Garibaldi [Staff] [85]	0	8	0.00	[0.00; 0.37]
Lalani T [86]	4	8	0.50	[0.16; 0.84]
van Buul LW [Healthcare workers] [87]	0	9	0.00	[0.00; 0.34]
Ferreira VH [78]	5	9	0.56	[0.21; 0.86]
Vohra [Presurgical patients] [82]	10	10	1.00	[0.69; 1.00]
Theuring S [School students and staff] [9/]	2	10	0.20	[0.03; 0.56]
Varnell C [90]	5	10	0.50	[0.19; 0.81]
Haidar G [91]	9	11	0.82	[0.48; 0.98]
Morgan SC [92]	2	11	0.18	[0.02; 0.52]
Garibaldi [Residents] [85]	1	12	0.08	[0.00; 0.38]
Jeffery−Smith A [Residents] [8/]	6	13	0.46	[0.19; 0.75]
Orsi A [83]	13	14	0.93	[0.66; 1.00]
Theuring S [Household members] [8/]	0	14	0.00	[0.00; 0.23]
Hwang [94]	8	14	0.57	[0.29; 0.82]
Isoldi S [95]	4	15	0.27	[0.08; 0.55]
van Buul LW [Nursing home residents] [87]	3	16	0.19	[0.04; 0.46]
Maki G [96]	16	18	0.89	[0.65; 0.99]
Fakhim H [97]	14	21	0.67	[0.43; 0.85]
Green R [88]	22	22	1.00	[0.85; 1.00]
Martins Machado C [0/ /]	5	22	0.23	[0.08; 0.45]
Tian S [0/ 0]	7	24	0.29	[0.13; 0.51]
Khondaker T [0/ 1]	7	26	0.27	[0.12; 0.48]
Laws RL [0/ 2]	25	28	0.89	[0.72; 0.98]
Rincon A [0/ 3]	9	35	0.26	[0.12; 0.43]
Pizarro−Sánchez MS [104]	5	38	0.13	[0.04; 0.28]
Hogan CA [105]	20	38	0.53	[0.36; 0.69]
Tan−Loh J [106]	6	46	0.13	[0.05; 0.26]
Xie W [107]	4	53	0.08	[0.02; 0.18]
van den Besselaar JH [Healthcare workers] [1/ 8]	1	54	0.02	[0.00; 0.10]
Schmitt J [10/]	18	54	0.33	[0.21; 0.47]
Gettings JR [100]	31	55	0.56	[0.42; 0.70]
Patel MR [101]	43	55	0.78	[0.65; 0.88]
Meyers KJ [102]	67	86	0.78	[0.68; 0.86]
Jones A [103]	24	87	0.28	[0.19; 0.38]
Cardillo L [Patients] [80]	20	91	0.22	[0.14; 0.32]
Weinbergerova B [114]	6	105	0.06	[0.02; 0.12]
Paleker M [115]	41	112	0.37	[0.28; 0.46]
van den Besselaar JH [Residents] [1/ 8]	7	113	0.06	[0.03; 0.12]
Esteban I [116]	75	113	0.66	[0.57; 0.75]
Nunes MC [107]	14	115	0.12	[0.07; 0.20]
Turunen T [108]	23	127	0.18	[0.12; 0.26]
Blain H [11/]	14	161	0.09	[0.05; 0.14]
Say D [110]	61	171	0.36	[0.29; 0.43]
Beiting KJ [111]	50	172	0.29	[0.22; 0.36]
Uçkay, I [112]	71	175	0.41	[0.33; 0.48]
Hussain A [113]	54	266	0.20	[0.16; 0.26]
Marcus JE [124]	199	403	0.49	[0.44; 0.54]
Uysal E [125]	64	684	0.09	[0.07; 0.12]
Hall VJ [126]	293	1704	0.17	[0.15; 0.19]
Malhotra S [117]	1272	1729	0.74	[0.71; 0.76]
Abraha HE [118]	1935	2617	0.74	[0.72; 0.76]
Ren R [12/]	1612	3103	0.52	[0.50; 0.54]
**Prediction interval**	.	.		**[0.03; 0.91]**

References

1. Hoehl S, Rabenau H, Berger A, Kortenbusch M, Cinatl J, Bojkova D, et al. Evidence of Sars-Cov-2 Infection in Returning Travelers from Wuhan, China. *N Engl J Med*. 2020;382(13):1278-80. Epub 2020/02/19. doi: https://doi.org/10.1056/nejmc2001899. PubMed PMID: 32069388; PubMed Central PMCID: 7121749.

2. Chang L, Zhao L, Gong H, Wang L, Wang L. Severe Acute Respiratory Syndrome Coronavirus 2 Rna Detected in Blood Donations. *Emerg Infect Dis*. 2020;26(7):1631-3. Epub 2020/04/04. doi: https://doi.org/10.3201/eid2607.200839. PubMed PMID: 32243255; PubMed Central PMCID: 7323524.

3. Hijnen D, Marzano AV, Eyerich K, GeurtsvanKessel C, Gimenez-Arnau AM, Joly P, et al. Sars-Cov-2 Transmission from Presymptomatic Meeting Attendee, Germany. *Emerg Infect Dis*. 2020;26(8):1935-7. Epub 2020/05/12. doi: https://doi.org/10.3201/eid2608.201235. PubMed PMID: 32392125; PubMed Central PMCID: 7392453.

4. Schwierzeck V, Konig JC, Kuhn J, Mellmann A, Correa-Martinez CL, Omran H, et al. First Reported Nosocomial Outbreak of Severe Acute Respiratory Syndrome Coronavirus 2 in a Pediatric Dialysis Unit. *Clin Infect Dis*. 2021;72(2):265-70. Epub 2021/01/28. doi: https://doi.org/10.1093/cid/ciaa491. PubMed PMID: 33501962; PubMed Central PMCID: 7197625.

5. Danis K, Epaulard O, Benet T, Gaymard A, Campoy S, Bothelo-Nevers E, et al. Cluster of Coronavirus Disease 2019 (Covid-19) in the French Alps, 2020. *Clin Infect Dis*. 2020. Epub 2020/04/12. doi: https://doi.org/10.1093/cid/ciaa424. PubMed PMID: 32277759; PubMed Central PMCID: 7184384.

6. Zhang W, Cheng W, Luo L, Ma Y, Xu C, Qin P, et al. Secondary Transmission of Coronavirus Disease from Presymptomatic Persons, China. *Emerg Infect Dis*. 2020;26(8):1924-6. Epub 2020/05/27. doi: https://doi.org/10.3201/eid2608.201142. PubMed PMID: 32453686; PubMed Central PMCID: 7392433.

7. Bohmer MM, Buchholz U, Corman VM, Hoch M, Katz K, Marosevic DV, et al. Investigation of a Covid-19 Outbreak in Germany Resulting from a Single Travel-Associated Primary Case: A Case Series. *Lancet Infect Dis*. 2020;20(8):920-8. Epub 2020/05/19. doi: https://doi.org/10.1016/s1473-3099(20)30314-5. PubMed PMID: 32422201; PubMed Central PMCID: 7228725.

8. Dora AV, Winnett A, Jatt LP, Davar K, Watanabe M, Sohn L, et al. Universal and Serial Laboratory Testing for Sars-Cov-2 at a Long-Term Care Skilled Nursing Facility for Veterans - Los Angeles, California, 2020. *MMWR Morb Mortal Wkly Rep*. 2020;69(21):651-5. Epub 2020/05/29. doi: https://doi.org/10.15585/mmwr.mm6921e1. PubMed PMID: 32463809; PubMed Central PMCID: 7269604.

9. Han X, Wei X, Alwalid O, Cao Y, Li Y, Wang L, et al. Severe Acute Respiratory Syndrome Coronavirus 2 among Asymptomatic Workers Screened for Work Resumption, China. *Emerg Infect Dis*. 2020;26(9). Epub 2020/06/20. doi: https://doi.org/10.3201/eid2609.201848. PubMed PMID: 32553070; PubMed Central PMCID: 7454110.

10. Bogani G, Ditto A, Bosio S, Brusadelli C, Raspagliesi F. Cancer Patients Affected by Covid-19: Experience from Milan, Lombardy. *Gynecol Oncol*. 2020;158(2):262-5. Epub 2020/06/15. doi: https://doi.org/10.1016/j.ygyno.2020.06.161. PubMed PMID: 32534808; PubMed Central PMCID: 7286266.

90. Theuring S, Thielecke M, van Loon W, Hommes F, Hulso C, von der Haar A, et al. Sars-Cov-2 Infection and Transmission in School Settings During the Second Covid-19 Wave: A Cross-Sectional Study, Berlin, Germany, November 2020. *Euro Surveill*. 2021;26(34). Epub 2021/08/28. doi: https://doi.org/10.2807/1560-7917.es.2021.26.34.2100184. PubMed PMID: 34448448; PubMed Central PMCID: 8393892.

91. Varnell C, Jr., Harshman LA, Smith L, Liu C, Chen S, Al-Akash S, et al. Covid-19 in Pediatric Kidney Transplantation: The Improving Renal Outcomes Collaborative. *Am J Transplant*. 2021;21(8):2740-8. Epub 2021/01/17. doi: https://doi.org/10.1111/ajt.16501. PubMed PMID: 33452854; PubMed Central PMCID: 8013281.

92. Haidar G, Ayres A, King WC, McDonald M, Wells A, Mitchell SL, et al. Preprocedural Sars-Cov-2 Testing to Sustain Medically Needed Health Care Delivery During the Covid-19 Pandemic: A Prospective Observational Study. *Open Forum Infectious Diseases*. 2021;8(2). doi: https://doi.org/10.1093/ofid/ofab022.

93. Morgan SC, Aigner S, Anderson C, Belda-Ferre P, De Hoff P, Marotz CA, et al. Automated, Miniaturized, and Scalable Screening of Healthcare Workers, First Responders, and Students for Sars-Cov-2 in San Diego County. *medRxiv [Preprint]*. 2021:2021.06.25.21257885. doi: https://doi.org/10.1101/2021.06.25.21257885.

94. Orsi A, Domnich A, Pace VD, Ricucci V, Caligiuri P, Bottiglieri L, et al. Outbreak of Sars-Cov-2 Lineage 20i/501y.V1 in a Nursing Home Underlines the Crucial Role of Vaccination in Both Residents and Staff. *Vaccines*. 2021;9(6):591. PubMed PMID: https://doi.org/10.3390/vaccines9060591.

95. Hwang CE, Kussman A, Christle JW, Froelicher V, Wheeler MT, Moneghetti KJ. Findings from Cardiovascular Evaluation of National Collegiate Athletic Association Division I Collegiate Student-Athletes after Asymptomatic or Mildly Symptomatic Sars-Cov-2 Infection. *Clin J Sport Med*. 2022;32(2):103-7. doi: https://doi.org/10.1097/jsm.0000000000000954. PubMed PMID: 34173780.

96. Isoldi S, Mallardo S, Marcellino A, Bloise S, Dilillo A, Iorfida D, et al. The Comprehensive Clinic, Laboratory, and Instrumental Evaluation of Children with Covid-19: A 6-Months Prospective Study. *J Med Virol*. 2021;93(5):3122-32. Epub 20210216. doi: https://doi.org/10.1002/jmv.26871. PubMed PMID: 33570199; PubMed Central PMCID: PMC8014060.

97. Maki G, Abdollah F, Dabaja A, Suleyman G. 462. Prevalence and Outcome of Asymptomatic Procedural Patients with Covid-19 Infection. *Open Forum Infectious Diseases*. 2020;7(Supplement_1):S298-S. doi: https://doi.org/10.1093/ofid/ofaa439.655.

98. Fakhim H, Nasri E, Aboutalebian S, Gholipour S, Nikaeen M, Vaezi A, et al. Asymptomatic Carriers of Coronavirus Disease 2019 among Healthcare Workers in Isfahan, Iran. *Future Virology*. 2021;16(2):93-8. doi: https://dx.doi.org/10.2217%2Ffvl-2020-0224.

99. Green R, Tulloch JSP, Tunnah C, Coffey E, Lawrenson K, Fox A, et al. Covid-19 Testing in Outbreak-Free Care Homes: What Are the Public Health Benefits? *J Hosp Infect*. 2021;111:89-95. Epub 2021/01/17. doi: https://doi.org/10.1016/j.jhin.2020.12.024. PubMed PMID: 33453349; PubMed Central PMCID: 7837210.

100. Machado CM, Kerbauy MN, Colturato I, Arcuri LJ, dos Santos ACF, Silva FR, et al. Clinical Characteristics and Outcomes of Covid-19 in Hsct Recipients. *Blood*. 2020;136:19-. Epub 2021/08/03. doi: https://doi.org/10.1182/blood-2020-141959. PubMed PMID: PMC8330389.

References

1. Hoehl S, Rabenau H, Berger A, Kortenbusch M, Cinatl J, Bojkova D, et al. Evidence of Sars-Cov-2 Infection in Returning Travelers from Wuhan, China. *N Engl J Med*. 2020;382(13):1278-80. Epub 2020/02/19. doi: https://doi.org/10.1056/nejmc2001899. PubMed PMID: 32069388; PubMed Central PMCID: 7121749.

2. Chang L, Zhao L, Gong H, Wang L, Wang L. Severe Acute Respiratory Syndrome Coronavirus 2 Rna Detected in Blood Donations. *Emerg Infect Dis*. 2020;26(7):1631-3. Epub 2020/04/04. doi: https://doi.org/10.3201/eid2607.200839. PubMed PMID: 32243255; PubMed Central PMCID: 7323524.

3. Hijnen D, Marzano AV, Eyerich K, GeurtsvanKessel C, Gimenez-Arnau AM, Joly P, et al. Sars-Cov-2 Transmission from Presymptomatic Meeting Attendee, Germany. *Emerg Infect Dis*. 2020;26(8):1935-7. Epub 2020/05/12. doi: https://doi.org/10.3201/eid2608.201235. PubMed PMID: 32392125; PubMed Central PMCID: 7392453.

4. Schwierzeck V, Konig JC, Kuhn J, Mellmann A, Correa-Martinez CL, Omran H, et al. First Reported Nosocomial Outbreak of Severe Acute Respiratory Syndrome Coronavirus 2 in a Pediatric Dialysis Unit. *Clin Infect Dis*. 2021;72(2):265-70. Epub 2021/01/28. doi: https://doi.org/10.1093/cid/ciaa491. PubMed PMID: 33501962; PubMed Central PMCID: 7197625.

5. Danis K, Epaulard O, Benet T, Gaymard A, Campoy S, Bothelo-Nevers E, et al. Cluster of Coronavirus Disease 2019 (Covid-19) in the French Alps, 2020. *Clin Infect Dis*. 2020. Epub 2020/04/12. doi: https://doi.org/10.1093/cid/ciaa424. PubMed PMID: 32277759; PubMed Central PMCID: 7184384.

6. Zhang W, Cheng W, Luo L, Ma Y, Xu C, Qin P, et al. Secondary Transmission of Coronavirus Disease from Presymptomatic Persons, China. *Emerg Infect Dis*. 2020;26(8):1924-6. Epub 2020/05/27. doi: https://doi.org/10.3201/eid2608.201142. PubMed PMID: 32453686; PubMed Central PMCID: 7392433.

7. Bohmer MM, Buchholz U, Corman VM, Hoch M, Katz K, Marosevic DV, et al. Investigation of a Covid-19 Outbreak in Germany Resulting from a Single Travel-Associated Primary Case: A Case Series. *Lancet Infect Dis*. 2020;20(8):920-8. Epub 2020/05/19. doi: https://doi.org/10.1016/s1473-3099(20)30314-5. PubMed PMID: 32422201; PubMed Central PMCID: 7228725.

8. Dora AV, Winnett A, Jatt LP, Davar K, Watanabe M, Sohn L, et al. Universal and Serial Laboratory Testing for Sars-Cov-2 at a Long-Term Care Skilled Nursing Facility for Veterans - Los Angeles, California, 2020. *MMWR Morb Mortal Wkly Rep*. 2020;69(21):651-5. Epub 2020/05/29. doi: https://doi.org/10.15585/mmwr.mm6921e1. PubMed PMID: 32463809; PubMed Central PMCID: 7269604.

9. Han X, Wei X, Alwalid O, Cao Y, Li Y, Wang L, et al. Severe Acute Respiratory Syndrome Coronavirus 2 among Asymptomatic Workers Screened for Work Resumption, China. *Emerg Infect Dis*. 2020;26(9). Epub 2020/06/20. doi: https://doi.org/10.3201/eid2609.201848. PubMed PMID: 32553070; PubMed Central PMCID: 7454110.

10. Bogani G, Ditto A, Bosio S, Brusadelli C, Raspagliesi F. Cancer Patients Affected by Covid-19: Experience from Milan, Lombardy. *Gynecol Oncol*. 2020;158(2):262-5. Epub 2020/06/15. doi: https://doi.org/10.1016/j.ygyno.2020.06.161. PubMed PMID: 32534808; PubMed Central PMCID: 7286266.

11. Cheng HY, Jian SW, Liu DP, Ng TC, Huang WT, Lin HH, et al. Contact Tracing Assessment of Covid-19 Transmission Dynamics in Taiwan and Risk at Different Exposure Periods before and after Symptom Onset. *JAMA Intern Med*. 2020;180(9):1156-63. Epub 2020/05/02. doi: https://doi.org/10.1001/jamainternmed.2020.2020 PubMed PMID: 32356867; PubMed Central PMCID: 7195694.

12. Rivett L, Sridhar S, Sparkes D, Routledge M, Jones NK, Forrest S, et al. Screening of Healthcare Workers for Sars-Cov-2 Highlights the Role of Asymptomatic Carriage in Covid-19 Transmission. *Elife*. 2020;9. Epub 2020/05/12. doi: https://doi.org/10.7554/elife.58728. PubMed PMID: 32392129.

13. Patel MC, Chaisson LH, Borgetti S, Burdsall D, Chugh RK, Hoff CR, et al. Asymptomatic Sars-Cov-2 Infection and Covid-19 Mortality During an Outbreak Investigation in a Skilled Nursing Facility. *Clin Infect Dis*. 2020;71(11):2920-6. Epub 2020/06/18. doi: https://doi.org/10.1093/cid/ciaa763. PubMed PMID: 32548628; PubMed Central PMCID: 7337684.

14. Brandstetter S, Roth S, Harner S, Buntrock-Dopke H, Toncheva AA, Borchers N, et al. Symptoms and Immunoglobulin Development in Hospital Staff Exposed to a Sars-Cov-2 Outbreak. *Pediatr Allergy Immunol*. 2020;31(7):841-7. Epub 2020/05/16. doi: https://doi.org/10.1111/pai.13278. PubMed PMID: 32413201.

15. Treibel TA, Manisty C, Burton M, McKnight NA, Lambourne J, Augusto JB, et al. Covid-19: Pcr Screening of Asymptomatic Health-Care Workers at London Hospital. *Lancet*. 2020. doi: https://doi.org/10.1016/s0140-6736(20)31100-4.

16. Pavli A, Smeti P, Papadima K, Andreopoulou A, Hadjianastasiou S, Triantafillou E, et al. A Cluster of Covid-19 in Pilgrims to Israel. *J Travel Med*. 2020;27(5). Epub 2020/06/26. doi: https://doi.org/10.1093/jtm/taaa102. PubMed PMID: 32584403; PubMed Central PMCID: 7337872.

17. Arons MM, Hatfield KM, Reddy SC, Kimball A, James A, Jacobs JR, et al. Presymptomatic Sars-Cov-2 Infections and Transmission in a Skilled Nursing Facility. *N Engl J Med*. 2020;382(22):2081-90. Epub 2020/04/25. doi: https://doi.org/10.1056/NEJMoa2008457. PubMed PMID: 32329971; PubMed Central PMCID: 7200056.

18. Wu J, Huang Y, Tu C, Bi C, Chen Z, Luo L, et al. Household Transmission of Sars-Cov-2, Zhuhai, China, 2020. *Clin Infect Dis*. 2020;71(16):2099-108. Epub 2020/05/12. doi: https://doi.org/10.1093/cid/ciaa557. PubMed PMID: 32392331; PubMed Central PMCID: 7239243.

19. London V, McLaren R, Jr., Atallah F, Cepeda C, McCalla S, Fisher N, et al. The Relationship between Status at Presentation and Outcomes among Pregnant Women with Covid-19. *Am J Perinatol*. 2020;37(10):991-4. Epub 2020/05/20. doi: https://doi.org/10.1055/s-0040-1712164. PubMed PMID: 32428964; PubMed Central PMCID: 7416203.

20. Park SY, Kim YM, Yi S, Lee S, Na BJ, Kim CB, et al. Coronavirus Disease Outbreak in Call Center, South Korea. *Emerg Infect Dis*. 2020;26(8):1666-70. Epub 2020/04/24. doi: https://doi.org/10.3201/eid2608.201274. PubMed PMID: 32324530; PubMed Central PMCID: 7392450.

21. Graham NSN, Junghans C, Downes R, Sendall C, Lai H, McKirdy A, et al. Sars-Cov-2 Infection, Clinical Features and Outcome of Covid-19 in United Kingdom Nursing Homes. *J*

Infect. 2020;81(3):411-9. Epub 2020/06/07. doi: https://doi.org/10.1016/j.jinf.2020.05.073. PubMed PMID: 32504743; PubMed Central PMCID: 7836316.

22. Wong J, Abdul Aziz ABZ, Chaw L, Mahamud A, Griffith MM, Ying-Ru LO, et al. High Proportion of Asymptomatic and Presymptomatic Covid-19 Infections in Travelers and Returning Residents to Brunei. *J Travel Med*. 2020. doi: https://doi.org/10.1093/jtm/taaa066.

23. Lombardi A, Consonni D, Carugno M, Bozzi G, Mangioni D, Muscatello A, et al. Characteristics of 1573 Healthcare Workers Who Underwent Nasopharyngeal Swab Testing for Sars-Cov-2 in Milan, Lombardy, Italy. *Clin Microbiol Infect*. 2020;26(10):1413 e9- e13. Epub 2020/06/23. doi: https://doi.org/10.1016/j.cmi.2020.06.013. PubMed PMID: 32569835; PubMed Central PMCID: 7305713.

24. Andrikopoulou M, Madden N, Wen T, Aubey JJ, Aziz A, Baptiste CD, et al. Symptoms and Critical Illness among Obstetric Patients with Coronavirus Disease 2019 (Covid-19) Infection. *Obstet Gynecol*. 2020;136(2):291-9. Epub 2020/05/28. doi: https://doi.org/10.1097/aog.0000000000003996. PubMed PMID: 32459701.

25. Tanacan A, Erol SA, Turgay B, Anuk AT, Secen EI, Yegin GF, et al. The Rate of Sars-Cov-2 Positivity in Asymptomatic Pregnant Women Admitted to Hospital for Delivery: Experience of a Pandemic Center in Turkey. *Eur J Obstet Gynecol Reprod Biol*. 2020;253:31-4. Epub 2020/08/09. doi: https://doi.org/10.1016/j.ejogrb.2020.07.051. PubMed PMID: 32763728; PubMed Central PMCID: 7390745.

26. Pirnay JP, Selhorst P, Cochez C, Petrillo M, Claes V, Van der Beken Y, et al. Study of a Sars-Cov-2 Outbreak in a Belgian Military Education and Training Center in Maradi, Niger. *Viruses*. 2020;12(9). Epub 2020/09/02. doi: https://doi.org/10.3390/v12090949. PubMed PMID: 32867108; PubMed Central PMCID: 7552053.

27. Berghoff AS, Gansterer M, Bathke AC, Trutschnig W, Hungerlander P, Berger JM, et al. Sars-Cov-2 Testing in Patients with Cancer Treated at a Tertiary Care Hospital During the Covid-19 Pandemic. *J Clin Oncol*. 2020;38(30):3547-54. Epub 2020/08/17. doi: https://doi.org/10.1200/jco.20.01442. PubMed PMID: 32795227; PubMed Central PMCID: 7571795.

28. AbdulRahman A, AlAli S, Yaghi O, Shabaan M, Otoom S, Atkin SL, et al. Covid-19 and Sickle Cell Disease in Bahrain. *Int J Infect Dis*. 2020;101:14-6. Epub 2020/09/28. doi: https://doi.org/10.1016/j.ijid.2020.09.1433. PubMed PMID: 32980556; PubMed Central PMCID: 7833176.

29. Corcorran MA, Olin S, Rani G, Nasenbeny K, Constantino-Shor C, Holmes C, et al. Prolonged Persistence of Pcr-Detectable Virus During an Outbreak of Sars-Cov-2 in an Inpatient Geriatric Psychiatry Unit in King County, Washington. *Am J Infect Control*. 2021;49(3):293-8. Epub 2020/08/23. doi: https://doi.org/10.1016/j.ajic.2020.08.025. PubMed PMID: 32827597; PubMed Central PMCID: 7438365.

30. Vinuela MC, De Leon-Luis JA, Alonso R, Catalan P, Lizarraga S, Munoz P, et al. Sars-Cov-2 Screening of Asymptomatic Women Admitted for Delivery Must Be Performed with a Combination of Microbiological Techniques: An Observational Study. *Rev Esp Quimioter*. 2020;33(6):415-21. Epub 2020/09/19. doi: https://doi.org/10.37201/req/088.2020. PubMed PMID: 32945157; PubMed Central PMCID: 7712338.

31. Bender WR, Hirshberg A, Coutifaris P, Acker AL, Srinivas SK. Universal Testing for Severe Acute Respiratory Syndrome Coronavirus 2 in 2 Philadelphia Hospitals: Carrier Prevalence and Symptom Development over 2 Weeks. *Am J Obstet Gynecol MFM*.

2020;2(4):100226. Epub 2020/09/22. doi: https://doi.org/10.1016/j.ajogmf.2020.100226. PubMed PMID: 32954248; PubMed Central PMCID: 7485442.

32. Yang N, Shen Y, Shi C, Ma AHY, Zhang X, Jian X, et al. In-Flight Transmission Cluster of Covid-19: A Retrospective Case Series. *Infect Dis (Lond)*. 2020;52(12):891-901. Epub 2020/08/01. doi: https://doi.org/10.1080/23744235.2020.1800814. PubMed PMID: 32735163.

33. Kutsuna S, Suzuki T, Hayakawa K, Tsuzuki S, Asai Y, Suzuki T, et al. Sars-Cov-2 Screening Test for Japanese Returnees from Wuhan, China, January 2020. *Open Forum Infect Dis*. 2020;7(7):ofaa243. Epub 2020/08/06. doi: https://doi.org/10.1093/ofid/ofaa243. PubMed PMID: 32754627; PubMed Central PMCID: 7337761.

34. Kirshblum SC, DeLauter G, Lopreiato MC, Pomeranz B, Dawson A, Hammerman S, et al. Screening Testing for Sars-Cov-2 Upon Admission to Rehabilitation Hospitals in a High Covid-19 Prevalence Community. *PM R*. 2020;12(10):1009-14. Epub 2020/07/24. doi: https://doi.org/10.1002/pmrj.12454. PubMed PMID: 32700434; PubMed Central PMCID: 7404891.

35. Romao VC, Oliveira-Ramos F, Cruz-Machado AR, Martins P, Barreira S, Silva-Dinis J, et al. A Covid-19 Outbreak in a Rheumatology Department Upon the Early Days of the Pandemic. *Front Med (Lausanne)*. 2020;7:576162. Epub 2020/10/27. doi: https://doi.org/10.3389/fmed.2020.576162. PubMed PMID: 33102507; PubMed Central PMCID: 7546334.

36. Wadhwa A, Fisher KA, Silver R, Koh M, Arons MM, Miller DA, et al. Identification of Presymptomatic and Asymptomatic Cases Using Cohort-Based Testing Approaches at a Large Correctional Facility-Chicago, Illinois, USA, May 2020. *Clin Infect Dis*. 2021;72(5):e128-e35. Epub 2020/12/04. doi: https://doi.org/10.1093/cid/ciaa1802. PubMed PMID: 33270101; PubMed Central PMCID: 7799274.

37. Balestrini S, Koepp MJ, Gandhi S, Rickman HM, Shin GY, Houlihan CF, et al. Clinical Outcomes of Covid-19 in Long-Term Care Facilities for People with Epilepsy. *Epilepsy Behav*. 2021;115:107602. Epub 2020/12/07. doi: https://doi.org/10.1016/j.yebeh.2020.107602. PubMed PMID: 33279440; PubMed Central PMCID: 7643621.

38. Alshahrani MS, Alnimr A, Alnassri S, Alfarag S, Aljehani Y, Alabdali M. Prevalence of the Sars-Cov-2 Infection among Post-Quarantine Healthcare Workers. *J Multidiscip Healthc*. 2020;13:1927-36. Epub 2020/12/29. doi: https://doi.org/10.2147/jmdh.s279469. PubMed PMID: 33363380; PubMed Central PMCID: 7752650.

39. Stock AD, Bader ER, Cezayirli P, Inocencio J, Chalmers SA, Yassari R, et al. Covid-19 Infection among Healthcare Workers: Serological Findings Supporting Routine Testing. *Front Med (Lausanne)*. 2020;7:471. Epub 2020/09/26. doi: https://doi.org/10.3389/fmed.2020.00471. PubMed PMID: 32974370; PubMed Central PMCID: 7472984.

40. Yau K, Muller MP, Lin M, Siddiqui N, Neskovic S, Shokar G, et al. Covid-19 Outbreak in an Urban Hemodialysis Unit. *Am J Kidney Dis*. 2020;76(5):690-5 e1. Epub 2020/07/19. doi: https://doi.org/10.1053/j.ajkd.2020.07.001. PubMed PMID: 32681983; PubMed Central PMCID: 7362862.

41. Edelstein M, Obi C, Chand M, Hopkins S, Brown K, Ramsay M. Sars-Cov-2 Infection in London, England: Changes to Community Point Prevalence around Lockdown Time, March-May 2020. *J Epidemiol Community Health*. 2021;75(2):185-8. Epub 2020/10/03. doi:

https://doi.org/10.1136/jech-2020-214730. PubMed PMID: 33004659; PubMed Central PMCID: 7815898.

42. Redditt V, Wright V, Rashid M, Male R, Bogoch I. Outbreak of Sars-Cov-2 Infection at a Large Refugee Shelter in Toronto, April 2020: A Clinical and Epidemiologic Descriptive Analysis. *CMAJ Open*. 2020;8(4):E819-E24. Epub 2020/12/10. doi: https://doi.org/10.9778/cmajo.20200165. PubMed PMID: 33293331; PubMed Central PMCID: 7743908.

43. Harada S, Uno S, Ando T, Iida M, Takano Y, Ishibashi Y, et al. Control of a Nosocomial Outbreak of Covid-19 in a University Hospital. *Open Forum Infect Dis*. 2020;7(12):ofaa512. Epub 2020/12/18. doi: https://doi.org/10.1093/ofid/ofaa512. PubMed PMID: 33330740; PubMed Central PMCID: 7665726.

44. Park JH, Jang JH, Lee K, Yoo SJ, Shin H. Covid-19 Outbreak and Presymptomatic Transmission in Pilgrim Travelers Who Returned to Korea from Israel. *J Korean Med Sci*. 2020;35(48):e424. Epub 2020/12/15. doi: https://doi.org/10.3346/jkms.2020.35.e424. PubMed PMID: 33316860; PubMed Central PMCID: 7735917.

45. Starling A, White E, Showell D, Wyllie D, Kapadia S, Balakrishnan R. Whole Care Home Testing for Covid-19 in a Local Authority Area in the United Kingdom. *medRxiv [Preprint]*. 2020. doi: https://doi.org/10.1101/2020.08.06.20162859.

46. Malagon-Rojas J, Gomez-Rendon C, Parra EL, Almentero J, Palma R, Lopez R, et al. Sars-Cov-2 and Rt-Pcr in Asymptomatic Patients: Results of a Cohort of Workers at El Dorado International Airport in Bogota, 2020. *Biomedica*. 2020;40(Supl. 2):166-72. Epub 2020/11/06. doi: https://doi.org/10.7705/biomedica.5802. PubMed PMID: 33152200; PubMed Central PMCID: 7676845.

47. Kittang BR, Hofacker SV, Solheim SP, Kruger K, Loland KK, Jansen K. Outbreak of Covid-19 at Three Nursing Homes in Bergen. *Tidsskr Nor Laegeforen*. 2020;140(11). Epub 2020/08/21. doi: https://doi.org/10.4045/tidsskr.20.0405. PubMed PMID: 32815356.

48. Yousaf AR, Duca LM, Chu V, Reses HE, Fajans M, Rabold EM, et al. A Prospective Cohort Study in Nonhospitalized Household Contacts with Severe Acute Respiratory Syndrome Coronavirus 2 Infection: Symptom Profiles and Symptom Change over Time. *Clin Infect Dis*. 2021;73(7):e1841-e9. Epub 2020/07/29. doi: https://doi.org/10.1093/cid/ciaa1072. PubMed PMID: 32719874; PubMed Central PMCID: 7454397.

49. Letizia AG, Ramos I, Obla A, Goforth C, Weir DL, Ge Y, et al. Sars-Cov-2 Transmission among Marine Recruits During Quarantine. *N Engl J Med*. 2020;383(25):2407-16. Epub 2020/11/12. doi: https://doi.org/10.1056/nejmoa2029717. PubMed PMID: 33176093; PubMed Central PMCID: 7675690.

50. Ladhani SN, Jeffery-Smith A, Patel M, Janarthanan R, Fok J, Crawley-Boevey E, et al. High Prevalence of Sars-Cov-2 Antibodies in Care Homes Affected by Covid-19: Prospective Cohort Study, England. *EClinicalMedicine*. 2020;28:100597. Epub 2020/11/12. doi: https://doi.org/10.1016/j.eclinm.2020.100597. PubMed PMID: 33173854; PubMed Central PMCID: 7644437.

51. Migisha R, Kwesiga B, Mirembe BB, Amanya G, Kabwama SN, Kadobera D, et al. Early Cases of Sars-Cov-2 Infection in Uganda: Epidemiology and Lessons Learned from Risk-Based Testing Approaches - March-April 2020. *Global Health*. 2020;16(1):114. Epub 2020/11/27. doi: https://doi.org/10.1186/s12992-020-00643-7. PubMed PMID: 33239041; PubMed Central PMCID: 7686950.

52. Aslam A, Singh J, Robilotti E, Chow K, Bist T, Reidy-Lagunes D, et al. Severe Acute Respiratory Syndrome Coronavirus 2 Surveillance and Exposure in the Perioperative Setting with Universal Testing and Personal Protective Equipment Policies. *Clin Infect Dis*. 2021;73(9):e3013-e8. Epub 2020/10/23. doi: https://doi.org/10.1093/cid/ciaa1607. PubMed PMID: 33090210; PubMed Central PMCID: 7665395.

53. Plucinski MM, Wallace M, Uehara A, Kurbatova EV, Tobolowsky FA, Schneider ZD, et al. Coronavirus Disease 2019 (Covid-19) in Americans Aboard the Diamond Princess Cruise Ship. *Clin Infect Dis*. 2021;72(10):e448-e57. Epub 2020/08/14. doi: https://doi.org/10.1093/cid/ciaa1180. PubMed PMID: 32785683; PubMed Central PMCID: 7454359.

54. Marossy A, Rakowicz S, Bhan A, Noon S, Rees A, Virk M, et al. A Study of Universal Severe Acute Respiratory Syndrome Coronavirus 2 Rna Testing among Residents and Staff in a Large Group of Care Homes in South London. *J Infect Dis*. 2021;223(3):381-8. Epub 2020/09/06. doi: https://doi.org/10.1093/infdis/jiaa565. PubMed PMID: 32889532; PubMed Central PMCID: 7499645.

55. Njuguna H, Wallace M, Simonson S, Tobolowsky FA, James AE, Bordelon K, et al. Serial Laboratory Testing for Sars-Cov-2 Infection among Incarcerated and Detained Persons in a Correctional and Detention Facility - Louisiana, April-May 2020. *MMWR Morb Mortal Wkly Rep*. 2020;69(26):836-40. Epub 2020/07/03. doi: https://doi.org/10.15585/mmwr.mm6926e2. PubMed PMID: 32614816; PubMed Central PMCID: 7332096.

56. Lavezzo E, Franchin E, Ciavarella C, Cuomo-Dannenburg G, Barzon L, Del Vecchio C, et al. Suppression of a Sars-Cov-2 Outbreak in the Italian Municipality of Vo'. *Nature*. 2020;584(7821):425-9. Epub 2020/07/01. doi: https://doi.org/10.1038/s41586-020-2488-1. PubMed PMID: 32604404.

57. Chamie G, Marquez C, Crawford E, Peng J, Petersen M, Schwab D, et al. Community Transmission of Severe Acute Respiratory Syndrome Coronavirus 2 Disproportionately Affects the Latinx Population During Shelter-in-Place in San Francisco. *Clin Infect Dis*. 2021;73(Suppl 2):S127-S35. Epub 2020/08/22. doi: https://doi.org/10.1093/cid/ciaa1234. PubMed PMID: 32821935; PubMed Central PMCID: 7499499.

58. Taylor J, Carter RJ, Lehnertz N, Kazazian L, Sullivan M, Wang X, et al. Serial Testing for Sars-Cov-2 and Virus Whole Genome Sequencing Inform Infection Risk at Two Skilled Nursing Facilities with Covid-19 Outbreaks - Minnesota, April-June 2020. *MMWR Morb Mortal Wkly Rep*. 2020;69(37):1288-95. Epub 2020/09/24. doi: https://doi.org/10.15585/mmwr.mm6937a3. PubMed PMID: 32966272; PubMed Central PMCID: 7498172.

59. Grijalva CG, Rolfes MA, Zhu Y, McLean HQ, Hanson KE, Belongia EA, et al. Transmission of Sars-Cov-2 Infections in Households - Tennessee and Wisconsin, April-September 2020. *MMWR Morb Mortal Wkly Rep*. 2020;69(44):1631-4. Epub 2020/11/06. doi: http://dx.doi.org/10.15585/mmwr.mm6944e1. PubMed PMID: 33151916; PubMed Central PMCID: 7643897.

60. Smith E, Aldus CF, Brainard J, Dunham S, Hunter PR, Steel N, et al. Testing for Sars-Cov-2 in Care Home Staff and Residents in English Care Homes: A Service Evaluation. *medRxiv [Preprint]*. 2020. doi: https://doi.org/10.1101/2020.08.04.20165928.

61. Wi YM, Lim SJ, Kim SH, Lim S, Lee SJ, Ryu BH, et al. Response System for and Epidemiological Features of Covid-19 in Gyeongsangnam-Do Province in South Korea. *Clin*

Infect Dis. 2021;72(4):661-7. Epub 2020/07/17. doi: https://doi.org/10.1093/cid/ciaa967. PubMed PMID: 32672789; PubMed Central PMCID: 7454481.

62. Luo L, Liu D, Liao X, Wu X, Jing Q, Zheng J, et al. Contact Settings and Risk for Transmission in 3410 Close Contacts of Patients with Covid-19 in Guangzhou, China : A Prospective Cohort Study. *Ann Intern Med*. 2020;173(11):879-87. Epub 2020/08/14. doi: https://doi.org/10.7326/m20-2671. PubMed PMID: 32790510; PubMed Central PMCID: 7506769.

63. Hcini N, Maamri F, Picone O, Carod JF, Lambert V, Mathieu M, et al. Maternal, Fetal and Neonatal Outcomes of Large Series of Sars-Cov-2 Positive Pregnancies in Peripartum Period: A Single-Center Prospective Comparative Study. *Eur J Obstet Gynecol Reprod Biol*. 2021;257:11-8. Epub 2020/12/15. doi: https://doi.org/10.1016/j.ejogrb.2020.11.068. PubMed PMID: 33310656; PubMed Central PMCID: 7705341.

64. Shi SM, Bakaev I, Chen H, Travison TG, Berry SD. Risk Factors, Presentation, and Course of Coronavirus Disease 2019 in a Large, Academic Long-Term Care Facility. *J Am Med Dir Assoc*. 2020;21(10):1378-83.e1. Epub 20200825. doi: https://doi.org/10.1016/j.jamda.2020.08.027. PubMed PMID: 32981664; PubMed Central PMCID: 7447263.

65. Eythorsson E, Helgason D, Ingvarsson RF, Bjornsson HK, Olafsdottir LB, Bjarnadottir V, et al. Clinical Spectrum of Coronavirus Disease 2019 in Iceland: Population Based Cohort Study. *BMJ*. 2020;371:m4529. Epub 2020/12/04. doi: https://doi.org/10.1136/bmj.m4529. PubMed PMID: 33268329; PubMed Central PMCID: 7708618.

66. Cariani L, Orena BS, Ambrogi F, Gambazza S, Maraschini A, Dodaro A, et al. Time Length of Negativization and Cycle Threshold Values in 182 Healthcare Workers with Covid-19 in Milan, Italy: An Observational Cohort Study. *Int J Environ Res Public Health*. 2020;17(15). Epub 2020/07/29. doi: https://doi.org/10.3390/ijerph17155313. PubMed PMID: 32718008; PubMed Central PMCID: 7432921.

67. Shi Q, Hu Y, Peng B, Tang XJ, Wang W, Su K, et al. Effective Control of Sars-Cov-2 Transmission in Wanzhou, China. *Nat Med*. 2021;27(1):86-93. Epub 20201130. doi: https://doi.org/10.1038/s41591-020-01178-5. PubMed PMID: 33257893.

68. Al-Qahtani M, AlAli S, AbdulRahman A, Salman Alsayyad A, Otoom S, Atkin SL. The Prevalence of Asymptomatic and Symptomatic Covid-19 in a Cohort of Quarantined Subjects. *Int J Infect Dis*. 2021;102:285-8. Epub 2020/11/07. doi: https://doi.org/10.1016/j.ijid.2020.10.091. PubMed PMID: 33157290; PubMed Central PMCID: 7607262.

69. Thai PQ, Rabaa MA, Luong DH, Tan DQ, Quang TD, Quach HL, et al. The First 100 Days of Severe Acute Respiratory Syndrome Coronavirus 2 (Sars-Cov-2) Control in Vietnam. *Clin Infect Dis*. 2021;72(9):e334-e42. Epub 2020/08/02. doi: https://doi.org/10.1093/cid/ciaa1130. PubMed PMID: 32738143; PubMed Central PMCID: 7454342.

70. Adhikari EH, Moreno W, Zofkie AC, MacDonald L, McIntire DD, Collins RRJ, et al. Pregnancy Outcomes among Women with and without Severe Acute Respiratory Syndrome Coronavirus 2 Infection. *JAMA Netw Open*. 2020;3(11):e2029256. Epub 2020/11/20. doi: https://doi.org/10.1001/jamanetworkopen.2020.29256. PubMed PMID: 33211113; PubMed Central PMCID: 7677755.

71. Hurst JH, Heston SM, Chambers HN, Cunningham HM, Price MJ, Suarez L, et al. Severe Acute Respiratory Syndrome Coronavirus 2 Infections among Children in the

Biospecimens from Respiratory Virus-Exposed Kids (Brave Kids) Study. *Clinical Infectious Diseases*. 2021;73(9):e2875-e82. doi: https://doi.org/10.1093/cid/ciaa1693.

72. Cao S, Gan Y, Wang C, Bachmann M, Wei S, Gong J, et al. Post-Lockdown Sars-Cov-2 Nucleic Acid Screening in Nearly Ten Million Residents of Wuhan, China. *Nat Commun*. 2020;11(1):5917. Epub 2020/11/22. doi: https://doi.org/10.1038/s41467-020-19802-w. PubMed PMID: 33219229; PubMed Central PMCID: 7679396.

73. Kennelly SP, Dyer AH, Noonan C, Martin R, Kennelly SM, Martin A, et al. Asymptomatic Carriage Rates and Case Fatality of Sars-Cov-2 Infection in Residents and Staff in Irish Nursing Homes. *Age Ageing*. 2021;50(1):49-54. Epub 2020/09/29. doi: https://doi.org/10.1093/ageing/afaa220. PubMed PMID: 32986806; PubMed Central PMCID: 7543256.

74. Ghinai I, Davis ES, Mayer S, Toews KA, Huggett TD, Snow-Hill N, et al. Risk Factors for Severe Acute Respiratory Syndrome Coronavirus 2 Infection in Homeless Shelters in Chicago, Illinois-March-May, 2020. *Open Forum Infect Dis*. 2020;7(11):ofaa477. Epub 2020/12/03. doi: https://doi.org/10.1093/ofid/ofaa477. PubMed PMID: 33263069; PubMed Central PMCID: 7665740.

75. Mahajan NN, Kesarwani SN, Shinde SS, Nayak A, Modi DN, Mahale SD, et al. Co-Infection of Malaria and Dengue in Pregnant Women with Sars-Cov-2. *Int J Gynaecol Obstet*. 2020;151(3):459-62. Epub 2020/10/23. doi: https://doi.org/10.1002/ijgo.13415. PubMed PMID: 33090458; PubMed Central PMCID: 7611276.

76. Lee JY, Hong SW, Hyun M, Park JS, Lee JH, Suh YS, et al. Epidemiological and Clinical Characteristics of Coronavirus Disease 2019 in Daegu, South Korea. *Int J Infect Dis*. 2020;98:462-6. Epub 2020/07/24. doi: https://doi.org/10.1016/j.ijid.2020.07.017. PubMed PMID: 32702415; PubMed Central PMCID: 7371586.

77. Almazeedi S, Al-Youha S, Jamal MH, Al-Haddad M, Al-Muhaini A, Al-Ghimlas F, et al. Characteristics, Risk Factors and Outcomes among the First Consecutive 1096 Patients Diagnosed with Covid-19 in Kuwait. *EClinicalMedicine*. 2020;24:100448. Epub 2020/08/09. doi: https://doi.org/10.1016/j.eclinm.2020.100448. PubMed PMID: 32766546; PubMed Central PMCID: 7335246.

78. Kasper MR, Geibe JR, Sears CL, Riegodedios AJ, Luse T, Von Thun AM, et al. An Outbreak of Covid-19 on an Aircraft Carrier. *N Engl J Med*. 2020;383(25):2417-26. Epub 2020/11/12. doi: https://doi.org/10.1056/nejmoa2019375. PubMed PMID: 33176077; PubMed Central PMCID: 7675688.

79. White EM, Santostefano CM, Feifer RA, Kosar CM, Blackman C, Gravenstein S, et al. Asymptomatic and Presymptomatic Severe Acute Respiratory Syndrome Coronavirus 2 Infection Rates in a Multistate Sample of Skilled Nursing Facilities. *JAMA Intern Med*. 2020;180(12):1709-11. Epub 2020/10/20. doi: https://doi.org/10.1001/jamainternmed.2020.5664. PubMed PMID: 33074318; PubMed Central PMCID: 7573793.

80. Jeffery-Smith A, Dun-Campbell K, Janarthanan R, Fok J, Crawley-Boevey E, Vusirikala A, et al. Infection and Transmission of Sars-Cov-2 in London Care Homes Reporting No Cases or Outbreaks of Covid-19: Prospective Observational Cohort Study, England 2020. *Lancet Reg Health Eur*. 2021;3:100038. Epub 20210122. doi: https://doi.org/10.1016/j.lanepe.2021.100038. PubMed PMID: 33870248; PubMed Central PMCID: PMC7826003.

81. Cardillo L, de Martinis C, Viscardi M, Esposito C, Sannino E, Lucibelli G, et al. Sars-Cov-2 Quantitative Real Time Pcr and Viral Loads Analysis among Asymptomatic and Symptomatic Patients: An Observational Study on an Outbreak in Two Nursing Facilities in Campania Region (Southern Italy). *Infect Agent Cancer*. 2021;16(1):45. Epub 20210622. doi: https://doi.org/10.1186/s13027-021-00388-x. PubMed PMID: 34158108; PubMed Central PMCID: PMC8218569.

82. Fisher MJ, Psevdos G. Asymptomatic Sars-Cov-2 Infection Following First Dose Mrna-1273 Covid-19 Vaccine in a Veterans Affairs Long Term Care Facility. *Am J Infect Control*. 2021;49(9):1210-1. Epub 20210625. doi: https://doi.org/10.1016/j.ajic.2021.06.010. PubMed PMID: 34175364; PubMed Central PMCID: PMC8225993.

83. Vohra LM, Jabeen D, Asif N, Ahad A. Covid-19 Pandemic and Breast Cancer Management: A Retrospective Observational Clinical Study from Pakistan. *Ann Med Surg (Lond)*. 2021;63:102151. Epub 20210204. doi: https://doi.org/10.1016/j.amsu.2021.01.099. PubMed PMID: 33564461; PubMed Central PMCID: PMC7860941.

84. Pamplona J, Solano R, Ramírez M, Durandez R, Mohamed F, Pardo L, et al. High Prevalence of Sars-Cov-2 Infection in Patients Scheduled for Digestive Endoscopy after the Peak of the First Wave of the Pandemic. *Gastroenterol Hepatol*. 2021;44(9):614-9. Epub 20210420. doi: https://doi.org/10.1016/j.gastrohep.2021.03.001. PubMed PMID: 33862154; PubMed Central PMCID: PMC8056966.

85. Rauch JN, Valois E, Ponce-Rojas JC, Aralis Z, Lach RS, Zappa F, et al. Comparison of Severe Acute Respiratory Syndrome Coronavirus 2 Screening Using Reverse Transcriptase-Quantitative Polymerase Chain Reaction or Crispr-Based Assays in Asymptomatic College Students. *JAMA Netw Open*. 2021;4(2):e2037129. Epub 2021/02/12. doi: https://doi.org/10.1001/jamanetworkopen.2020.37129. PubMed PMID: 33570576; PubMed Central PMCID: 7879237.

86. Garibaldi PMM, Ferreira NN, Moraes GR, Moura JC, Espósito DLA, Volpe GJ, et al. Efficacy of Covid-19 Outbreak Management in a Skilled Nursing Facility Based on Serial Testing for Early Detection and Control. *Braz J Infect Dis*. 2021;25(2):101570. Epub 20210323. doi: https://doi.org/10.1016/j.bjid.2021.101570. PubMed PMID: 33773990; PubMed Central PMCID: PMC7985963.

87. Lalani T, Lee TK, Laing ED, Ritter A, Cooper E, Lee M, et al. Sars-Cov-2 Infections and Serologic Responses among Military Personnel Deployed on the Usns Comfort to New York City During the Covid-19 Pandemic. *Open Forum Infect Dis*. 2021;8(2):ofaa654. Epub 20210123. doi: https://doi.org/10.1093/ofid/ofaa654. PubMed PMID: 33553482; PubMed Central PMCID: PMC7856331.

88. van Buul LW, van den Besselaar JH, Koene F, Buurman BM, Hertogh C, Group** C-N-S, et al. Asymptomatic Cases and Limited Transmission of Sars-Cov-2 in Residents and Healthcare Workers in Three Dutch Nursing Homes. *Gerontol Geriatr Med*. 2020;6:2333721420982800. Epub 2021/01/12. doi: https://doi.org/10.1177/2333721420982800. PubMed PMID: 33426178; PubMed Central PMCID: 7756037.

89. Ferreira VH, Chruscinski A, Kulasingam V, Pugh TJ, Dus T, Wouters B, et al. Prospective Observational Study and Serosurvey of Sars-Cov-2 Infection in Asymptomatic Healthcare Workers at a Canadian Tertiary Care Center. *PLoS One*. 2021;16(2):e0247258. Epub 2021/02/17. doi: https://doi.org/10.1371/journal.pone.0247258. PubMed PMID: 33592074; PubMed Central PMCID: 7886177.

90. Theuring S, Thielecke M, van Loon W, Hommes F, Hulso C, von der Haar A, et al. Sars-Cov-2 Infection and Transmission in School Settings During the Second Covid-19 Wave: A Cross-Sectional Study, Berlin, Germany, November 2020. *Euro Surveill*. 2021;26(34). Epub 2021/08/28. doi: https://doi.org/10.2807/1560-7917.es.2021.26.34.2100184. PubMed PMID: 34448448; PubMed Central PMCID: 8393892.

91. Varnell C, Jr., Harshman LA, Smith L, Liu C, Chen S, Al-Akash S, et al. Covid-19 in Pediatric Kidney Transplantation: The Improving Renal Outcomes Collaborative. *Am J Transplant*. 2021;21(8):2740-8. Epub 2021/01/17. doi: https://doi.org/10.1111/ajt.16501. PubMed PMID: 33452854; PubMed Central PMCID: 8013281.

92. Haidar G, Ayres A, King WC, McDonald M, Wells A, Mitchell SL, et al. Preprocedural Sars-Cov-2 Testing to Sustain Medically Needed Health Care Delivery During the Covid-19 Pandemic: A Prospective Observational Study. *Open Forum Infectious Diseases*. 2021;8(2). doi: https://doi.org/10.1093/ofid/ofab022.

93. Morgan SC, Aigner S, Anderson C, Belda-Ferre P, De Hoff P, Marotz CA, et al. Automated, Miniaturized, and Scalable Screening of Healthcare Workers, First Responders, and Students for Sars-Cov-2 in San Diego County. *medRxiv [Preprint]*. 2021:2021.06.25.21257885. doi: https://doi.org/10.1101/2021.06.25.21257885.

94. Orsi A, Domnich A, Pace VD, Ricucci V, Caligiuri P, Bottiglieri L, et al. Outbreak of Sars-Cov-2 Lineage 20i/501y.V1 in a Nursing Home Underlines the Crucial Role of Vaccination in Both Residents and Staff. *Vaccines*. 2021;9(6):591. PubMed PMID: https://doi.org/10.3390/vaccines9060591.

95. Hwang CE, Kussman A, Christle JW, Froelicher V, Wheeler MT, Moneghetti KJ. Findings from Cardiovascular Evaluation of National Collegiate Athletic Association Division I Collegiate Student-Athletes after Asymptomatic or Mildly Symptomatic Sars-Cov-2 Infection. *Clin J Sport Med*. 2022;32(2):103-7. doi: https://doi.org/10.1097/jsm.0000000000000954. PubMed PMID: 34173780.

96. Isoldi S, Mallardo S, Marcellino A, Bloise S, Dilillo A, Iorfida D, et al. The Comprehensive Clinic, Laboratory, and Instrumental Evaluation of Children with Covid-19: A 6-Months Prospective Study. *J Med Virol*. 2021;93(5):3122-32. Epub 20210216. doi: https://doi.org/10.1002/jmv.26871. PubMed PMID: 33570199; PubMed Central PMCID: PMC8014060.

97. Maki G, Abdollah F, Dabaja A, Suleyman G. 462. Prevalence and Outcome of Asymptomatic Procedural Patients with Covid-19 Infection. *Open Forum Infectious Diseases*. 2020;7(Supplement_1):S298-S. doi: https://doi.org/10.1093/ofid/ofaa439.655.

98. Fakhim H, Nasri E, Aboutalebian S, Gholipour S, Nikaeen M, Vaezi A, et al. Asymptomatic Carriers of Coronavirus Disease 2019 among Healthcare Workers in Isfahan, Iran. *Future Virology*. 2021;16(2):93-8. doi: https://dx.doi.org/10.2217%2Ffvl-2020-0224.

99. Green R, Tulloch JSP, Tunnah C, Coffey E, Lawrenson K, Fox A, et al. Covid-19 Testing in Outbreak-Free Care Homes: What Are the Public Health Benefits? *J Hosp Infect*. 2021;111:89-95. Epub 2021/01/17. doi: https://doi.org/10.1016/j.jhin.2020.12.024. PubMed PMID: 33453349; PubMed Central PMCID: 7837210.

100. Machado CM, Kerbauy MN, Colturato I, Arcuri LJ, dos Santos ACF, Silva FR, et al. Clinical Characteristics and Outcomes of Covid-19 in Hsct Recipients. *Blood*. 2020;136:19-. Epub 2021/08/03. doi: https://doi.org/10.1182/blood-2020-141959. PubMed PMID: PMC8330389.

101. Tian S, Wu M, Chang Z, Wang Y, Zhou G, Zhang W, et al. Epidemiological Investigation and Intergenerational Clinical Characteristics of 24 Coronavirus Disease Patients Associated with a Supermarket Cluster: A Retrospective Study. *BMC Public Health*. 2021;21(1):647. Epub 2021/04/03. doi: https://doi.org/10.1186/s12889-021-10713-z. PubMed PMID: 33794836; PubMed Central PMCID: 8016429.

102. Khondaker T, Qader MA, Gosh K, Chowdhury GN, Ferdous T, Afroz S, et al. Clinical Profile and Outcome of Covid -19 in Children with Pre-Existing Renal Disease. *Journal of Pediatric Nephrology*. 2020;9(1):1-6. doi: https://doi.org/10.22037/jpn.v9i1.33008.

103. Laws RL, Biraro S, Kirungi W, Gianetti B, Aibo D, Awor AC, et al. Coronavirus Disease 2019 (Covid-19) Mitigation Efforts and Testing During an in-Person Training Event-Uganda, 12-29 October 2020. *Clin Infect Dis*. 2021;73(Suppl 1):S42-s4. doi: https://doi.org/10.1093/cid/ciab331. PubMed PMID: 33912911; PubMed Central PMCID: PMC8135603.

104. Rincón A, Moreso F, López-Herradón A, Fernández-Robres MA, Cidraque I, Nin J, et al. The Keys to Control a Covid-19 Outbreak in a Haemodialysis Unit. *Clinical Kidney Journal*. 2020;13(4):542-9. doi: https://doi.org/10.1093/ckj/sfaa119.

105. Pizarro-Sanchez MS, Avello A, Mas-Fontao S, Stock da Cunha T, Goma-Garces E, Pereira M, et al. Clinical Features of Asymptomatic Sars-Cov-2 Infection in Hemodialysis Patients. *Kidney Blood Press Res*. 2021;46(1):126-34. Epub 2021/01/28. doi: https://doi.org/10.1159/000512535. PubMed PMID: 33503627; PubMed Central PMCID: 7900451.

106. Hogan CA, Gombar S, Wang H, Roltgen K, Shi RZ, Holubar M, et al. Large-Scale Testing of Asymptomatic Healthcare Personnel for Severe Acute Respiratory Syndrome Coronavirus 2. *Emerg Infect Dis*. 2021;27(1). Epub 2020/12/02. doi: https://doi.org/10.3201/eid2701.203892. PubMed PMID: 33256889; PubMed Central PMCID: 7774535.

107. Tan-Loh J, Cheong BMK. A Descriptive Analysis of Clinical Characteristics of Covid-19 among Healthcare Workers in a District Specialist Hospital. *Med J Malaysia*. 2021;76(1):24-8. Epub 2021/01/30. doi: https://pubmed.ncbi.nlm.nih.gov/33510104/. PubMed PMID: 33510104.

108. Xie W, Chen Z, Wang Q, Song M, Cao Y, Wang L, et al. Infection and Disease Spectrum in Individuals with Household Exposure to Sars-Cov-2: A Family Cluster Cohort Study. *J Med Virol*. 2021;93(5):3033-46. Epub 20210212. doi: https://doi.org/10.1002/jmv.26847. PubMed PMID: 33538342; PubMed Central PMCID: PMC8014049.

109. van den Besselaar JH, Sikkema RS, Koene F, van Buul LW, Oude Munnink BB, Frenay I, et al. Are Presymptomatic Sars-Cov-2 Infections in Nursing Home Residents Unrecognised Symptomatic Infections? Sequence and Metadata from Weekly Testing in an Extensive Nursing Home Outbreak. *Age Ageing*. 2021;50(5):1454-63. Epub 2021/05/09. doi: https://doi.org/10.1093/ageing/afab081. PubMed PMID: 33963830; PubMed Central PMCID: 8136016.

110. Schmitt J, Genet E, Danguy Des Deserts M, Chauvet-Atin S, Cungi PJ, Aries P, et al. Sars Cov2 Outbreak Management on a Landing Helicopter Dock: An Observational Retrospective Study. *Infect Dis Now*. 2021;51(5):424-8. Epub 20210619. doi: https://doi.org/10.1016/j.idnow.2021.06.303. PubMed PMID: 34157429; PubMed Central PMCID: PMC8214318.

111. Gettings JR, Gold JAW, Kimball A, Forsberg K, Scott C, Uehara A, et al. Severe Acute Respiratory Syndrome Coronavirus 2 Transmission in a Georgia School District-United States, December 2020-January 2021. *Clin Infect Dis*. 2022;74(2):319-26. doi: https://doi.org/10.1093/cid/ciab332. PubMed PMID: 33864375; PubMed Central PMCID: PMC8083290.

112. Patel MR, Kushwaha RS, Behera M, Bhadauria DS, Yachha M, Kaul A, et al. Aftermath of Fortnightly Universal Testing for Severe Acute Respiratory Corona Virus-2 Infection in Maintenance Hemodialysis Patients. *Semin Dial*. 2021;34(5):338-46. Epub 20210624. doi: https://doi.org/10.1111/sdi.12997. PubMed PMID: 34169574; PubMed Central PMCID: PMC8447208.

113. Meyers KJ, Dillman B, Williams C, Jiang J, Clifford N, Miller JL, et al. Follow-up of Sars-Cov-2 Positive Subgroup from the Asymptomatic Novel Coronavirus Infection Study. *J Med Virol*. 2021;93(5):2925-31. Epub 2021/01/20. doi: https://doi.org/10.1002/jmv.26810. PubMed PMID: 33463731; PubMed Central PMCID: 8014630.

114. Jones A, Fialkowski V, Prinzing L, Trites J, Kelso P, Levine M. Assessment of Day-7 Postexposure Testing of Asymptomatic Contacts of Covid-19 Patients to Evaluate Early Release from Quarantine - Vermont, May-November 2020. *MMWR Morb Mortal Wkly Rep*. 2021;70(1):12-3. Epub 2021/01/08. doi: http://dx.doi.org/10.15585/mmwr.mm7001a3. PubMed PMID: 33411700; PubMed Central PMCID: 7790157.

115. Weinbergerova B, Mayer J, Hrabovsky S, Novakova Z, Pospisil Z, Martykanova L, et al. Covid-19's Natural Course among Ambulatory Monitored Outpatients. *Sci Rep*. 2021;11(1):10124. Epub 20210512. doi: https://doi.org/10.1038/s41598-021-89545-1. PubMed PMID: 33980931; PubMed Central PMCID: PMC8115337.

116. Paleker M, Tembo YA, Davies MA, Mahomed H, Pienaar D, Madhi SA, et al. Asymptomatic Covid-19 in South Africa - Implications for the Control of Transmission. *Public Health Action*. 2021;11(2):58-60. doi: https://doi.org/10.5588/pha.20.0069. PubMed PMID: 34159063; PubMed Central PMCID: PMC8202632.

117. Esteban I, Bergero G, Alves C, Bronstein M, Ziegler V, Wood C, et al. Asymptomatic Covid-19 in the Elderly: Dementia and Viral Clearance as Risk Factors for Disease Progression. *TP92 TP092 CLINICAL ADVANCES IN SARS-COV-2 AND COVID-19*. 2021:A3826-A. doi: https://doi.org/10.1164/ajrccm-conference.2021.203.1_MeetingAbstracts.A3826.

118. Nunes MC, Baillie VL, Kwatra G, Bhikha S, Verwey C, Menezes C, et al. Severe Acute Respiratory Syndrome Coronavirus 2 Infection among Healthcare Workers in South Africa: A Longitudinal Cohort Study. *Clin Infect Dis*. 2021;73(10):1896-900. doi: https://doi.org/10.1093/cid/ciab398. PubMed PMID: 33949670; PubMed Central PMCID: PMC8135922.

119. Turunen T, Kontunen K, Sugulle K, Hieta P, Snellman O, Hussein I, et al. Covid-19 Outbreak at a Reception Centre for Asylum Seekers in Espoo, Finland. *J Migr Health*. 2021;3:100043. Epub 20210416. doi: https://doi.org/10.1016/j.jmh.2021.100043. PubMed PMID: 33880457; PubMed Central PMCID: PMC8051004.

120. Blain H, Gamon L, Tuaillon E, Pisoni A, Giacosa N, Albrand M, et al. Atypical Symptoms, Sars-Cov-2 Test Results and Immunisation Rates in 456 Residents from Eight Nursing Homes Facing a Covid-19 Outbreak. *Age Ageing*. 2021;50(3):641-8. doi: https://doi.org/10.1093/ageing/afab050. PubMed PMID: 33620381; PubMed Central PMCID: PMC7929417.

121. Say D, Crawford N, McNab S, Wurzel D, Steer A, Tosif S. Post-Acute Covid-19 Outcomes in Children with Mild and Asymptomatic Disease. *Lancet Child Adolesc Health*. 2021;5(6):e22-e3. Epub 20210421. doi: https://doi.org/10.1016/s2352-4642(21)00124-3. PubMed PMID: 33891880; PubMed Central PMCID: PMC8057863.

122. Beiting KJ, Huisingh-Scheetz M, Walker J, Graupner J, Martinchek M, Thompson K, et al. Management and Outcomes of a Covid-19 Outbreak in a Nursing Home with Predominantly Black Residents. *J Am Geriatr Soc*. 2021;69(5):1155-65. Epub 20210324. doi: https://doi.org/10.1111/jgs.17126. PubMed PMID: 33739444; PubMed Central PMCID: PMC8218575.

123. Uçkay I, Steinwender L, Burkhard J, Holy D, Strähl M, Farshad M. Outcomes of Asymptomatic Hospital Employees in Covid-19 Post-Exposure Quarantine During the Second Pandemic Wave in Zurich. *J Hosp Infect*. 2021;113:189-91. Epub 20210421. doi: https://doi.org/10.1016/j.jhin.2021.04.010. PubMed PMID: 33891983; PubMed Central PMCID: PMC8057933.

124. Hussain A, Satti L, Hanif F, Shoaib M, Ghauri MA, Khan Niazi GA, et al. Clinical and Virological Course of Sars-Cov 2 Infected Patients in a Tertiary Care Hospital in Pakistan. *J Ayub Med Coll Abbottabad*. 2020;32(Suppl 1)(4):S602-s6. doi: https://pubmed.ncbi.nlm.nih.gov/33754515/. PubMed PMID: 33754515; PubMed Central PMCID: 33754515.

125. Marcus JE, Frankel DN, Pawlak MT, Casey TM, Cybulski RJ, Jr., Enriquez E, et al. Risk Factors Associated with Covid-19 Transmission among Us Air Force Trainees in a Congregant Setting. *JAMA Netw Open*. 2021;4(2):e210202. Epub 20210201. doi: https://doi.org/10.1001/jamanetworkopen.2021.0202. PubMed PMID: 33630090; PubMed Central PMCID: PMC7907953.

126. Uysal E, Kilinçer A, Cebeci H, Özer H, Demir NA, Öztürk M, et al. Chest Ct Findings in Rt-Pcr Positive Asymptomatic Covid-19 Patients. *Clinical Imaging*. 2021;77:37-42. doi: https://doi.org/10.1016/j.clinimag.2021.01.030.

127. Hall VJ, Foulkes S, Charlett A, Atti A, Monk EJM, Simmons R, et al. Sars-Cov-2 Infection Rates of Antibody-Positive Compared with Antibody-Negative Health-Care Workers in England: A Large, Multicentre, Prospective Cohort Study (Siren). *Lancet*. 2021;397(10283):1459-69. Epub 20210409. doi: https://doi.org/10.1016/s0140-6736(21)00675-9. PubMed PMID: 33844963; PubMed Central PMCID: PMC8040523.

128. Malhotra S, Rahi M, Das P, Chaturvedi R, Chhibber-Goel J, Anvikar A, et al. Epidemiological Profiles and Associated Risk Factors of Sars-Cov-2 Positive Patients Based on a High-Throughput Testing Facility in India. *Open Biol*. 2021;11(6):200288. Epub 20210602. doi: https://doi.org/10.1098/rsob.200288. PubMed PMID: 34062097; PubMed Central PMCID: PMC8169211.

129. Abraha HE, Gessesse Z, Gebrecherkos T, Kebede Y, Weldegiargis AW, Tequare MH, et al. Clinical Features and Risk Factors Associated with Morbidity and Mortality among Patients with Covid-19 in Northern Ethiopia. *Int J Infect Dis*. 2021;105:776-83. Epub 20210316. doi: https://doi.org/10.1016/j.ijid.2021.03.037. PubMed PMID: 33741488; PubMed Central PMCID: PMC7962557.

130. Ren R, Zhang Y, Li Q, McGoogan JM, Feng Z, Gao GF, et al. Asymptomatic Sars-Cov-2 Infections among Persons Entering China from April 16 to October 12, 2020. *Jama*. 2021;325(5):489-92. doi: https://doi.org/10.1001/jama.2020.23942. PubMed PMID: 33528529; PubMed Central PMCID: PMC7856538.
