# Supplementary material for: Occurrence and transmission potential of asymptomatic and presymptomatic SARS-CoV-2 infections: Update of a living systematic review and meta-analysis
Source: PLoS Med. 2022 May 26;19(5):e1003987. doi: 10.1371/journal.pmed.1003987 (PMC9135333; doi:10.1371/journal.pmed.1003987)
Supplement: S5 Fig — NA, not applicable; NEI: not enough information; NR: not reported; PY: partially yes. (PDF) [file pmed.1003987.s015.pdf]

S5 Figure. Relevance and credibility of mathematical modelling studies assessing contribution of asymptomatic and pre-symptomatic infection to SARS-CoV-2 transmission

AUTHOR[a]	Is the population relevant?	Are any critical interventions missing?	Is the context (settings and circumstances) applicable?	Is external validation of the model sufficient to make its results credible for your decision?	Is internal verification of the model sufficient to make its results credible for your decision?	Does the model have sufficient face validity to make its results credible for your decision?	Is the design of the model adequate for your decision problem?	Are the data used in populating the model suitable for your decision problem?	Were the analyses performed using the model adequate to inform your decision problem?	Was there an adequate assessment of the effects of uncertainty?	Was the reporting of the model adequate to inform your decision problem?	Was the interpretation of results fair and balanced?	Were there any potential conflicts of interest?
Ferretti L [1]	PY	NA	YES	NO	YES	YES	YES	PY	YES	YES	YES	NO	NO
Zhang W [2]	PY	NA	YES	NO	YES	NO	PY	PY	NO	NO	NO	NO	NR
He X [3]	PY	NA	YES	NO	YES	YES	YES	PY	YES	YES	PY	YES	NO
Peak CM [4]	YES	NA	PY	NO	YES	PY	YES	NO	PY	PY	PY	YES	NO
Emery JC [5]	YES	NA	PY	YES	YES	YES	YES	PY	YES	YES	YES	YES	NO
Tindale LC [6]	YES	NA	YES	YES	YES	YES	PY	YES	YES	YES	YES	YES	NO
Moghadas SM [7]	YES	NA	YES	NO	NO	NO	YES	YES	YES	PY	NO	NO	NO
Ren X [8]	YES	NA	YES	NO	NO	NO	NO	YES	YES	NO	YES	PY	NO
Chun JY [9]	YES	NA	YES	YES	YES	YES	YES	YES	YES	YES	YES	YES	NO
Sun K [10]	YES	NA	YES	PY	PY	NEI	PY	YES	YES	PY	YES	YES	NO
Bushman M [11]	YES	NA	YES	NO	YES	NO	YES	YES	YES	YES	YES	YES	NO
Wu P [12]	YES	NA	YES	YES	YES	YES	YES	YES	YES	YES	YES	YES	NO
Tan J [13]	YES	NA	YES	NO	NO	YES	YES	YES	YES	YES	YES	YES	NO

a. Reference numbers as per the main text

PY: Partially yes

NA: Not applicable

NR: Not reported

NEI: Not enough Information

References

1. Ferretti L, Wymant C, Kendall M, Zhao L, Nurtay A, Abeler-Dorner L, et al. Quantifying Sars-Cov-2 Transmission Suggests Epidemic Control with Digital Contact Tracing. Science. 2020;368(6491). Epub 2020/04/03. doi: https://doi.org/10.1126/science.abb6936. PMID: 32234805; PubMed Central PMCID: PMC7164555.
2. Zhang W. Estimating the Presymptomatic Transmission of Covid19 Using Incubation Period and Serial Interval Data. bioRxiv [Preprint]. 2020. doi: https://doi.org/10.1101/2020.04.02.20051318. PMID.
3. He X, Lau EHY, Wu P, Deng X, Wang J, Hao X, et al. Temporal Dynamics in Viral Shedding and Transmissibility of Covid-19. Nat Med. 2020;26(5):672-5. Epub 2020/04/17. doi: https://doi.org/10.1038/s41591-020-0869-5. PMID: 32296168.
4. Peak CM, Kahn R, Grad YH, Childs LM, Li R, Lipsitch M, et al. Individual Quarantine Versus Active Monitoring of Contacts for the Mitigation of Covid-19: A Modelling Study. Lancet Infect Dis. 2020;20(9):1025-33. Epub 2020/05/24. doi: https://doi.org/10.1016/s1473-3099(20)30361-3. PMID: 32445710; PubMed Central PMCID: 7239635.
5. Emery JC, Russell TW, Liu Y, Hellewell J, Pearson CA, Group CC-W, et al. The Contribution of Asymptomatic Sars-Cov-2 Infections to Transmission on the Diamond Princess Cruise Ship. Elife. 2020;9. Epub 2020/08/25. doi: https://doi.org/10.7554/elife.58699. PMID: 32831176; PubMed Central PMCID: 7527238.
6. Tindale LC, Stockdale JE, Coombe M, Garlock ES, Lau WYV, Saraswat M, et al. Evidence for Transmission of Covid-19 Prior to Symptom Onset Elife. 2020;9. Epub 2020/06/23. https://doi.org/10.7554/elife.57149. doi. PMID: 32568070; PubMed Central PMCID: 7386904.
7. Moghadas SM, Fitzpatrick MC, Sah P, Pandey A, Shoukat A, Singer BH, et al. The Implications of Silent Transmission for the Control of Covid-19 Outbreaks. Proc Natl Acad Sci U S A. 2020;117(30):17513-5. Epub 2020/07/08. doi: https://doi.org/10.1073/pnas.2008373117. PMID: 32632012; PubMed Central PMCID: 7395516.
8. Ren X, Li Y, Yang X, Li Z, Cui J, Zhu A, et al. Evidence for Pre-Symptomatic Transmission of Coronavirus Disease 2019 (Covid-19) in China. Influenza Other Respir Viruses. 2021;15(1):19-26. Epub 2020/08/09. doi: https://doi.org/10.1111/irv.12787. PMID: 32767657; PubMed Central PMCID: 7436222.
9. Chun JY, Baek G, Kim Y. Transmission Onset Distribution of Covid-19. Int J Infect Dis. 2020;99:403-7. Epub 2020/08/11. doi: https://doi.org/10.1016/j.ijid.2020.07.075. PMID: 32771633; PubMed Central PMCID: 7409940.
10. Sun K, Wang W, Gao L, Wang Y, Luo K, Ren L, et al. Transmission Heterogeneities, Kinetics, and Controllability of Sars-Cov-2. Science. 2021;371(6526). Epub 2020/11/26. doi: https://doi.org/10.1126/science.abe2424. PMID: 33234698; PubMed Central PMCID: 7857413.
11. Bushman M, Worby C, Chang HH, Kraemer MUG, Hanage WP. Transmission of Sars-Cov-2 before and after Symptom Onset: Impact of Nonpharmaceutical Interventions in China. Eur J Epidemiol. 2021;36(4):429-39. Epub 2021/04/22. doi: https://doi.org/10.1007/s10654-021-00746-4. PMID: 33881667; PubMed Central PMCID: 8058147.
12. Wu P, Liu F, Chang Z, Lin Y, Ren M, Zheng C, et al. Assessing Asymptomatic, Presymptomatic, and Symptomatic Transmission Risk of Severe Acute Respiratory Syndrome Coronavirus 2. Clin Infect Dis. 2021;73(6):e1314-e20. doi: https://doi.org/10.1093/cid/ciab271. PMID: 33772573; PubMed Central PMCID: PMC8083716.
13. Tan J, Ge Y, Martinez L, Sun J, Li C, Westbrook A, et al. Transmission Roles of Symptomatic and Asymptomatic Covid-19 Cases: A Modeling Study. medRxiv [Preprint]. 2021:2021.05.11.21257060. doi: https://doi.org/10.1101/2021.05.11.21257060. PMID.
