# Supplementary material for: Occurrence and transmission potential of asymptomatic and presymptomatic SARS-CoV-2 infections: Update of a living systematic review and meta-analysis
Source: PLoS Med. 2022 May 26;19(5):e1003987. doi: 10.1371/journal.pmed.1003987 (PMC9135333; doi:10.1371/journal.pmed.1003987)
Supplement: Attachment — Submitted filename: review_letter0215.docx [file pmed.1003987.s016.docx]

Dear editor:

This paper is clearly written and well organized. I suggest a minor revision.

The highlights of this article are very obvious. First, this is a time-dynamic article. The methods authors used were posted online and updated 3 times. The study spans a large period. And the methods part and result part was detailed and informative. Second, a new method was invented and hired in this article to assess the bias of each included article. Third, this article emphasized prediction interval instead of single summary estimates of the percentage of asymptomatic infection due to the high Heterogeneity, which other meta-analyses may not emphasize. Fourth, this article was well organized. And two question this article try to figure out was innovative, including the attacking rate of asymptomatic infection and what proportion of SARS-CoV-2 transmission is accounted for by asymptomatic people.

**Changes need to be made:**

**Information update:**

I do understand the workload of this living mate analysis is tremendous. But this review includes 94 articles updated up to 2 February 2021 and now is 2022. I think the authors shall update some information, which can be put in the discussion part of this article. This causes some change in this part but does not affect the conclusion:

1. Information update about variants is necessary. One year has been gone since the latest data update. SARS-Cov-2 already went through some epidemical changes. As it is said in line 454. The variance of SARS-Cov-2 starts to appear in 2021. And studies show that variants already occupy major popularity of new infections in 2022. According to some studies, variants of SARS-Cov-2 already manifest different clinical features. The result may be challenged due to the epidemic of two main variant viruses. This is a limit of this article and more information about variants asymptomatic infection rate need to be discussed.

Page 22 Line 457. Variants are an important part of the epidemic, which can change the asymptomatic infection rate. Not only the omicron variance, which appeared in November 2021, but other variance also come to appear in 2021, First, the delta variant, named in May 2021, shows more powerful infection ability and spreads quickly all over the world. Then the omicron variant showing up in November 2021. Those variants brought huge changes to the global virus Epidemic. There are already studies showing that the omicron variant has a more asymptomatic infection rate. Which may affect the percentage of asymptomatic infection rate. That information needs to be added.

2. In the result part, it shows that the estimated percentage does not change during the 3 times articles update (Page 15 line 311-312). So, the authors conclude that’ preliminary analyses show that the main findings do not change when including studies published up to April 2021’ (Page 19 Line 382-383). This conclusion is limited. It only can be said that it represents the data of Infection of non-mutant strains which is before 2021.

3. Page 19 Line 388 also needed to be updated. The study already shows that vaccinated people could be infected by a new variant of SARS-Cov-2 in Omicron infection. As an article will be published in 2022, the conclusion from articles before 2021 is limited.

**Some small suggestions:**

1. Page 3 lines 78-79: how many studies were based on screening of defined populations and how many studies were at low risk of information bias may be added.

2. Page 6 line 134: Question (3). Since studies concerning Q3 were all calculated by modeling. This should be clarified here. This result is inferred from modeling calculations, not from direct observation.

3. Page 14 Line 286: How many studies including contact investigations and outbreak investigation shall be added.

4. Page 14 lines 288-294: IQR of subgroup analysis may need to be added.

3. Page 15 line 308: the statement ‘additional analyses’ was too general. This part may be Combined with the previous paragraph. Or change with a more specific title.

**Some small grammar mistakes:**

1. Page7 line 151: ‘Ovid Embase, bioRxiv and medRxiv),’ a comma may be inserted before ‘and’

2. Page 8 line 184: “The extracted variables included, the study” coma may be removed.
