# Supplementary material for: Occurrence and transmission potential of asymptomatic and presymptomatic SARS-CoV-2 infections: Update of a living systematic review and meta-analysis
Source: PLoS Med. 2022 May 26;19(5):e1003987. doi: 10.1371/journal.pmed.1003987 (PMC9135333; doi:10.1371/journal.pmed.1003987)
Supplement: Attachment — Submitted filename: Replytocomm_asymptomaticreview_upload.docx [file pmed.1003987.s017.docx]

| **Response to reviewers’ comments**  Thank you very much for these helpful and insightful comments. Our revised manuscript is a new update of the living systematic review, which extends the period of the search to include publications to 6 July 2021. The update includes three publications with data collected since vaccines have been rolled out and variants of concern have emerged. The revised analyses did not change our main findings and conclusions.  We include a point-by-point response to the editors’ and reviewers’ comments. We include quotations of text that we have changed, referring to page and paragraph numbers, or line numbers of the text in the clean version of the manuscript.  In this revised version, we have made some other changes to improve readability. We reduced the word count (from 5200 to around 4700) and reduced the number of supplementary figures. | |
| --- | --- |
| **COMMENT** | **Authors’ response** |
| **Editors** |  |
| 1. As requested by one or more of our reviewers, please update the search to the most recent date possible. | We have updated the review until 6th July with 40 additional studies. The updated results did not change our main conclusions, but we were able to include three studies in the period after vaccines were introduced and variants of concern emerged. |
| 2. After the abstract, please add a new and accessible "author summary" section in non-identical prose. You may find it helpful to consult one or two recent research papers published in PLOS Medicine to get a sense of the preferred style. | We have written an author summary (page 5) |
| 3. At line 61, we suggest "we conducted an update ..." or similar. | Abstract, p3, line 62. We say, “We updated a living systematic review…” and we repeat this in the first sentence in the Methods section in the main text. |
| 4. Please remove the information on funding and competing interests from the end of the main text. In the event of publication, this will appear in the article metadata, via entries in the submission form. | We have deleted information on funding and competing interests, but left acknowledgements and author contributions |
| 5. Noting one entry in table 2, please quote exact p values or "p<0.001" throughout, unless there are specific reasons to do otherwise. | Table 2 has been replaced and updated with additional findings from subgroup and meta-regression analyses, with exact p values. |
| 6. Throughout the text, please remove spaces from within the square brackets for reference call-outs (e.g., "... more [2,3]."). | We have edited the output style, as requested to deal with requests 6 to 8 |
| 7. In the reference list, please add "[preprint]" to all preprint citations, noting reference 33 and others. |  |
| 8. Noting reference 10, please use the journal name abbreviation "PLoS Med."; and for reference 57 "PLoS ONE". |  |
| 9. Noting reference 95 and others, please ensure that all references have full access details. | We have revised all the references and have added full access details |
| 10. Thank you very much for including the PRISMA checklist. Please adapt this so that individual items are referred to by section (e.g., "Methods") and paragraph numbers, not by line or page numbers as these generally change in the event of publication. | We have adapted the PRISMA checklist each item has a section, page number and paragraph number. |
| 11. Please remove the attached earlier version of the systematic review. | The earlier published version has been removed |
| **Reviewer #1**  This is an excellent review of an important set of questions |  |
| 1. The review is stated as being a "living" review, but the literature was only searched up to February 2021.  So what is the definition of a living review, according to these investigators. A review of publications up to one year ago does not seem to fit with definitions of living reviews. | We understand the authors’ concern. We use the publication written by Elliott et al.’s definition of a living systematic review (p7, Methods, first sentence), “a systematic review that provides an online summary of findings and is updated when relevant new evidence becomes available.”  This fifth version is now the most up to date systematic review of the subject and we think that is still justifiable to call it a living systematic review. We have acknowledged the delay (p18, Discussion, Strengths and weaknesses) “Although we made extensive efforts to comply with the planned 3-monthly updates, with weekly searches and a continuous process of screening, data extraction and risk of bias assessment, the pace of publications about SARS-CoV-2 exceeds the capacity of our crowd of reviewers”. |
| 2. While it is understandable that the production of literature and resources to conduct the review process is limiting the ability to keep up to date, |  |
| 2a. …it would be good to think about restrictions that could be made to streamline this process and allow for more recent updating (eg dropping pre-prints, increasing minimal sample size for eligibility, restricting to particular study designs, excluding based on certain quality criteria). | 2a. We have clarified in the Methods which of the reviewer’s suggested restrictions we currently use (p9, Study selection), “Reviewers, including crowdsourced trained volunteers, worked in pairs to screen records using an application programming interface in the electronic data capture system (REDCap…” And exclusion of some study types (p7 and 8, Eligibility criteria and protocol (<https://osf.io/78qe4/>, v5.1).  We thank the reviewer for mentioning preprints. We chose to include these and now mention this in the Discussion (p18, Strengths and weaknesses) “Our decision to include preprints compensates for some of the delay because these articles appear sooner than peer-reviewed publications.” |
| 2b. The WHO Covid-19 database is a one-stop shop that could be used instead of searching 4 separate databases | 2b. We agree that data aggregators also increase efficiency and have clarified (p8, Methods, Information sources, lines 179-180) that we use “the COVID-19 living evidence database [26], which uses automated workflow processes to: (1) aggregate simultaneous daily searches of four electronic databases...” |
| 3. More updated information may also contribute information about the predominant variant in circulation at the time of the study. | Thank you. We agree and have included a statement in the Results (p11, end of first paragraph) “The review period from 1 January 2021 onwards includes 52 publications, three of which collected data during the period when the alpha variant of concern [85,125,133] had been described and vaccines were being introduced.”  Please also see our response to Reviewer #2, comment 1 |
| The Comparison with other reviews is helpful and could be summarised graphically. | Thank you for this suggestion. We have added a forest plot to these findings, which were reported in the former S4 Table; the new information is now in S2 Appendix. |
| **Reviewer #2** |  |
| 1. This is a thoughtful and well-written update to a living systematic review and meta-analysis examining the prevalence and roles of asymptomatic/presymptomatic infection on the spread of COVID-19. To rely perhaps a bit too much on Greek myths, what began as a Herculean task now seems a Sisyphean one, given the vast numbers of papers and preprints on COVID-19. This update only goes through Feb 1, 2021, and thus misses the potential impact of immunity from infection and/or vaccination on the extent of symptomatic disease and transmission and similarly how these factors change by the dominant variant. I appreciate the authors reasoned approach to noting the massive heterogeneity in the studies reviewed and their caution in applying meta-analysis tools not appropriate for the massive variability in findings across studies. | Thank you for the sympathetic response. With the revised manuscript, we have updated the search to 6 July 2021 and our main findings are unchanged.  We have provided more information about the impact of vaccines and variants of concern in our response below. Please also see our response to Reviewer #1, comment 3. |
| 2. A primary takeaway seems to be that these types of reviews may be overwhelmed by both the tsunami of reports and the rapidly changing landscape with widespread infection and vaccination and shifting variants, where each of these factors may contribute to differences in results for each of the three questions this study focuses on. As such, my main comment for the authors is to clarify how much they expect variants and immunity from both vaccination and infection to play a role and whether their study is focused just on 'wild-type' or is generally a question that can be answered across the multiple dimensions of immune history, dominant variant, and potentially other elements. The abstract and paper end by mentioning 'wild-type' SARS-CoV-2. Given the D614G mutation that was dominant even within the timeframe of this review/metaanalysis, was this really only a study of wild-type SARS-CoV-2 (as the last lines of the abstract and paper suggest)? | Thank you for these comments, which we partly address in our response to Reviewer #1, comment 3. Apologies for the misnomer “wild-type”, which we have deleted. We meant this to include all earlier variants that were not variants of concern.  We have revised the Discussion to deal more explicitly with this pertinent question, especially as we had expected that (p19, lines 448-449) “persistently asymptomatic SARS-CoV-2… should be influenced mainly by properties of the virus and the host response to infection.”  We have added to the Implications and unanswered questions (p21, lines 492-501), “Determining the viral dynamics and full clinical spectrum of infection with variants of concern is important. Variants classed as omicron differ substantially from all earlier SARS-CoV-2 variants, with high infectiousness and immune evasion [188], and viral characteristics and immunity could influence the occurrence of asymptomatic infection. Studies published in early 2022 are already reporting a wide range of estimates of asymptomatic omicron infection. In India, from the date of emergence of the omicron variant, 24 November 2021 to 4 January 2022, authors reported a high proportion of asymptomatic omicron variant infections (56.7% of 291) but did not report any follow-up and >80% of participants had been vaccinated [189] . In contrast, authors of a cohort study of an outbreak of omicron SARS-CoV-2 in Norway, found only 1 of 81 infections in a highly vaccinated group was asymptomatic after 10 days of follow-up [190].” |
|  |  |
|  |  |
|  |  |
|  |  |
|  |  |
| 2a. It seems that no prospective study could be done to look at these questions any longer for that strain, but what about for other strains? I'd be interested in the authors thoughts on what kinds of studies will be needed now, given widespread population immune experience from vaccination & infection, and repeated replacement of dominant variants. | Thank you. We have also added to our Discussion of future study designs (p21, Discussion, Implications, lines 501-509. “There are increasing challenges for studies relying on routine health service or surveillance data; in many jurisdictions, indications for routine testing are being reduced, which will make selection biases more likely, and mandated quarantine and isolation periods for people with diagnosed SARS-CoV-2 infection are being reduced, which will increase information biases in the ascertainment of persistent asymptomatic status. Researchers need to design studies to address this specific research question for each variant of concern, taking into account vaccination status and prior infection. There are ongoing prospective studies that collect appropriate data [125], for which improved reporting could address the requirements for assessing asymptomatic infection status fully, but ongoing funding for these studies is not secure [191]. |
| **Reviewer #3** |  |
| 1. This manuscript provides an update to the previous systematic review and meta-analysis of asymptomatic COVID-19 infections. Using 94 studies, the authors found that 13-45% of infections are asymptomatic, although the authors note high between-study heterogeneity. While the paper is well written, in my opinion it does not add a lot to the growing body of literature that has been published since the previous version. | We hope that our revised manuscript shows that added value of the updated systematic review. We believe that our study makes an important methodological contribution by stating that the use of meta-analysis to produce an overall summary estimate for this body of evidence is not justified. |
| 2. My major concern with this article is that the database was last updated a year back on February 2, 2021. To justify a publication as a living systematic review and meta-analysis, the database needs to be updated. Additionally, as the authors mention in the introduction, several recent meta-analyses on asymptomaticity have been published, providing more up to do date systematic reviews and meta-analyses of the literature. For example:  Ma et al. performed a similar analysis with their database updated on  Feb 4, 2021. <https://jamanetwork.com/journals/jamanetworkopen/fullarticle/2787098> Number of studies included in the meta-analysis of asymptomatic percentage: 95  Sah et al. Last database update: April 2021 <https://www.pnas.org/content/118/34/e2109229118#sec-3> Number of studies included in the meta-analysis of asymptomatic percentage: 170 | We have updated our search to 6 July 2021 and now include 130 studies about the proportion of asymptomatic SARS-CoV-2. The smaller number of studies included in our review, compared with Sah, et al. reflects more restrictive inclusion criteria. |
| 3. a. Following my previous point, Table 1 indicates that only 13 studies published between January 2021 - June 2021 were included in the analysis which is probably because the database wasn't updated after February 2021. | The reviewer is correct. With our updated search, we have included 52 studies in the period from 1 Jan 2021 to 6 Jul 2021. |
| 3. b. However, page 11 line 257-258 mentions "At the time of the latest search date,17 records were preprints, 14 of which had been  published in peer-reviewed journals by 23 November 2021. Does this mean that another search was performed on 23 November 2021? If there was another search performed in November 2021, why weren't any studies published after June 2021 included in the analysis? | We thank the reviewer for bringing this point to our attention. We meant that we checked the publication status but did not conduct a new search on 23 November 2021. We have revised the text to say (Results, lines 269-271), “At the time of the search on 6 July 2021, five records were preprints. We checked the publication status on 14 March and all remained as preprints [61,62,88,169,171].” |
| 4. Definition of asymptomatic infection: The analyses include studies where COVID diagnosis was made based on antibody test rather than RT-PCR test. For example, the study Pirnay JP, 2020 observed four individuals with no symptoms, but only two of these were positive RT-qPCR. The other two were negative RT-qPCR but tested positive on antibody tests. The study itself notes "two individuals (M8 and M9), only the rapid antibody tests were positive, making it uncertain whether they actually had the disease. This manuscript assumes that these two individuals were asymptomatic. However, since the dates of exposure for these two individuals are uncertain, assuming that the positive antibody (but PCR negative) test is an indication of active infection is incorrect. As such, any study that used an antibody test to diagnose an asymptomatic infection should be excluded from the analysis. | Thank you for pointing out this error. We have corrected this (Fig 1 and S2 Table). We should not have included diagnoses made by antibody test, in line with our protocol “Condition: SARS-CoV-2 infection diagnosed with a molecular test from any anatomical site.” |
| 5. Lack of symptom follow-up in studies included in the analysis: Some of the studies included in the analysis do not report an absence of symptoms at the end of follow-up, but despite this lack of data, these individuals are categorized as asymptomatic in this manuscript. For example, Bi Q, 2020 (reference 2, Table S2) mentions "At the time of the first clinical assessment …..and 17 (20%) of 87 had no symptoms." The study does not specify that these individuals remained symptom-free at the end of follow-up, and so cannot be categorized as asymptomatic. Such studies should be removed from the meta-analysis of asymptomaticity. | Thank you for pointing this out. We have removed the study by Bi, which was included before we tightened our definition of the outcome (protocol amendment 14.10.2020). This study is now listed in S1 Table, which reports on studies that were previously included, but were excluded from this version of the review. |
| 6. Bias arising due to over-representation of symptomatic cases: Since the studies included in the meta analysis were not designed to calculate asymptomatic percentage, there is an over representation of cases symptomatic cases in most studies, biasing the asymptomatic percentage towards a lower estimate. For example, Lee JY, 2020 investigated inpatients diagnosed with COVID-19 where 20% of the patients had severe disease. In such a setting, it is likely that the majority of patients were admitted because they were exhibiting symptoms and therefore asymptomatic individuals were likely under-represented. Additionally, surveillance studies would also suffer from this bias due to a higher willingness of symptomatic individuals to participate/provide consent. This issue has been highlighted and corrected by a recent meta-analysis (Sah et. al, 2021). | We agree with the reviewer and acknowledged this in our Discussion (p20, lines 477-478), “Part of the heterogeneity arises from the fact that many studies included in this review were not designed to estimate the proportion of asymptomatic SARS-CoV-2 infection.”  As the reviewer suggests, some individuals are more likely to be included in a study because of their symptoms. We incorporated the suggestion of Sah, et al. (Synthesis of the evidence, lines 237-240) “ in contact or outbreak investigations, we subtracted the index cases from the total with SARS-CoV-2 infection, because these people were likely to have been identified because of their symptoms and their inclusion might lead to underestimation of the asymptomatic proportion [16].” |
| 6b. Although performing a subgroup analysis by study design attempts to approach this issue, all study designs (except the ones where 100% of the population was sampled) will have this inherent selection bias. Without a correction, the estimates of asymptomaticity presented in this meta-analysis are unreliable. | Thank you. In a new meta-regression analysis, we found that study design explained 16% of the heterogeneity in results (Table 2).  We agree that overall estimates from meta-analysis are unreliable and chose to present interquartile ranges and prediction intervals instead. For studies based on screening of defined populations, we did not find a simple correction that could be applied.  We believe that the heterogeneity results from a range of biases, including both selection and information biases (Table 2). Based on our protocol, we did exclude some studies (S1 Table) that could have underestimated the asymptomatic proportion. We also describe the risks of bias in individual studies and compared estimates in groups of studies at low versus unclear or high risk of bias, stratified by study design (S4 table). |
| 7. Additional analysis: Performing a subgroup analysis of asymptomatic proportion by publication date is likely to be biased due to the publication efficiency of different journals (it may take anytime between 1 month to 6m+ to publish an article). A more informative analysis would look at the last date of data collection mentioned in the study. | We agree with this point. Although S4 Fig, showed only a difference between the first and second time periods, in the new meta-regression analysis, with publication date as a continuous variable, we did find evidence of an association with publication date (Table 2).  We agree that there are delays in publication. We did not record dates of data collection for all studies. But, for the most recent studies (p11, Results), “The review period from 1 January 2021 onwards includes 52 publications, three of which collected data during the period when the alpha variant of concern [85,125,133] had been described and vaccines were being introduced.” |
| **Reviewer #4** |  |
| This paper is clearly written and well organized. I suggest a minor revision.  The highlights of this article are very obvious. First, this is a time-dynamic article. The methods authors used were posted online and updated 3 times. The study spans a large period. And the methods part and result part was detailed and informative. Second, a new method was invented and hired in this article to assess the bias of each included article. Third, this article emphasized prediction interval instead of single summary estimates of the percentage of asymptomatic infection due to the high Heterogeneity, which other meta-analyses may not emphasize. Fourth, this article was well organized. And two question this article try to figure out was innovative, including the attacking rate of asymptomatic infection and what proportion of SARS-CoV-2 transmission is accounted for by asymptomatic people. |  |
| 1. Information update about variants is necessary. One year has been gone since the latest data update. SARS-Cov-2 already went through some epidemical changes. As it is said in line 454. The variance of SARS-Cov-2 starts to appear in 2021. And studies show that variants already occupy major popularity of new infections in 2022. According to some studies, variants of SARS-Cov-2 already manifest different clinical features. The result may be challenged due to the epidemic of two main variant viruses. This is a limit of this article and more information about variants asymptomatic infection rate need to be discussed. Page 22 Line 457. Variants are an important part of the epidemic, which can change the asymptomatic infection rate. Not only the omicron variance, which appeared in November 2021, but other variance also come to appear in 2021, First, the delta variant, named in May 2021, shows more powerful infection ability and spreads quickly all over the world. Then the omicron variant showing up in November 2021. Those variants brought huge changes to the global virus Epidemic. There are already studies showing that the omicron variant has a more asymptomatic infection rate. Which may affect the percentage of asymptomatic infection rate. That information needs to be added. | Thank you for this important comment. We hope that our responses to Reviewer #1, comment 3 and Reviewer #2, comment 2 address this concern.  In addition, we searched for new studies about asymptomatic infections with the omicron variant and summarise the challenges of such studies (p21, lines 495-505, Discussion, Implications, “Studies published in early 2022 are already reporting a wide range of estimates of asymptomatic omicron infection. In India, from the date of emergence of the omicron variant, 24 November 2021 to 4 January 2022, authors reported a high proportion of asymptomatic omicron variant infections (56.7% of 291) but did not report any follow-up and >80% of participants had been vaccinated [189] . In contrast, authors of a cohort study of an outbreak of omicron SARS-CoV-2 in Norway, found only 1 of 81 infections in a highly vaccinated group was asymptomatic after 10 days of follow-up [190]. There are increasing challenges for studies relying on routine health service or surveillance data; in many jurisdictions, indications for routine testing are being reduced, which will make selection biases more likely, and mandated quarantine and isolation periods for people with diagnosed SARS-CoV-2 infection are being reduced, which will increase information biases in the ascertainment of persistent asymptomatic status. .” |
| 2. In the result part, it shows that the estimated percentage does not change during the 3 times articles update (Page 15 line 311-312). So, the authors conclude that’ preliminary analyses show that the main findings do not change when including studies published up to April 2021’ (Page 19 Line 382-383). This conclusion is limited. It only can be said that it represents the data of Infection of non-mutant strains which is before 2021. | We agree. We have added to the Discussion (p17, Summary of findings, last sentence), “Evidence about asymptomatic infections caused by variants of concern, or with immunity following infection or vaccination, is limited.” |
| 3. Page 19 Line 388 also needed to be updated. The study already shows that vaccinated people could be infected by a new variant of SARS-Cov-2 in Omicron infection. As an article will be published in 2022, the conclusion from articles before 2021 is limited. | Thank you. In the revised version, we have deleted this sentence. |
| 1. Page 3 lines 78-79: how many studies were based on screening of defined populations and how many studies were at low risk of information bias may be added. | In the Abstract, we have added that there were 84 studies based on screening of defined populations. To shorten the Abstract we have not reported on subgroups according to risk of bias. |
| 3. Page 14 Line 286: How many studies including contact investigations and outbreak investigation shall be added. | In the Results (p12, line 286), we now report that there were 46 studies based on contact and outbreak investigations. |
| 4. Page 14 lines 288-294: IQR of subgroup analysis may need to be added. | IQRs of subgroups are now reported (p13), “The estimated proportions of asymptomatic SARS-CoV-2 infections were similar in studies of contact investigations (16%, 95% CI 9-27%, IQR 8-38%, 13 studies) and outbreak investigations (20%, 95% CI 15-28%, IQR 8-38%, 33 studies)… The ranges of estimates were similar in three settings in which screening studies were conducted; people in a community setting (23 studies, IQR 20-53%, prediction interval 2-96%), institutional settings such as nursing homes (43 studies, IQR 26-67%, prediction interval 5-93%), and occupational settings such as amongst groups of healthcare workers (18 studies, IQR 17-64%, prediction interval 3-95%)“ |
| 3. Page 15 line 308: the statement ‘additional analyses’ was too general. This part may be Combined with the previous paragraph. Or change with a more specific title. | ‘Additional analyses’ is a PRISMA item to show those not pre-specified in the review protocol. We have changed the subheading to “Factors associated with proportion of asymptomatic SARS-CoV-2” and report findings in more detail to include the meta-regression analyses that Reviewer #5, comment 3 requested. |
| 1. Page7 line 151: ‘Ovid Embase, bioRxiv and medRxiv),’ a comma may be inserted before ‘and’ | This is a decision that we leave to the editors, according to their house style. |
| 2. Page 8 line 184: “The extracted variables included, the study” coma may be removed. | This is a decision that we leave to the editors, according to their house style. |
| **Reviewer #5** |  |
| This is an interesting and useful systematic review and meta-analysis on the occurrence and transmission potential of asymptomatic and presymptomatic SARSCoV-2 infections. The methods including the search, study selection and data extraction, risk of bias assessment, synthesis of the evidence using meta and ggplot2 packages in R and random-effects models are mostly adequate. However, there are still a few major issues needing attention. |  |
| 1. As the authors said in conclusion "this review does not provide a summary estimate of the proportion of asymptomatic SARS-CoV-2 across all study designs". With very wide interquartile ranges (IQR) in most of meta estimates which is largely due to extremely high heterogeneity between studies, it basically demonstrated that the current meta-analysis in this paper doesn't work or the way the evidence was synthesed may not be adequate or practical. With the overwhelming differences in attributes/factors in these 94 studies includes, it is extremely challenging and ambitious to do a meaningful meta analysis. Another substantial limitation in this study is that there is no information on Covid variants or vaccination status of paticipants, which may have a big impact of heterogeneity of the meta results and subsequently led to inconclusive results and findings. The sub group analyses showed some better and narrow IQRs which might indicate a direction of future work in meta-analysing these studies. One of useful features of this study is that authors identified quite a few difficulties, challenges and practical issues in conducting the meta-analysis on asymptomatic infections, however it would be better if the authors could go further (e.g., some relatively neat sub group meta analysis, or extra meta regression analysis) and to be constructive with more in depth and critical discussions to shed some lights in the tunnel. | Thank you. We agree with this summary of the limitations of the study. We hope that our responses to Reviewer #1, comment 3 and Reviewer #2, comment 2 address the comment about variants of concern. We reply to the request for additional meta-analyses with our response to comment 3, below. |
| 2. Prediction interval. Prediction intervals were frequently used in the paper. However this measure is not standard in meta-analysis. I can see prediction intervals were calculated using the 3 previous meta-analyses results but not clear how they were calculated exactly. Basically, as this is a key element of this study, we need a detailed paragraph explaining what this prediction interval is for and what it means in practical terms and how it is relevant to the the summary estimate of meta-analysis. So far, these extremely wide prediction intervals are basically saying no to all the meta analysis results, but the quesion is, is it valid or appropriate doing so? | Thank you for the opportunity to give further explanation about the use of prediction intervals. Whilst they are often not reported, they are recommended by the Cochrane Collaboration (https://training.cochrane.org/handbook/current/chapter-10#section-10-10-4-2) and are available in standard meta-analysis software packages. We added to the Methods (p10, Synthesis of the evidence) “We calculated 95% prediction intervals for all summary estimates, to give a likely range of proportions that would have been obtained in hypothetical studies conducted in similar settings [17,19,20].” |
| 3. To go deeper in finding out the factors contributing to heterogeneity and wide IQR in meta results, I would suggest that authors to carry out some extra meta regression analyses to identify which factors have impact on meta results, e.g., time, age, region, ethnicity, and different study designs and settings so that can give some solid suggestions for future research on this topic. | Thank you for this suggestion. We have now conducted meta-regression analyses (Methods p10, Synthesis of evidence, lines 248-249), “We used meta-regression to examine differences by study design and setting, as well as for post-hoc subgroups of age and geographic region, and with study size and publication date.” We report the findings (Results, p14, Factors associated with proportion of asymptomatic SARS-CoV-2) and now include the Tables, which replace the previous Table 2. |
