# Supplementary material for: Occurrence and transmission potential of asymptomatic and presymptomatic SARS-CoV-2 infections: Update of a living systematic review and meta-analysis
Source: PLoS Med. 2022 May 26;19(5):e1003987. doi: 10.1371/journal.pmed.1003987 (PMC9135333; doi:10.1371/journal.pmed.1003987)
Supplement: Attachment — Submitted filename: Replytocomm_asymptomaticreview_minor_220412_upload.docx [file pmed.1003987.s018.docx]

# Authors’ response to editors’ and reviewers’ comments on version R2

Thank you very much for the opportunity to submit a minor revision. We include a point-by-point response to the editors’ and reviewers’ comments. We include quotations of text that we have changed, referring to page and paragraph numbers, or line numbers of the text in the clean version of the manuscript.

Of note, we have made an explicit statement that we will not do further updates of this living systematic review because we think that the results are unlikely to become more precise. We clarify this in our response to Reviewer #1. This is in line with the ‘stopping rule’ in our protocol.

## Comments from editors

1. Please check the version numbers for consistency, e.g., "v4" at Github, "fifth update" at line 161 and "first three versions" in the acknowledgements.

*Authors’ response:*

*Github: we have renamed this link as “v5”;*

*Line 161: we have renamed this as “fifth version”;*

*Acknowledgements: this was correct – we have clarified the context by adding, “Without their contributions, we would not have been able to complete the fourth and fifth versions.”*

2. At line 83 (abstract), should that be "0.95"?

*Authors’ response: Apologies, yes – corrected.*

3. In table 1, should that be "sex" rather than "gender"?

*Authors’ response: Yes, we have changed this.*

4. At line 270, please make that "8 studies" and use this general style throughout, although numbers should be spelt out at the start of sentences.

*Authors’ response: We have used ‘find and replace’ and used numbers instead of words, except at the beginning of a sentence.*

5. At line 396, please revisit "0.38 95% 0.16-0.64", which appears inconsistent with the numbers quoted in the abstract, for example.

*Authors’ response: Thanks for pointing this out. The correct risk ratio is 0.32 and we have replaced this in line 120 (Author summary) and main text (line 399).*

6. Please revisit reference 26, which may contain a superfluous close bracket.

*Authors’ response: Thanks – bracket removed.*

7. Please remove the asterisks from reference 50.

*Authors’ response: We have corrected this – it is a study group name, “COVID-19 NH-Study Group”.*

8. Please make that "JAMA" in reference 71 and any other instances; and "PLoS ONE" in reference 134.

*Authors’ response: We have corrected the journal names – all other instances are correct.*

9. Please make that "US" in reference 92; and "UK" in reference 191.

*Authors’ response: We have corrected these.*

10. Please check the journal name in reference 109.

*Authors’ response: We have corrected this to “PM&R” – the journal is published by Wiley. The abbreviation stands for Physical Medicine & Rehabilitation.*

11. Should "MFM" be removed from reference 153?

*Authors’ response: MFM is part of the journal title – according to Elsevier, “AJOG MFM is one of two companion titles to the highly-respected American Journal of Obstetrics and Gynecology, and focuses on the latest research in the specialty of maternal-fetal medicine, or high-risk pregnancy.” (https://www.sciencedirect.com/journal/american-journal-of-obstetrics-and-gynecology-mfm/about/aims-and-scope).*

## Comments from Reviewers:

### Reviewer #1:

Excellent review that I hope will be published soon.

I still have a concern about claiming this as a living review. It sets expectations from an end user perspective and as this will likely only be published a year after the end date of the last search.

But leave it to the authors if they think this is important.

Authors’ response: We understand the reviewer’s concern. We would like to retain the term ‘living systematic review’ and we hope we have justified this in our discussion of the limitations.

We have made a change to the conclusion, which says that there is limited value in continuing updates, based on the existing search strategy and evidence base. We have made an explicit statement that we will not do further updates in lines 94-5 (Abstract), 128 (Author summary) and 518-19 (end of Discussion).

### Reviewer #3:

The authors have satisfactorily addressed my previous concerns. To facilitate replication of results in the future, my only remaining suggestion is to include study characteristics described in Table 1 to individual studies listed in Table S2, including the study design, number of index cases excluded (if any), study region and age range of study participants.

Authors’ response: We have added the requested information to S2 Table so that readers can see how individual studies were categorised in Table 1. We have also added a spreadsheet to the Github repository.
